# Genomic and Transcriptomic Insight of Giant Sclerotium Formation of Wood-Decay Fungi

**DOI:** 10.3389/fmicb.2021.746121

**Published:** 2021-10-12

**Authors:** Shuo Cao, Yang Yang, Guiqi Bi, David Nelson, Sheng Hu, Nokwanda Pearl Makunga, Bin Yu, Xin Liu, Xiaohua Li, Xuebo Hu

**Affiliations:** ^1^Laboratory of Natural Medicine and Molecular Engineering, College of Plant Science and Technology, Huazhong Agricultural University, Wuhan, China; ^2^National and Local Joint Engineering Research Center for Medicinal Plant Breeding and Cultivation, Wuhan, China; ^3^Hubei Provincial Engineering Research Center for Medicinal Plants, Wuhan, China; ^4^Wuhan Unique Gene Bioinformatics Science and Technology Co., Ltd., Wuhan, China; ^5^Department of Microbiology, Immunology and Biochemistry, University of Tennessee, Memphis, TN, United States; ^6^Hubei Cancer Hospital, Wuhan, China; ^7^Department of Botany and Zoology, Stellenbosch University, Stellenbosch, South Africa; ^8^Beijing Genomics Institute, Chinese Academy of Sciences, Beijing, China; ^9^State Key Laboratory of Agricultural Genomics, BGI-Shenzhen, Shenzhen, China

**Keywords:** *Wolfiporia cocos*, genome, comparative genomics, sclerotium, sclerotium expanding, medicinal fungi

## Abstract

Many fungi form persistent and dormant sclerotia with compact hardened mycelia during unfavorable circumstances. While most of these sclerotia are small in size, *Wolfiporia cocos*, a wood-decay fungus, grows into giant sclerotia, which are mainly composed of polysaccharides of linear (1→3)-β-D-glucans. To explore the underlying mechanism of converting sophisticated wood polysaccharides for biosynthesis of highly homogenized glucans in *W. cocos*, we sequenced and assembled the genome of a cultivated *W. cocos* strain (WCLT) in China. The 62-Mb haploid genome contains 44.2% repeat sequences, of which, 48.0% are transposable elements (TEs). Contrary to the genome of *W. cocos* from North America, WCLT has independently undergone a partial genome duplication (PGD) event. The large-scale TE insertion and PGD occurrence overlapped with an archeological Pleistocene stage of low oxygen and high temperature, and these stresses might have induced the differences in sclerotium due to geographical distribution. The wood decomposition enzymes, as well as sclerotium-regulator kinases, aquaporins, and highly expanded gene families such as NAD-related families, together with actively expressed 1,3-β-glucan synthase for sclerotium polysaccharides, all have contributed to the sclerotium formation and expansion. This study shall inspire further exploration on how fungi convert wood into simple glucans in the sclerotium of *W. cocos*.

## Highlights

Sclerotia, usually in round, harden mycelia with a diameter of a few millimeters in size, are a form of fungi dormancy to adverse circumstances. However, as a widely used medicinal fungus, *Wolfiporia cocos* in China forms into larger sizes of sclerotia than most other sclerotia-forming fungi. Genomic sequencing revealed that the fungi from China contain significant transposable elements with a partial genome duplication of 1 Mb, as well as a difference in copy number of gene families associated with nutrient and viability, which resulted from a divergent evolution upon environmental stresses that drove the sclerotia formation.

## Introduction

Forming a sclerotium is of utmost importance for fungi to survive and maintain their life cycle in various adverse environments. *Wolfiporia cocos* (Schw.) is a synonym of *Poria cocos* F.A. Wolf, which is a fungus of *Polyporaceae*, *Polyporales*, *Agaricomycetes*, and *Basidiomycota* (Zhao et al., 1998). *W. cocos* forming into a giant sclerotium, an ideal object for studying the formation and development of sclerotium, has been extensively used as a traditional Chinese medicine (TCM) for over 2,000 years in China and other Asian countries (Wang W. et al., 2015). The pharmacological effects of the sclerotium include tumor suppression (Chen et al., 2010), immune enhancement (Wu et al., 2016), and anti-inflammatory activity (Li et al., 2015), and the biologically active compounds of *W. cocos* are mainly concentrated in triterpenes and polysaccharides. Also as a brown rot fungus, *W. cocos* secretes enzymes that effectively decompose cellulose and lignin, which have profound effects on the planetary biomass recycling and bioenergy production (Baldrian and Valásková, 2008).

Giant sclerotium phenomena appear sporadically in *Agaricomycetes* and *basidiomycetes* species such as *W. cocos*, *Grifola umbellata* (Pers.), *Omphalia lapidenscens* Schroet., *Xylaria nigripes* (KI.) Sacc, and *Pleurotus tuber-regium* (Fr.) Sing (Huang, 1999). For example, *P. tuber-regium* forms a sclerotium with a diameter up to 30 cm before a fruiting body grows out of it (Sánchez, 2010). The sclerotium of *Grifola frondosa*, an edible fungus of *Polyporales*, is about the size of a potato (Han et al., 1994). Contrary to the above saprophytic fungi, many phytoparasitic fungi produce a much smaller sclerotium with a diameter of a few millimeters, including species of *Sclerotinia*, *Rhizoctonia*, and *Botryotinia*, with *Sclerotinia sclerotiorum* and *Botrytis cinerea* as examples (Smith et al., 2015).

The formation of the giant fungal sclerotium includes tissue differentiation and morphological changes (Wong and Cheung, 2008). It is known that the sclerotium of *W. cocos* is composed of polysaccharides of linear (1→3)-β-D-glucan (Wang et al., 2004). Polysaccharides isolated from the sclerotium of *P. tuber-regium* were identified as a triple-helix conformation with a main chain of (1→3)-β-D-glucan and branching at every third glucose by a (1→6)-β-D-glucopyranosyl unit (Lin et al., 2020). Previous research on sclerotium formation mostly focused on plant diseases-related small sclerotia fungi, disclosing that the sclerotium formation was closely related to adversity-induced dormancy (Willetts and Bullock, 1992; Smith et al., 2015). The underlying molecular events were also explored. A tetrameric pheromone module SteC-MkkB-MpkB-SteD regulated sclerotia formation in filamentous fungi *Aspergillus flavus* (Frawley et al., 2020). AoRim15, a serine–threonine kinase in *Aspergillus oryzae*, was a positive regulator of the formation of sclerotia and stress tolerance (Nakamura et al., 2016). In *Tuber melanosporum* Vittad., aquaporin-related major intrinsic proteins (PFAM00230) showed the highest transcription level in ectomycorrhizas (Martin et al., 2010).

Previously, the genome sequence of *W. cocos* was sequenced together with a group of lignin-decaying fungi for tracing the paleozoic origin of lignin-degrading peroxidases (Floudas et al., 2012), but it was assembled based on short reads of the next-generation sequencing using a strain from the United States. Here in this study, a strain of *W. cocos* for giant sclerotium production in China was sequenced with a combination of Illumina and PacBio sequencing platform, and then a chromosome-level genome map was constructed in order to uncover the mechanism of sclerotium expansion. We performed the Hi-C-assisted genome sequence assembly, annotation, comparative genome analysis, and transcriptome sequencing to shed light on the genetic strategies for sclerotium formation.

## Results

### Genome Sequencing and Assembly

**FIGURE 1:**
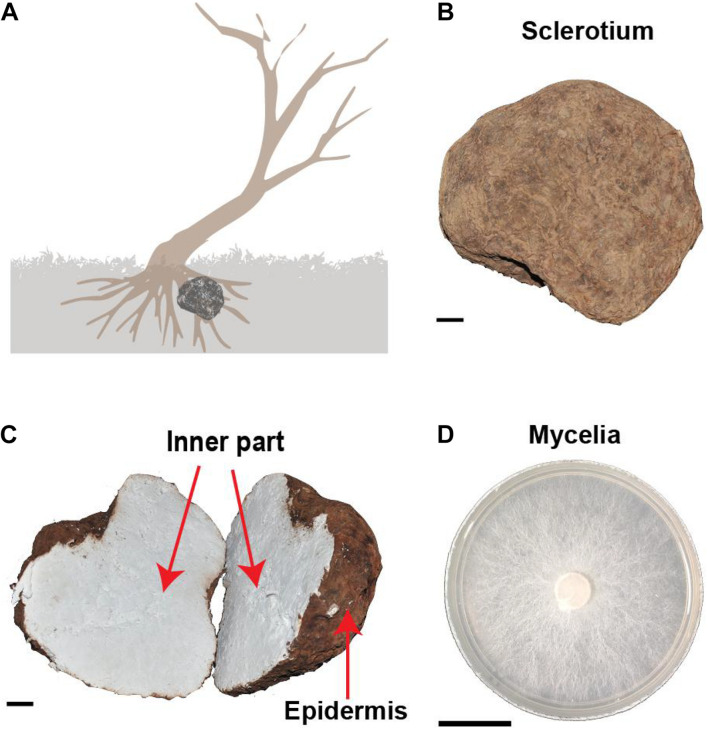
The morphological characteristics of *Wolfiporia cocos*. **(A)** The depicted wild *W. cocos* grows underneath a dead tree in the natural environment. **(B)** A sclerotium of cultured *W. cocos*. **(C)** The cut image of *W. cocos* sclerotium showing two distinct parts of the epidermis and the inner part. **(D)** The mycelium of *W. cocos* grows in a plate. Bar, 2 cm.

The life cycle of *W. cocos* includes three stages of mycelium, sclerotium, and fruiting bodies with the dominant stage of sclerotium development. Naturally, sclerotium grows around the root of a dead pine tree under the soil (Figure 1A). Within a year of inoculation of the fungi mycelium to the wood logs of pine trees, large sclerotia (Figures 1B,C) generally grew and were harvested for medicinal uses in China (Figure 1D). The epidermis of *W. cocos* sclerotium is a continuous layer of tightly packed hyphal tips that become thick walls with dark pigments to form an impervious outer layer.

**FIGURE 2:**
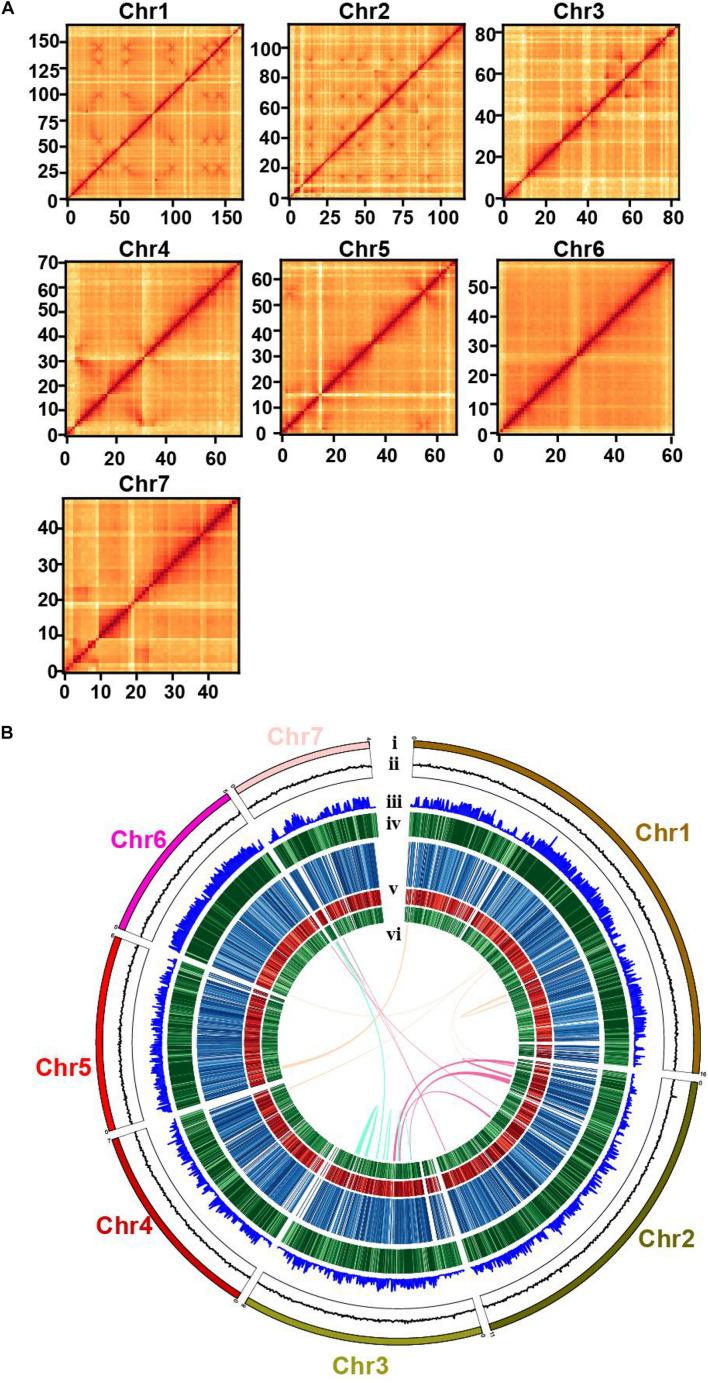
The genome map and chromosomal features of *Wolfiporia cocos* genome. **(A)** A Hi-C map shows genome-wide all-by-all interactions between chromosomes. **(B)** (i), seven chromosomes of the genome of a cultivated *W. cocos* strain (WCLT); (ii), GC content; (iii), gene density; (iv), transposable element (TE) content; (v), gene expression levels in the mycelia, inner part of the sclerotium, and epidermis of the sclerotium from outside to inside. The color deepens as the expression level increases; and (vi), syntenic links.

Previously, a wild strain of *W. cocos* MD104 (sampled in Florida, United States; named WCFL hereafter) was sequenced, and a draft genome with large fragments was assembled, together with other fungi for analyzing wood decomposition (Floudas et al., 2012). In order to comprehensively identify genetic components related to sclerotium formation, we sequenced a cultivated strain of *W. cocos* 2018LT001 (sampled in Luotian, Hubei, China; named WCLT hereafter). We first determined the chromosome number of this strain using the germ burst method (Mehrabi et al., 2012), which clearly showed 14 chromosomes of diploid WCLT under microscopy (Supplementary Figure 1). After sequencing by Illumina and PacBio platforms, we estimated the WCLT genome size to be 62 Mb (in haploid) using 31-nt k-mers (Marçais and Kingsford, 2011). We also conducted Hi-C analysis to assist the genome assembly. The genome features are shown in Table 1. We depicted a Hi-C heat map to separate distinct regions on seven different chromosomes and draw a draft of the haploid genome (Figure 2). The predicted WCLT genome contains 11,906 predicted protein-coding genes. The BUSCO assessment shows that 96.9% of the fungal core genes can be found in the assembled *W. cocos* genome, indicating a high genome integrity (Simão et al., 2015). Long terminal repeat (LTR) assembly index (LAI) is a parameter used for assessing the quality of genome assembly, indicating the ratio of the complete LTR transposon sequence to the length of the total LTR sequence (Ou et al., 2018). In general, the LAI score for draft quality is less than 10, 10 to 20 for reference quality, and LAI greater than 20 indicating gold quality. The LAI values of the seven chromosomes were between 21.64 and 29.06 (Supplementary Table 1), meaning that the sequence assembly is of high quality.

**TABLE 1:** The features of the *Wolfiporia cocos* genome.

Parameters	
Number of chromosomes	14
Estimated genome size (Mb)	62
Total length of scaffold (Mb)	74
Number of scaffolds	145
Longest scaffold (kb)	4,699.2
N50 of scaffolds (kb)	1,599.1
Anchored to chromosome (Mb)	61.127
No. of predicted protein-coding genes	11,906
Average gene length (bp)	1,332.76
Masked repeat sequence length (Mb)	34.5
Percentage of repeat sequences (%)	46.6
GC content (%)	51.86%

With an average sequencing depth of 2,208×, the mitochondrial genome of WCLT was assembled into a circular map of 136,065 bp with GC content of 27.0% (Supplementary Figure 2). It is worth noting that as most conserved and important genes, cytochrome c oxidase (COX) showed unique structures in WCLT. We compared the mitochondria of *W. cocos* with other *Basidiomycetes* mitochondrial genomes. As a result, it was identified that COX1 has two deletions, one 23-amino acid (aa) deletion and, the other, 11-aa deletion; COX2 has a 5-aa deletion (Supplementary Table 2), which may be related to the intron homing (Haridas and Gantt, 2010). However, the evolutionary analysis did not show big differences from the classical fungi nomenclature since COX1 and COX2 are commonly used for DNA barcoding for phylogeny (Supplementary Figures 3A–D).

### Comparative Genome Analysis of Unusual Genomic Structure of *Wolfiporia cocos*

A large number of repeated sequences were found in WCLT, accounting for 46.6% of the whole genome (Table 1). The TE elements annotated in the WCLT genome were mainly LTR transposon sequences dominated by Gypsy. The total LTR complement in WCLT is 8,713, of which 5,686 were Gypsy and 216 were Copia (Supplementary Figure 4). We estimated that the time of LTR insertion was concentrated between 0 and 1 million years ago (Supplementary Figure 5).

**FIGURE 3:**
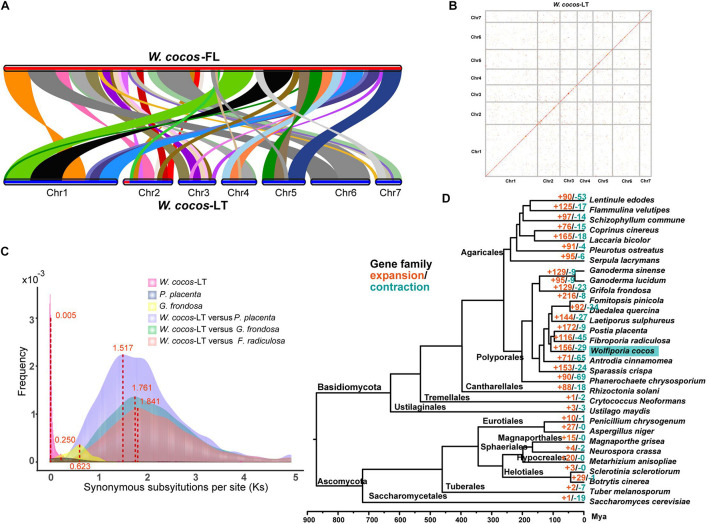
Comparative genomics analysis of *Wolfiporia cocos* and other fungi. **(A)** A colinear block comparison between WCLT and WCFL genomes. **(B)** The synteny plot within the WCLT haploid genome. The red dots indicate potential homology between genes. **(C)** Synonymous nucleotide substitutions (Ks) among a homologous gene pair, showing Ks distribution within and between genomes. The probability density of Ks was estimated by the “density” function in the *R* language. **(D)** A phylogenetic tree of 31 fungi. A total of 210 single-copy genes of 31 fungi species were selected to construct the phylogenetic tree. The red and green color numbers, respectively, represent the number of gene families that expand and contract. Due to lack of fossil data to estimate the specific time, the divergence times were estimated according to the fungal divergence time provided by the TimeTree website (www.timetree.org). Mya, million years ago.

In addition to the long-term geographical differences, we believe that there are some other differences between the domesticated WCLT and the wild WCFL. We compared the homologous regions between 348 scaffolds of the WCFL and seven chromosomes of WCLT (Figure 3A and Supplementary Figure 6) by McscanX (Tang et al., 2008). Strikingly, a 1-Mb segment at chromosome 2 in WCLT did not match any corresponding regions in WCFL. Of 242 genes deduced from the segment, 218 had no homologs in WCFL (Supplementary Data 1). GO annotations indicated that these 242 protein-encoding genes were mainly for catalytic activity, cellular process, and metabolic process (Supplementary Figure 7). Interestingly, 146 of these 242 genes were predicted without any specific functional domains, meaning that these genes might be relatively newly evolved compared with regular genes (Supplementary Data 1). Of all the 22 chitin synthases in the WCLT genome, 8 were in this segment, suggesting that WCLT has a significantly large number of chitin synthase genes.

Whole-genome duplication (WGD) events widely occur in biological systems, and it is one of the most important driving factors for genome evolution, new species origin, and gene functions (Van de Peer et al., 2017). To study the evolution of the *W. cocos*, we searched for whole-genome duplication (WGD) in our assembled *W. cocos* genome and genome of *Postia placenta*, *Fibroporia radiculosa*, and *Grifola frondosa* downloaded from NCBI. The protein sequences from various genomes were searched against themselves by blastp (E < 1e−5) to identify syntenic blocks. In addition, the protein sequences from *W. cocos* were searched against *Postia placenta*, *Fibroporia radiculosa*, and *Grifola frondosa*, respectively. A whole-genome dot map was drawn by MUMmer (Marçais et al., 2018) to show the homology within the WCLT genome (Figure 3B). The syntenic block searching and homology analysis of the WCLT genome indicated that a total of 555 genes were duplicated in 51 syntenic blocks with a total length of 8.6 Mb (Supplementary Data 2). Considering the length of the much large genome size, we think it exhibited a partial genome duplication (PGD).

The occurrence time of the PGD event in the *W. cocos* was estimated. The synonymous substitutions per site (Ks) is widely used to estimate the time of large-scale replication in evolutionary biology. We used TBtools (Chen et al., 2020) to calculate the synonymous substitutions per site of each homologous gene pair and then plot the distribution of Ks values (Figure 3C). One obvious peak at approximately 0.005351042 was observed in the *W. cocos* genomes, and the peak of *P. placenta*, *G. frondosa*, *W. cocos versus P. placenta*, *W. cocos versus G. frondosa*, *W. cocos versus F. radiculosa* is 0.250, 0.623, 1.517, 1.761, and 1.841, respectively. To estimate the occurrence time of the PGD event in *W. cocos*, the calculated Ks value was converted to the divergence time according to formula T = Ks/2r, where r represented a substitution rate of 7.38 × 10^–9^ per site mutations per year (the r was calculated by a comparative genome of 31 fungi genome), which presumably gave WCLT a latest genome-wide doubling event occurring approximately 359,000 years ago according to the calculated evolutionary rate. However, the WCFL genome did not show obvious evidence of PGD event. The GO function annotations indicated that the function of WCLT PGD-related genes are enriched in catalytic activity, cellular process, and metabolic process (Supplementary Figure 8). In particular, multiple genes from the cytochrome P450, glycoside hydrolase family, and glycosyltransferase family have doubled, which might be related to the synthesis of various terpene compounds and polysaccharides in *W. cocos.* In addition, multiple genes of nicotinamide adenine dinucleotide phosphate NAD(P)-binding protein had also been doubled, which are known to be widely associated with biochemical processes of living cells.

The orthologous gene families of the WCLT with 30 other fully sequenced fungal genomes were compared by OrthoMCL (Li et al., 2003), and then the gene family expansion and contraction was analyzed by CAFE (De Bie et al., 2006). A systematic and temporal evolutionary tree was constructed showing the evolutionary relationship of these 31 fungi and their gene families with expansion and contraction (Figure 3D and Supplementary Data 3). It showed that WCLT was grouped into a clade with *Fibroporia radiculosa*, *Postia placenta*, *Daedalea quercina*, *Fomitopsis pinicola*, and *Laetiporus sulphureus*. The major gene families involving the expansion in *W. cocos* are zinc finger transcription factors and protein kinase C superfamilies (Supplementary Data 4). Furthermore, these three gene families also showed extremely significant upregulation in the sclerotium of *W. cocos* according to our transcriptomic sequencing data. It implies that these families may play a unique role in *W. cocos* sclerotium development.

### The Molecular Mechanism of the Mating System in *Wolfiporia cocos*

The mating system of *W. cocos* has not been fully determined previously. We observed no obvious clamp connection in the sequenced strain by microscopy (Supplementary Figures 9, 10). Five protein-coding genes (*WC00116*, *WC07956*, *WC07958*, *WC10813*, and *WC06039*) in the MAT-B locus containing pheromone B alpha receptor and a pheromone-mating factor (STE3) domain were identified. Furthermore, we found no neighboring and divergently transcribed HD genes, implying that *W. cocos* should be a bipolar heterogeneous combination fungus. This is consistent with a previous study (James et al., 2013).

### Transcriptome Analysis

We performed the transcriptome of WCLT in three different tissues: mycelium not form sclerotium (MYC), inner part of sclerotium (IP), and epidermis of sclerotium (EP). Among 10,885 genes expressed in the WCLT genome, 10,152 genes were expressed simultaneously, and 135, 88 and 46 genes were specifically expressed in MYC, IP, and EP parts (Supplementary Figures 11A,B). Pathway analysis of multiple comparisons of MYC, IP, and EP (Supplementary Figures 12A–C) showed that the sclerotium of WCLT had a large number of DEGs enriched in transmembrane transport (GO:0055085) and integral component of membrane (GO: 0016021).

We looked into the differences of secondary metabolism during the fungus development. The metabolic pathways of DEGs (Supplementary Figures 11C,D, 13A–C) were indicated in the biosynthesis of secondary metabolites, metabolic pathways, and starch and sucrose metabolism, which indicated that the membrane activity and active substance synthesis had undergone important changes during the development from mycelium to sclerotium.

Combined with conserved domain analysis, it is clear that transcriptional regulator ICP4 (PHA03247), major facilitator superfamily (MFS), cytochrome P450, PKc like, GAL4, short-chain dehydrogenases/reductases (SDR), PHA03307, fungal transcription factor middle homology region (TF-MHR), PcbC, SLC5-6-like_sbd, WD40, and abhydrolase superfamilies show most significantly differential expression in IP vs. MYC or EP vs. MYC (Supplementary Data 5), and among them, GAL4, fungal-MHR, SLC5-6-like sbd, and PHA03247 superfamilies were mostly upregulated and without downregulation. Because these gene families are associated with gene and protein regulation, chemical modification, and transport, it indicated that they actively participate in the process of sclerotium development.

### Nutrition Utilization and Substance Synthesis

**FIGURE 4:**
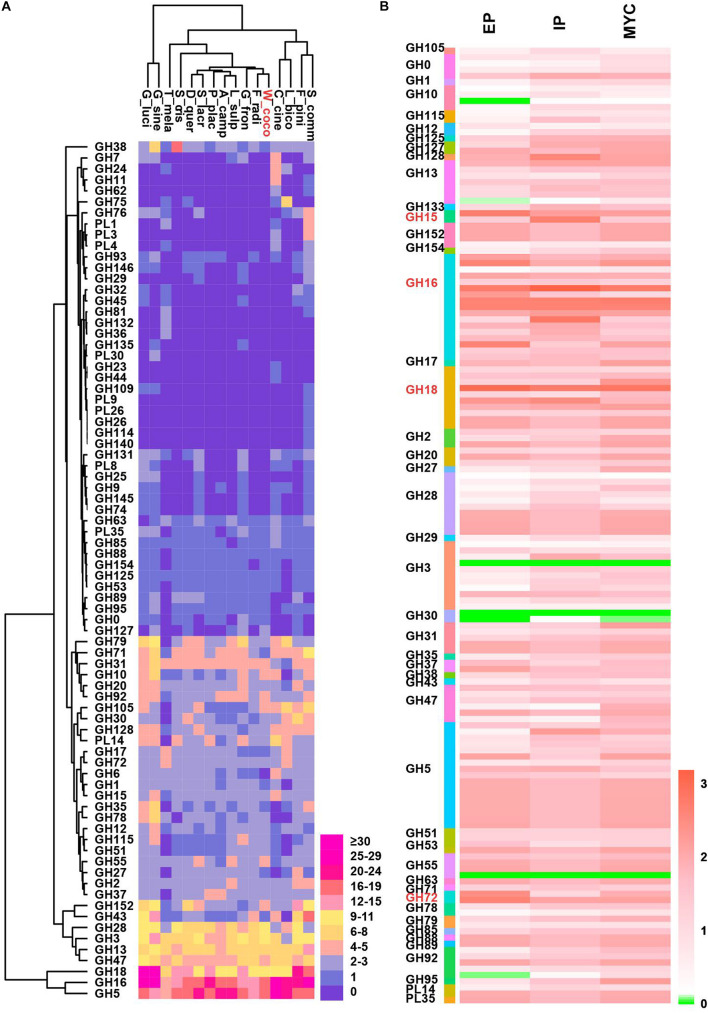
The double clustering glycoside hydrolase (GH) and polysaccharide lyase (PL) families of CAZymes from representative wood-rot fungi genomes and the CAZymes expression level in WCLT. **(A)** The depicted double cluster of GH and PL families in representative wood-rot fungi genomes. The fungi named are *Schizophyllum commune* (S_comm), *Fomitopsis pinicola* (F_pini), *Laccaria bicolor* (L_bico), *Coprinus cinereus* (C_cine), *Wolfiporia cocos* (W_cocos), *Fibroporia radiculosa* (F_radi), *Grifola frondosa* (G_fron), *Laetiporus sulphureus* (L_sulp), *Antrodia camphorata* (A_camp), *Postia placenta* (P_plac), *Serpula lacrymans* (S_lacr), *Daedalea quercina* (D_quer), *Sparassis crispa* (S_cris), *Tuber melanosporum* (T_mela), *Ganoderma sinense* (G_sine), and *Ganoderma lucidum* (G_luci) from right to left. Abundance of the different enzymes within a family is represented by a color scale from 0 (blue) to 30 occurrences (red) per species. **(B)** GH and PL family gene expression in the WCLT genome.

During the formation of sclerotia, considerable nutrients are absorbed from the matrix and decomposed by various enzymes (Wong and Willetts, 1974); the insoluble carbohydrates accumulated in the mycelium during vegetative growth are converted into soluble forms (Jennings, 1987). Also, the size of sclerotia is determined by the nutritional status of the substrate (Willetts, 1971). How *W. cocos* acquires sufficient nutrient for the growth of giant-sized sclerotia is worthy of exploring. Degradative enzymes play a vital role in lignin degradation, pumping a continuous source of carbohydrates and nutrients necessary for giant sclerotium formation. Carbohydrate-active enzymes (CAZymes) that are responsible for breaking down of glycoconjugates, oligo and polysaccharides were analyzed. Of 331 CAZymes annotated from WCLT genome, there are 154 glycoside hydrolases (GH), 79 glycosyl transferases (GT), 3 polysaccharide lyases (PL), 44 carbohydrate esterases (CE), 49 auxiliary activities (AA) families, and 2 carbohydrate-binding modules (CBM) (Figure 4A). The CAZymes of *W. cocos* were compared with a group of wood rot basidiomycetes fungi. The total number of CAZymes in WCLT or WCFL was comparably less than in other fungi, indicated by the CAZymes numbers per Mb of genome (Table 2). A unique profile of WCLT is that no GH6 enzymes were found, while all other fungi had one to six copies. The lack of GH6, less CAZymes, less CBMs is a feature of brown rot fungi. Another difference was that the CMB protein, which belongs to a set of proteins targeting enzymes to promote catalysis and specificity, was significantly less in WCLT than the other fungi studied in this work (Supplementary Data 6). The gene expression of all CAZymes was also analyzed, of which, GH15, GH16, GH18, and GH72 in EP and IP were much higher than in MYC (Figure 4B). The gene patterns and activities of CAZymes might be an important factor contributing to the giant sclerotium development for providing nutrients to WCLT for efficient decomposition of the wood.

**TABLE 2:** The carbohydrate-active enzymes and the subclasses in different wood-rot basidiomycetes fungi.

Species	AA	CBM	CE	GH	GT	PL	Total CAZymes	CAZymes ratio^a^
*WCLT*	49	2	44	154	79	3	331	4.47
*WCFL*	43	3	13	138	61	4	262	5.18
*Fibroporia radiculosa*	41	10	37	142	72	3	305	10.75
*Antrodia camphorata*	33	9	36	135	75	1	289	8.99
*Sparassis crispa*	37	9	39	164	89	5	343	8.79
*Laetiporus sulphureus*	59	12	44	154	83	4	356	8.92
*Postia placenta*	54	11	52	152	82	6	357	8.40
*Laccaria bicolor*	59	12	36	166	86	8	367	5.66
*Serpula lacrymans*	48	7	52	177	77	6	367	8.58
*Daedalea quercina*	51	12	55	173	83	3	377	11.52
*Grifola frondosa*	87	10	37	172	83	8	397	10.10
*Fomitopsis pinicola*	57	12	61	218	84	4	436	10.48
*Ganoderma lucidum*	99	14	52	259	82	11	517	11.94
*Schizophyllum commune*	85	24	67	249	93	18	536	13.93
*Coprinus cinereus*	130	33	75	202	91	16	547	15.11
*Ganoderma sinense*	120	18	65	311	78	11	603	12.32

*^*a*^The CAZymes ratio indicates the value of the total CAZymes per 1-Mb genomic size. AA, auxiliary activities; CBM, carbohydrate-binding modules; CE, carbohydrate esterases; PL, polysaccharide lyases; GT, glycosyl transferases; GH, glycoside hydrolases.*

**FIGURE 5:**
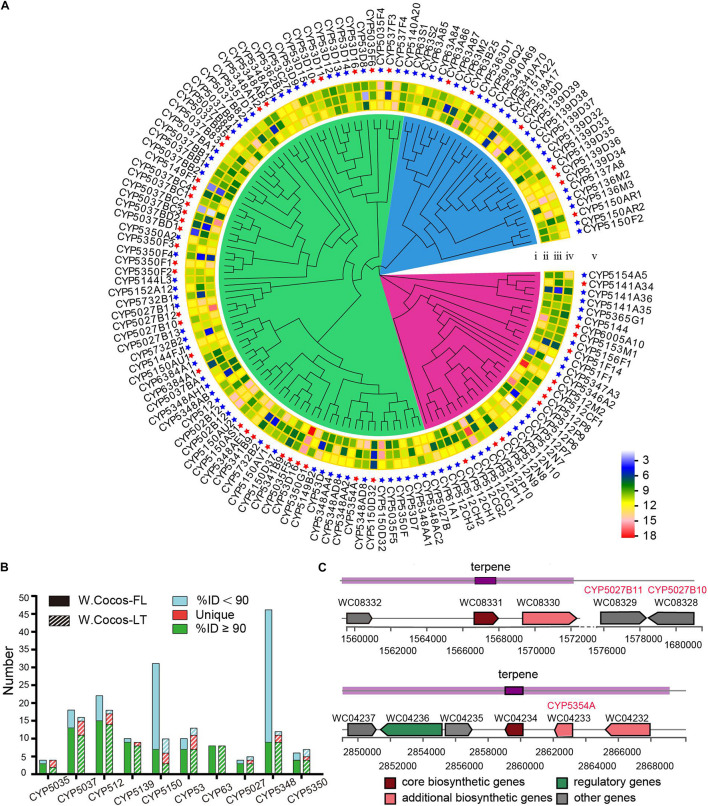
Key genes for triterpene biosynthesis in *Wolfiporia cocos.* **(A)** A phylogenetic tree and gene expression of CYP450 gene in WCLT. (i), the phylogenetic tree of CYP450 genes in WCLT; (ii), the gene expression in EP; (iii), the gene expression in IP; (iv), the gene expression in MYC; and (v) the name of CYPs. The red star indicates the gene coexpression with LSS gene. **(B)** Top 10 CYP450 gene families in the WCLT and WCFL genomes. **(C)** A diagram of representative terpenoid synthetic gene clusters and related CYP450 genes.

Polysaccharides, accounting for over 84% of the weight of dry sclerotium in *W. cocos*, are mainly composed of linear β-(1-3)-D-glucan (Wang et al., 2004). We identified two 1,3-β-glucan synthase-coding genes (*WC02118*, *WC04098*) and one cell wall α-1,3-glucan synthase (*WC01971*); these three genes showed the highest expression in the epidermis of sclerotium, which corresponds to the enrichment of polysaccharides in the epidermis. We also identified one glucokinase (*WC02709*), one phosphoglucomutase (*WC04166*), and two UTP-glucose-1-phosphate (*WC05152* and *WC05158*) coding genes. Eight genes contain β-glucan biosynthesis-associated protein SKN1 domains (*WC00019*, *WC04387*, *WC04388*, *WC09549*, *WC09965*, *WC09967*, *WC09969*, and *WC09971*) and three of them (*WC00019*, *WC09969*, and *WC09971*) encoding β-glucan synthesis-associated protein KRE6. These genes are involved in the biosynthesis of polysaccharides. Because sclerotia is a dormant structure, the corresponding gene and protein activities are expected to be low in it. Thus, it was found that the transcription level of ATP synthase gradually decreased during the transition (Supplementary Table 3). Besides polysaccharides, triterpenoids are known to be major bioactive constituents of *W. cocos* sclerotium, and these chemicals are synthesized through the isoprenoid pathway (Chappell, 2002). The genes for the lanosterol biosynthesis pathway were predicted through NR and KEGG analysis (Supplementary Table 4). A previous study disclosed that the types and content of triterpenoids in the EP were significantly higher than those in the IP and MYC (Jin et al., 2019). It may be closely related to the significantly high expression of triterpene biosynthetic pathways. So far, 84 triterpenoids have been identified in *W. cocos* (Zhang N. et al., 2019), which were variants of lanosterol skeletons, mostly modified by the cytochrome P450 superfamily (CYP). In total, 145 CYP genes were found in the WCLT haploid genome, and this was divided into 42 families through the nomenclature of the CYP International Nomenclature Committee (Nelson, 2006) (Supplementary Data 7). We predicted 203 CYP450 genes from WCFL (Supplementary Data 7). The majority of CYP differences between WCLT and WCFL came from two families: CYP5150 and CYP5348 (Figure 5B). The gene expression level from transcriptome was estimated for all 145 CYPs found in WCLT (Figure 5A). As a result, the expression of 51 CYPs was highly correlated with lanosterol synthase (LSS) (Supplementary Table 5), a key biosynthesis step that is important in forming various kinds of cyclic triterpenoid skeletons, indicating the potential connection between these 51 CYPs and the biosynthesis of triterpene compounds. The expression of all predicted CYPs were verified by qRT-PCR, and it showed that the expression of most genes in the EP was significantly higher than that in IP and MYC (Supplementary Figure 15).

Biosynthetic gene clusters in WCLT were analyzed by antiSMASH (Medema et al., 2011) (Supplementary Figure 16). A total of 18 terpene synthase gene clusters were found (Supplementary Data 8). It is worth noting that CYP5027B10 (*WC08328*) and CYP5027B11 (*WC08329*) were close to the terpene synthesis cluster 1 on chromosome 3; CYP5354A (*WC04233*) was located on another cluster on chromosome 7, and both were co-expressed with LSS, indicating that it is most likely playing a role in the catalysis of terpenoids (Figure 5C).

### Sclerotium Expansion

The sclerotium formed in most fungi is small, but a medium-sized sclerotium of *W. cocos* weighs about 2 kg. The growth ability of a giant sclerotium is very different from that of other fungi. Under the microscope, a large number of hypha complexes tightly wrap to form a dense hard tissue. When the tissue is scattered, it exhibited irregular vacuole-like protrusions; the diameter of the hypha was over twice as those from the mycelium from a culture dish (Supplementary Figure 17).

Stress response signals generated by stimuli from the external environment may initiate the formation of sclerotia. We have identified two stress response-related Dnaj (HSP40; *WC03260*, *WC05954*) encoding genes, which act primarily by stimulating the ATPase activity of heat shock protein 70 (HSP70), both of which had very significantly upregulated expression in IP and EP. Two HSP70 (*WC00798* and *WC00797*) and one heat shock factor protein (*WC02888*) were significantly highly expressed in *W. cocos* sclerotium compared with those of the mycelium, which indicated that the high expression of stress-related signals might regulate the initiation of the sclerotium formation of *W. cocos*. MAPK signaling is necessary for the pheromone response of the conservative cAMP signaling pathway (Saito, 2010). Most of the genes in the MAPK pathway are differentially expressed in the EP, IP, and MYC of *W. cocos*. Furthermore, adenylate and guanylate cyclases and an adenylate cyclase regulation G protein was also found significantly highly expressed in the sclerotium.

Previously, a few regulators of small sclerotium formation were identified in filamentous fungi (Nakamura et al., 2016; Frawley et al., 2020). Accordingly, a dozen genes in WCLT were matched to fungi sclerotia regulators (Supplementary Data 9), and among them, an MAPKK gene (*WC01972*) and MPKKK gene (*WC05263*) were showing significantly higher expression in the sclerotium. It was found that aquaporin-encoding genes (*WC00149* and *WC08005*) had significant expression in EP and IP than in MYC, suggesting that the high level of aquaporins might contribute to the viability of the fungi. These actively expressed genes may be related to the continuous growth of sclerotium.

**FIGURE 6:**
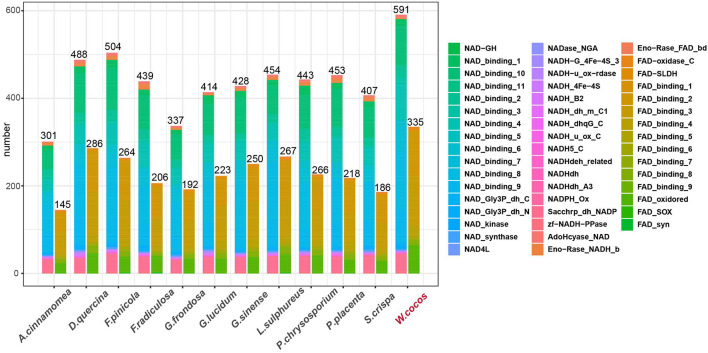
The numbers of nicotinamide adenine dinucleotide (NAD)- and FAD-related domains in WCLT and other representative fungi in *Polyporaceae*. The domains were predicted by Pfam 3.1.0; the right side of the figure indicates different family classifications.

Nicotinamide adenine dinucleotide (NAD^+^) is a cofactor of many metabolic enzymes in living cells, which are closely related to various physiology and pathogenesis (Covarrubias et al., 2020). It is worth noting that the number of NAD and its closely related domains in *W. cocos* is significantly higher than that of the other fungi from *Polyporales* (Figure 6), mainly due to the duplication of these genes during PGD events. Besides, 55.8% of genes containing NAD- and FAD-related domains were significantly differentially expressed in the sclerotium versus the mycelium (Supplementary Data 10).

## Discussion

The dried sclerotium of *W. cocos* is widely used in oriental medicine, with approximately 10% of the TCM formula containing the material according to the record of the Chinese Pharmacopoeia (Chinese Pharmacopoeia Commission, 2010). It is one of the top four TCM material clinically used in China with extremely high economic and medicinal value. In this study, we endeavored to explore the genetic mechanisms that are at play in the giant sclerotium formation. WCLT has been cultivated for over a thousand years, and thus, the long time of breeding optimization might have posed a selection on the mushroom genetics. We performed Illumina and PacBio platform sequencing, together with Hi-C-assisted assembly for a cultivated strain of *W. cocos* PCmonoA, to draft a chromosomal-level genome. Prior to sequencing, we were able to observe the *W. cocos* chromosome numbers under light and fluorescent microscopy by a germ bust method (Mehrabi et al., 2012). The 14 chromosomes from microscopy were consistent with the sequence assembly of the diploid *W. cocos*.

The genome size of the WCLT genome is much larger than that of most other basidiomycetes mainly due to a high ratio of repeat sequences. Interestingly, extremely high TEs content contributed to 125 Mb of black truffle (*T. melanosporum* vittad.) in fungi (Martin et al., 2010). Coincidentally, both *W. cocos* and *T. melanosporum* are giant underground fungi. Any intrinsic connection of these TEs for these two mushrooms is not yet known.

The genome of WCLT studied here exhibited great divergence from that of WCFL from the United States. The genome size of WCLT is much larger than that of WCFL. Although it was stated that the WCFL encoded 12,358 genes (Floudas et al., 2012), data presented here indicates that the WCLT encoded only 11,906 genes. Besides, there was an extra fragment encoding 242 genes in chromosome 2 in WCLT, compared with WCFL. These genes may possibly provide additional growth regulators that control the development of the giant sclerotium for WCLT. For example, this region contained nearly one third of chitin syntheses in WCLT, which is pivotal to the biomass accumulation of fungi. The extra region might be gained by repeatedly screening for a larger sclerotium during the long term artificial cultivation.

The genome-wide scattered gene duplication is another significant difference between WCLT and WCFL. A PGD was predicted to have occurred at a peak around 359,000 years ago for WCLT but not for WCFL. The occurrence of high TEs in the genome was also within a million years ago. Both events might be related to a record of low-oxygen and high-temperature climate cycles that changed between 1,250,000 and 700,000 years ago on the earth (Elderfield et al., 2012). Mushrooms in different geographic locations might have suffered distinct pressures, and this may possibly explain the divergent evolutionary responses. The mushroom close to the ocean (WCFL) or inland (WCLT) might have responded differently to the adverse temperature and oxygen availability. As a result, WCFL gained duplicates of genes for nutrient utilization, such as glycoside hydrolases, glycosyltransferases, and the reaction drivers NAD(P)-binding proteins. These genes may favor forming a giant sclerotium.

Typically, the sexual development of most *Basidiomycetes* involves the mycelium developing into a fruiting body, but for *W. cocos*, its mycelium first grows into a giant sclerotium and then fruit bodies germinate from the mature sclerotia in the natural environment. The reproductive mating type may be relative to the giant sclerotium formation. While previously, the existence of clamp connections and the mating type of *W. cocos* was still controversial (Xiong et al., 2006), mating type in fungi seems not to be consistent with phylogeny. *Ganoderma lucidum* and *Antrodia camphorata* are also fungi of *Polyporales*, but they exhibit a tetrapolar heterogeneous combination without an obvious sclerotium (Adaskaveg and Gilbertson, 1986; Lu et al., 2014). On the other hand, *W. cocos* appears to undergo a bipolar heterogeneous combination. This mating pattern might be beneficial for giant sclerotium in that a bipolar heterogeneous combination than the tetrapolar combination might be more vulnerable to adversities in terms of generation of genetically diverse offspring, thus, forming a sclerotium as a dormancy is an alternative asexual reproduction to better the chance of survival.

The mtDNA genome is a specific and powerful tool for studying the origin and evolution of species (Li et al., 2018). It exhibits characteristics of small molecular weight and rapid sequence variation compared with the nuclear genome (Delsuc et al., 2003). The WCLT mtDNA presents, as a closed circular double-stranded structure, encoding several genes that are fundamental for aerobic metabolism: seven NADH dehydrogenase complex subunit genes, three COX1, one COX2, and three ATP synthase subunit genes. Although a few deletions were found in COX1 and COX2, it did not change the phylogeny of the most conserved proteins during evolution. How these deletions and intron homing affect the functionality of these two proteins remains to be elucidated in the future.

Sclerotiogenesis and development are affected by various factors, including environmental sensing, cellular sensing, and signal transduction. Nutritional factors are essential in the sclerotia formation. Previous studies suggested that the formation of the sclerotium occurs under circumstances of starvation and other adverse conditions that are not suitable for the continuous growth of the mycelium (Willetts and Bullock, 1992), which means the supply of carbohydrates is, thus, essential for the development of the giant sclerotium. Carbohydrate-active enzymes are keys for *W. cocos* to gain nutrition from the wood. Compared with other wood-rot fungi of Basidiomycota, although *W. cocos* has a largest genome, the number of its CAZymes is comparably less than other wood rot fungi (Table 2). Noticeable differences were found as no copy of GH6 and fewer copies of the CBMs were present in *W. cocos.* Also, some GH gene subfamilies presented to have a higher expression in the EP or IP than MYC (Figure 4). On the other hand, the sclerotium is largely composed of linear β-(1-3)-D-glucan, which is catalyzed by 1,3-β-glucan synthase. The high expression of the 1,3-β-glucan synthase and also the cell wall α-1,3-glucan synthase in the epidermis indicate that active biomass is being processed. We speculated that, while maintaining both decomposition enzymes and biosynthetic glucan synthase at a high level for nutrient supply and new substrate synthesis in abundance, *W. cocos* shall keep receiving strong signals to convert wood from one kind of polysaccharides to other ones. The underlying mechanism is awaiting further disclosure.

CYP was much appreciated in medicinal fungal genome studies (Chen et al., 2012; Lu et al., 2014) because it plays a central role in triterpenoid modifications. At present, it is not yet clear why the number of CYP genes in WCLT is one third less than WCFL. This is rather puzzling as fewer CYP indicates less diversity of variants associated with terpenoids and other specialized metabolites. However, it is evident that differences in the composition of secondary metabolites from different geographies prevail, and more especially, this is linked to the two biggest CYP families, CYP5150 and CYP5348.

Aquaporins might be associated with sclerotia development. Two aquaporin paralogous proteins were identified from *W. cocos* and each of them showing a highly differential expression pattern in EP and IP, respectively. In line with over 45% water content of the sclerotia we tested, aquaporin proteins regulate the ingress and egress of water and this is beneficial to more water and biomass content in the sclerotium. To our attention, aquaporin-related proteins exhibited the highest transcription level in ectomycorrhizas in truffle *T. melanosporum* (Martin et al., 2010). Also, fungi aquaporin ΔAQP1 mutants showed a bubble-like mycelium structure (Ding et al., 2018), which is similar to the mycelium morphology of the inner part of the *W. cocos* sclerotium because the mycelium become swollen and vacuolated, and the cells are shortened with thickened walls. Taken together, differentially expressed signaling pathway, stress response protein, and aquaporins may be closely linked to the sclerotium development from cellular morphology, tissue pattern, and size swelling.

NAD^+^ maintains metabolic homeostasis of the oxidation and reduction status and regulation of the longevity of cells (Cantó et al., 2012). The NAD^+^ and its supplements have shown great potential in treating aging-related diseases (Zhang et al., 2016). By comparative genomics study, we found that the number of NAD- or FAD-related domains was more than that of other fungi in *Polyporales* (Figure 6). Furthermore, more than half of the genes containing those domains were differentially expressed in the sclerotium versus mycelium (Supplementary Data 10), indicating that the *W. cocos* has a better anti-aging potential than other fungi in *Polyporales*, which might facilitate its growing faster and longer that lead to a bigger sclerotium.

Signaling pathways of oxidative stress are closely related to the formation of sclerotia. The comparison of transcriptome results at different growth stages indicated that oxidative stress caused by unfavored environment played an important role in the early formation of the sclerotium (Erental et al., 2008). Under a microscope, the mycelia undergo obvious fusion and polymerization, which might be forced to have specific cell-to-cell adhesion to multihyphal structures (Supplementary Figure 14) to cope with the unfavorable growth environment.

Another unique profile of *W. cocos* is that gene families relating to protein regulation, chemical modification, and transportation, such as GAL4 (Gal4-like dimerization domain), fungal TFs (transcription factors), MHR (the major homology region), SLC5-6-like_sbd (sodium/glucose co-transporters solute-binding domain), and PHA03247 superfamilies were significantly upregulated in the sclerotium, compared with those in the mycelium. Among them, GAL4 and fungal TF MHR superfamilies in *W. cocos* showed significant expansion, compared with other fungi genomes. These proteins might play a pivotal role in the formation of giant sclerotium of *W. cocos*.

In conclusion, the full sequencing, assembly, and annotation of *W. cocos* provided valuable and new information on the genomic structure, genetics, evolution, and phylogeny on the giant sclerotium development. The data are pivotal not only for the domestication, cultivation, production, and evaluation of the medicinal properties of a traditional medicine but also provide novel insights to its contribution to the biogeochemical cycles as a wood decomposition fungus in nature and understanding of its unique life style to keep its prosperity in adverse environments.

## Materials and Methods

### Sequencing Material

The *Wolfiporia cocos* was sampled in Luotian County, Hubei Province. The inoculated mycelium grew into a mature sclerotium in about 6 months. The brown epidermis of the mature sclerotium was peeled off, and the inner part sclerotium was cut into pieces. After disinfection, it was inoculated in a plate with potato dextrose agar solid medium to obtain a white mycelium. The strain was named *Wolfiporia cocos* 2018LT001. The mycelium used for inducing sclerotium was used to generate mononucleated protoplasts (Wang Q. et al., 2015). After microscopic examination under a fluorescent microscope, a generated strain denoted as *W. cocos* PCmonoA with lesser nucleus was selected for genome sequencing. More detailed experimental descriptions are provided in the Supplementary Notes.

### Genome Sequencing

DNA (1.5 μg) was sonicated using a Covaris E220 sonicator (Covaris). The fragments were end repaired and 3′-adenylated, and Illumina adapters were added using the Kapa Hyper Prep Kit (Kapa Biosystems, Wilmington, MA, United States). The ligation products were purified with AMPure XP beads (Beckmann Coulter Genomics). The libraries were quantified by quantitative PCR using the KAPA Library Quantification Kit for Illumina Libraries (Kapa Biosystems), and the library profiles were assessed using a DNA High Sensitivity LabChip kit on an Agilent Bioanalyzer (Agilent Technologies). The libraries were sequenced on an Illumina HiSeq2500 instrument (Illumina) using 250-base length read chemistry in paired-end mode. In addition, ∼6.2-G subreads were generated using SMRT sequencing technology on the PacBio RSII platform with an average coverage depth of 100×. The average length of the subreads was 11.2 kb.

### Genome Size and Heterozygosity Estimation

The raw Illumina data were treated using the following steps. First, reads were aligned to the adapter sequences, which were truncated according to alignments. Second, an NGS QC Toolkit (Patel and Jain, 2012) was used to filter low-quality reads by satisfying one of these three conditions: bases with quality ≤ 20 were regarded as low-quality bases, and the percentage of low quality bases in a read was ≥40%, or ambiguous bases’ percentage of a read was ≥10%, or the read length <50 bp. Third, the samtools (Li et al., 2009) platform was used to remove the PCR duplicates. We ran a kmer analysis using Jellyfish (v.2.2.6) (Marçais and Kingsford, 2011) before the kmer frequency was analyzed using the Genomescope (Vurture et al., 2017) software for genome survey (genome size and heterozygosity under kmer = 31). The haploid genome size was estimated to be 62 Mb, and the percentage of the sequences repeated at least twice in the genome was recorded as 36.9%.

### Long-Read Genome Assemblies

We used the wtdbg2 (Ruan and Li, 2020), canu (Koren et al., 2017), DBG2OLC (Ye et al., 2016), miniasm (Li, 2016), Ra^1^, flye (Kolmogorov et al., 2019), and FALCON (Chin et al., 2016) assemblers with all PacBio raw reads for the genome assembly. Then, we selected the best assembly for further analysis, based on contiguity metrics, such as N50 or cumulative size. The FALCON assembler produced the most contiguous assembly (N50 of 1.48 Mb). A high-quality consensus was accessible by polishing the consensus of the selected assembly three times with the PacBio reads as inputs to the Racon (Vaser et al., 2017) software and then three additional times using Illumina reads as input to the Pilon (Walker et al., 2014) tool. Both tools were used with default parameters.

### HiC-Scaffolding

We deduplicated the assembly contigs with the Purge Haplotigs pipeline (Roach et al., 2018) to produce primary contigs of ∼73 Mb as well as contigs labeled as assembly artifacts (180 kb). Then we used the Purge Haplotigs software, calling minimap2 to post the third-generation sequencing reads to the genome sequence and counted the coverage depth of each site, composed of a map, and viewed the third-generation sequencing depth distribution. Furthermore, the haploid sequences were distinguished with 61,098,192 bp and diploid heterozygous sequences of 12,729,286 bp. The selected haploid genome was basically in line with the kmer estimated haploid genome size of the next-generation sequencing data. We then generated scaffolds from the assembly contigs using Hi-C generated read data with ALLHIC (v.0.8.11) (Zhang X. et al., 2019).

### Genomic Repetitive Sequence Prediction

We used RepeatModeler^2^ and RepeatMasker^3^ to build a *de novo* repeat library and shield the repetitive sequence. Tandem repeats were detected in the genome using the software Tandem Repeats Finder (TRF).

### Gene Prediction and Functional Annotation

The total RNA of the hypha and sclerotium of *W. cocos* transcriptome data (SRR768316) was downloaded from the SRA database, and our sequenced transcriptome data of the mycelium that did not form a sclerotium (MYC), inner part of sclerotium (IP), and epidermis of sclerotium (EP) were adopted to perform hisat2 comparison with the gene model. The Bam2hint was used to anchor the exon and intron boundaries, and then BAKER (v1.9) was used for gene prediction. For the integrated gene model, we use BLASTP (Gish and States, 1993) to compare the gene model with the NCBI non-redundant (NR) database and Swiss-Prot database. Protein domains were annotated by searching against the InterPro (Hunter et al., 2009) and Pfam (Finn et al., 2014) databases using InterProScan (Quevillon et al., 2005) and HMMER (Finn et al., 2011). The result of the genome comparison is imported into Blast2Go^4^ for Gene Ontology (GO) annotation. The KAAS online server^5^ was used to compare metabolic pathways using the BBH method (bidirectional best hit) to conduct KEGG annotation. The CDD (Lu et al., 2020) (conserved domains) online server was used to submit multiple batches and CDD annotation. The CYP450 genes in *W. cocos* were predicted using the CYP450 hmm model (PF00067) from Pfam (Tu et al., 2020).

### RNA Extraction, Library Construction, and Transcriptome Sequencing

Three biological replicates of the mycelium (MYC), inner part of the sclerotium (IP), and epidermis of sclerotium (EP) were sampled for transcriptomics analysis. Total RNA was extracted separately from each sample using the RN38 EASYspin plus Plant RNA kit (Aidlab Biotech, Beijing, China). After the total RNA extraction and DNase I treatment, the concentration and quality of each sample were examined using a NanoDrop2000 (Thermo Scientific, Wilmington, DE, United States) and Agilent 2100 Bioanalyzer. The mRNA was isolated by magnetic beads with Oligo (dT) and then synthesized to cDNA. Short fragments were purified and resolved with EB buffer for end reparation and single nucleotide A addition. After that, the short fragments were connected with adapters. Finally, the libraries were sequenced using Illumina NovaSeq 6000.

The raw data produced by Illumina NovaSeq6000 were subjected to FastQC^6^ to evaluate read quality. After filtration of low QC reads and removal of joint contamination by fastp^7^, reads were aligned to the *W. cocos* genome sequences by hisat2^8^. Then the gene and isoform expression analysis was carried out. The differentially expressed genes (DEGs) were selected by a threshold of false discovery rate (FDR) ≤ 0.001 and an absolute log2ratio value ≥1 among the three biological replicates based on the analysis method of the Poisson distribution by DESeq2^9^. Furthermore, the sequences of DEGs were compared with the NCBI non-redundant (Nr) database, Gene Ontology (GO), and Kyoto Encyclopedia of Genes and Genomes (KEGG) databases to identify and annotate the obtained DEGs using the Blast software. In addition, the log2 (folds of mean RPKM values to the 0-h time point) were used to generate cluster diagrams by the MultiExperiment Viewer software with a color scale using the hierarchical clustering method. ClusterProfiler was used to analyze the^10^ enrichment of DEGs based on the GO annotation and KEGG annotation of the genome.

## Data Availability Statement

The WCLT genome project have been submitted to National Genomics Data Center, China National Center for Bioinformation with a Bioproject: PRJCA003291 (https:/bigd.big.ac.cn/gwh/Assembly/10372/show). All other detailed experimental descriptions related in this article can be found in the Supplementary Notes part of Supplementary Material. All of Supplementary Figures and Supplementary Tables are included in Supplementary Material. Supplementary Data 1–12 are provided in Supplementary Dataset.

## Author Contributions

YY, SC, BY, GB, SH, and XH conceived and designed all the experiments. YY, SC, GB, XHL, and XL performed the experiments. SC, YY, BY, GB, SH, DN, XH, and NPM analyzed the data. SC, YY, DN, NPM, and XH wrote the manuscript. All authors contributed to the article and approved the submitted version.

## Conflict of Interest

BY is employed by Wuhan Unique Gene Bioinformatics Science and Technology Co., Ltd. The remaining authors declare that the research was conducted in the absence of any commercial or financial relationships that could be construed as a potential conflict of interest.

## Publisher’s Note

All claims expressed in this article are solely those of the authors and do not necessarily represent those of their affiliated organizations, or those of the publisher, the editors and the reviewers. Any product that may be evaluated in this article, or claim that may be made by its manufacturer, is not guaranteed or endorsed by the publisher.

## Footnotes

1
https://github.com/lbcb-sci/ra

2
http://www.repeatmasker.org/RepeatModeler.html

3
http://www.repeatmasker.org/

4
https://www.blast2go.com/

5
https://www.genome.jp/tools/kaas/

6
http://www.bioinformatics.babraham.ac.uk/projects/fastqc/

7
https://github.com/OpenGene/fastp

8
http://ccb.jhu.edu/software/hisat2

9
https://bioconductor.org/packages/release/bioc/html/DESeq2.html

10
https://bioconductor.org/packages/release/bioc/html/clusterProfiler.html
