# Supplementary material for: Genomic and Transcriptomic Insight of Giant Sclerotium Formation of Wood-Decay Fungi
Source: Front Microbiol. 2021 Oct 12;12:746121. doi: 10.3389/fmicb.2021.746121 (PMC8546338; doi:10.3389/fmicb.2021.746121)
Supplement: Supplementary file 13 [file Table_13.DOCX]

**Supplemental Information**

**Genomic and transcriptomic insight of giant sclerotium formation in fungi**

Shuo Cao^1,2,3*^, Yang Yang^1,2,3*^, Guiqi Bi^4*^, David Nelson^5^, Sheng Hu^6^, Nokwanda P. Makunga^7^, Bin Yu^4^, Xiaohua Li^1,2,3^, Xin liu^8,9^, Xuebo Hu^1,2,3^*

^1^ Laboratory of Natural Medicine and Molecular Engineering, College of Plant Science and Technology, Huazhong Agricultural University, Wuhan, 430070, China;

^2^ National & Local Joint Engineering Research Center for Medicinal Plant Breeding and Cultivation, Wuhan, 430070, China;

^3^ Hubei Provincial Engineering Research Center for Medicinal Plants, Wuhan, 430070, China;

^4^Wuhan UniqueGene Bioinformatics Science and Technology Co., Ltd., Wuhan, 430070, China

^5^Dept. of Microbiology, Immunology and Biochemistry, University of Tennessee, Memphis TN 38163, USA;

^6^Hubei Cancer Hospital, Wuhan, 430079, China;

^7^Department of Botany and Zoology, Stellenbosch University, Stellenbosch, South Africa;

^8^BGI-Beijing, Beijing 100101, China;

^9^State Key Laboratory of Agricultural Genomics, BGI-Shenzhen, Shenzhen, 518083, China;

***Correspondence** X.H. (xuebohu@mail.hzau.edu.cn).

Address: Room A104, Complex III, 1 Shizishan, Nanhu, Wuhan, 43007, China

Telephone number: 0086-180-6250-3982.

**Running title**: *Wolfiporia cocos* sclerotium formation mechanism

**SUPPLEMENTARY Methods**

**Production and regeneration of *W. cocos* protoplasts**

*Wolfiporia cocos* cultivar strain was sampled in Luotian County, Hubei Province, China,The protoplast monokaryogenesis method^1^ was used and some improvements were made. Mycelia were inoculated into 100 ml PDA liquid media (200 g potato, 20 g dextrose, 1 L water) in 250 ml flasks. The mycelium was cultured in the dark on a shaker at 150rpm/min, 28℃. when the mycelia grows to strong viability, put the rotor into the flask and place it on a magnetic stirrer to break up the mycelia to improve the efficiency of enzymolysis. The cultures were harvested by filtering through sterile gauze and washed by 0.6 M mannitol solution; prepare 3.5% lywallzyme ( Guangdong Institute of Microbiology, China) using 0.6 M mannitol solution and the lywallzyme solution is sterilized through a 0.22 micron sterile filter membrane, add lywallzyme solution as 2 ml of per gram wet mycelia to lyse the cell wall with occasional agitation at 30℃, 3 h. Hyphal fragments were removed by filtration through a column of cotton wool packed up to the 0.4 ml mark of a 5-ml syringe. Protoplasts were collected from the effluent by centrifugation (1400 x g, 10 min) and washed twice by 0.6 M mannitol solution, centrifuge again, Dilute the volume to 1ml with 0.6 M mannitol solution and count number by hemocytometer. Dilute the protoplast suspension to 1 × 10^4^ cells/ml and add 50 μl to a MYG medium ( 10.0 g malt extract, 4.0 g glucose, 4.0 g yeast extract, 20.0 g agar and distilled water to 1 L) plate and inoculated at 28°C in the dark constant temperature incubator.

MYG medium set a control prepared with distilled water instead of mannitol to test whether the cell wall is completely removed. (If the medium prepared with water also produces clones, it indicates that the enzymolysis is incomplete.)

**Selection and validation of** **Monokaryotic strain**

After about a week or longer of growth, the clones visible on the culture medium were picked and transferred to the PDA medium. If the regeneration rate is very low, adding 1% glycine to the MYG medium can have a good effect. We observe the number of nuclei of the hypha through fluorescence microscope, In order to better observe the hyphae, we Insert three coverslips into the culture medium obliquely to induce hyphae from each colony to climb on the slide. We choose hoechet 33258 solution (50 μg/ml, dissolve in Disodium hydrogen phosphate-citrate buffer, PH=7.2) to stain mycelium in case the DAPI and PI can’t stain very well. In the dikaryotic strain W.cocos-FL, no clamp connections were observed and the mycelium has no messy branches. However, we also did not observe obvious clamp connection but there were a large number disorder branches.

In order to further determine the monokaryotic strain, we used flow cytometry to detect the fluorescence degree of individual cells of different strain. 150 mg of wet hyphae were placed in an ice bath petri dish, add 2 ml OttⅠ solution (0.1M Citric acid monohydrate, 0.5% v/v Tween 20, filtered by 0.22 μm filter membrane), use a razor blade to quickly chop the hyphae and pass through a 300 mesh nylon screen , collect the filtrate into a 1.5 centrifuge tube, centrifuge at 8000 rpm for 5 min, and then remove the supernatant. Make up to 100 μl with OTT1 solution and shake gently,add 50 μl RNAse (1mg/ml) to react for 5 minutes and then add 50 μl PI solution to stain for 10 minutes in the dark, Filter through a 300-mesh nylon sieve into another 1.5ml centrifuge tube, and add 400 μl OttoⅡ solution(0.4 M Na_2_HPO_4_·12H_2_O filtered by 0.22 μm filter membrane). The result of flow cytometer is shown as Supplementary Figure S14.

**Chromosome observation of *W. cocos* monokaryon strain**

The chromosome number and morphology of W.cocos have not been clarified so far. In this paper, the number and morphology of chromosomes were observed. Because the filamentous fungal mycelium are slender and the cells are generally tiny, it is difficult to observe their complete chromosomes under light microscopy using ordinary tableting methods, so the Germ burst method was used to observe the chromosomes ^2^. The mycelial growth tip usually contains several dividing nuclei, and when ruptured, the concentrated chromosomes are expelled from the mycelial cells to spread onto the surface of the slide.

Due to the inconvenience of collecting spores under laboratory conditions, hypha fragments are used for chromosome observation. Inoculate the seed liquid culture into the liquid medium(35 g/l dextrose, 5 g /l peptone, 5 g/l Yeast extractand, 1g/l Potassium Dihydrogen Phosphate, 0.5 g/l Magnesium Sulfate Heptahydrate) grow at 28℃ for 3 days. Mycelial fragments are resuspended in liquid medium. A glass slide is immersed in the poly-L-lysine solution（1 mg/ml）to coat the glass slide with poly-L-lysine. Shake gently and stand still for 5-10 minutes. Use rubber cement glue to make a rectangular frame on the glass slide. Pipette 200ul of resuspended hypha fragments to rectangular frame on glass slide and incubate in the dark for 10 hours, humidity 88%. Gently suck the liquid from the surface of the glass slide, do not let the slide dry. Gently pipette 200μL of liquid medium containing thiabendazole (TBZ) at a concentration of 50μg/mL to rectangular frame to prevent metaphase mitosis. After incubating for 3 hours at room temperature, gently aspirate the TBZ solution. Use fine tweezers to remove the rectangular frame, wipe off excess water with filter paper. Gently immerse the slide in the freshly prepared fixative (methanol: glacial acetic acid 17:3) for 20 minutes. Dry the slide quickly on the flame of an alcohol lamp. Prevent excessive drying or overheating. Then put the slides into an ultra-clean workbench with air circulation to completely dry them. Drop 25 μL dye solution containing 2 μg/mL DAPI and PI onto the slide, and do not touch the surface of the slide. Soak up excess liquid and gently place the coverslip on the slide and keep it in the dark for 15 minutes. Use nail polish to seal around the glass slide. Place the prepared slides under a fluorescence microscope or a confocal laser microscope to obtain a higher resolution image as much as possible.

**Observation of the morphology of *W. cocos***

*Wolfiporia cocos* cultivar strain was sampled in Luotian, Hubei of China. Through continuous separation and purification, sterile white *W. cocos* mycelium were obtained, and the mycelium were cultured on a petri dish to observe the hyphae morphology. The mycelium is used as an inducement to inoculate the pine tree section, and it is buried in the soil for artificial cultivation. After six months, the sclerotium of *W. cocos* can be collected.

The appearance of mycelium is white fluffy, with unique multi-concentric ring colonies. The sclerotium is a dormant body formed by a large number of mycelium tightly packed and entangled, which is spherical, elliptical, oblate or irregular. The weight varies from hundreds of grams to tens of kilograms. The epidermis of the sclerotium is shell-like, with rough surface and nodular shrinkage

When fresh, it is light brown or tan, and after drying, it becomes dark brown. The fruiting bodies are produced on the surface of the sclerotium under natural conditions, and can be obtained by culturing aging mycelium under laboratory conditions.

The fresh whole sclerotium used in this article weighs 2214.5 g, the inner part water content is 45.24%, Epidermis water content is 45.26% and Mycelia water content is 58.85%.

We cut open the whole sclerotium, and take a small piece of it to observe the internal sclerotium morphology through a stereo microscope. Spread the internal tissues on the glass slide as much as possible and count 100 pieces of normal and sclerotium-forming hypha diameter respectively. The box diagram drawn by GraphPad Prism 7 shows that the average diameter of sclerotium-forming hypha is more than twice that of hypha without forming sclerotium. And the sclerotium-forming hypha has a crystal-like luster, which may be a complex formed with some polysaccharides. Observing it under an optical microscope, we can see a clear vacuole-like structure, with many branches and protrusions, and irregular shapes, which are significantly different from hypha without forming sclerotium.

**Genome assembly**

We calculated the double-ended kmer (31 bp) frequency of the sequencing data, and used the Poisson distribution formula to estimate the size of the fungal genome. Kmer frequency distribution graph shows two main peaks and sequencing depth is 50X and 100X respectively. It shows that there are extremely long diploid fragments in the genome. In addition, the CANU results shows the obviously spliced bubble structure due to diploid hybridization. Therefore, it is concluded that *W. cocos* is diploid. The predicted genome haploid size is 62 Mb. We first use the second-generation data and use SPAdes 3.11.0 for splicing to see the actual size of the genome after the second-generation data is spliced. The results show that the size of the genome spliced by SPAdes is close to that estimated by kmer. Then we spliced the three-generation data. We tried seven softwares including wtdbg2, canu, DBG2OLC, FALCON, miniasm, Ra, and flye for genome splicing. The results show that the genome assembled based on FALCON has the best indicators. N50 reaches 1.48MB, and the longest sequence stitched out reaches 4.68 Mb, which is significantly better than the splicing results of other software. We used FinisherSC to perform three-generation data scaffolding of the genome assembly results, then used the third-generation data and the second-generation sequencing data to perform three genome corrections on the splicing results; finally, using the next-generation sequencing data try to further scaffolding by SOPRA. However, the results show that the next-generation data does not improve the third-generation scaffolding.

**Hi-C scaffolding constructs seven chromosomes of *W. cocos***

The genome size obtained by the third-generation sequencing splicing results is 74 Mb, which is larger than our estimated haploid genome size (62 Mb). Therefore, there is a vesicle structure in the genome due to diploid heterozygosity, and the excised chromosome heterozygous fragments need to be screened and excluded before the subsequent construction of the chromosome sequence.

Use Purge Haplotigs software to post the third-generation sequencing reads to the genome sequence through minimap2, and count the coverage depth of each site, compose the map, and view the third-generation sequencing depth distribution. Set the corresponding depth threshold between the two obvious peaks, and use minimap2 to post the distributed genome sequences of different regions to the genome to further distinguish which are haploid sequences and which are diploid heterozygous sequences. We set 15, 70, and 170 as three depth thresholds on the sequencing depth. Select the haploid genome sequence of Poria cocos for subsequent Hi-C reads comparison. Purge Haplotigs produces the following three files: curated.fasta, Genome haploid sequence; curated.haplotigs.fasta, Diploid heterozygous fragment; curated.artefacts.fasta, Sequences that are too deep or too deep may be contaminated or repetitive sequences in the genome. The selected haploid genome is basically the same size as the kmer estimated haploid genome of the second-generation sequencing data.

For Hi-C clean reads, use HiC-Pro to compare data, filter Hi-C alignment results, detect valid Hi-C sequences, merge results, construct Hi-C correlation maps, and standardize correlation maps. The 16.5Gb original sequencing reads are filtered to produce about 4.5Gb valid data. The specific construction process is as follows：Use the ALLHiC algorithm to divide and sort chromosome groups. The generated intermediate sequence is compared with the filtered Hi-C data through bwa, and the Hi-C file is constructed through the juicer process. Use Juicebox verion1.9.8 to manually correct the sequence. Use HiC-Pro to re-align the filtered data to the corrected chromosome sequence, and use ALLHIC software to draw a Hi-C heat map.

After constructing the seven chromosome sequences, we need to integrate the original gene prediction results with the current seven chromosomes. After trying exonerate to align the predicted gene sequence to the chromosome, it was found that there were a few gene alignment problems (amino acid alignment nucleic acid). After several more attempts, We decided to use the chain.py script in the ALLMAP software to build the chain file. Then use liftOver to adjust and integrate the original gene prediction results with the current chromosome sequence. The assembled *W. cocos* haploid genome covers all the haploid genome sequences selected by purge haplotig. The total number of protein-coding genes covered is 10795, and the number of all protein-coding genes is 11906, which means that there are 1111 genes distributed in the diploid heterozygous sequence.

**Genome integrity assessment**

A set of *W. cocos* transcriptome data (SRR768316) was downloaded from the SRA database, and the data was quality controlled using Trimmomatic. Subsequently, the genome is aligned. Use Hisat2 to perform variable-cutting replies and count the replies of reads to assess the integrity of the genome. The result shows an overall response rate of 90.17%, the integerity is high considering the data from different sample sources.

Use BUSCO^3^ to detect the eukaryotic gene set of unsterilized, sterilized genome sequences and predicted protein coding genes to predict genome integrity. BUSCO results show that whether it is at the genome level or at the level of gene prediction, whether using eukaryotic gene sets or fungal biological core genes, it all shows that the genome integrity is very good, at 96.9%.

**Mitochondrial genome splice**

The mitochondrial genome is spliced using GetOrganelle software. We download the fungal samples it provides, use the default parameters to screen the mitochondrial reads, then splice and assemble it into a ring. Then result was submitted to the website GeSeq for gene annotation.

**Comparison genome of different fungal species**

The whole genome protein sequences of 31 fungal species (*Wolfiporia cocos, Laetiporus sulphureus, fibroporia radiculosa, Postia placenta, Ganoderma lucidum, sparassis crispa, Serpula lacrymans, Antrodia camphorate, Laccaria bicolor, Coprinus cinereus, Schizophyllum commune, Phanerochaete chrysosporium, Crytococcus Neoformans, Ustilago maydis, Lentinula edodes, Pleurotus ostreatus, Daedalea quercina, Fomitopsis pinicola, Grifola frondosa, Ganoderma sinense, flammulina velutipes, Tuber melanosporum, Botrytis cinerea, Sclerotinia sclerotiorum, Rhizoctonia solani, Magnaporthe grisea, Saccharomyces cerevisiae, N**eurospora crassa, Aspergillus niger, Penicillium chrysogenum, Metarhizium anisopliae*) were respectively blast aligned. Then use the orthoFinder^4^ program to identify gene families, and finally select 210 single-copy gene protein sequences to construct a species evolutionary tree. We use RAxML^5^ software to build a maximum likelihood tree through 1000 bootstrap. The PROTGAMMALGX model was selected during the establishment process.

The molecular phylogenetic tree constructed with RAxML software is re-rooted according to the topological structure of the fungal species phylogenetic tree to obtain a molecular phylogenetic tree similar to the phylogenetic relationship of the species. Then determine the differentiation time of the evolutionary tree. Since there is no fossil data to estimate the specific evolutionary time, we estimate the differentiation time of the phylogenetic tree based on the fungal species differentiation time provided by the timetree website, and generate a phylogenetic tree with relative differentiation time.

The orthologous gene families of *W. cocos-LT* genome with other 30 fully sequenced genomes was identified by OrthoMCL^6^. The whole genome protein sequences of 31 fungal species were respectively blast aligned, and then the orthoFinder program was used to identify gene families. We count the number of genes of each species in each family, and then remove families with less than 2 genes in all species. The remaining 9564 gene families are estimated by the CAFÉ^7^ software for gene family expansion and contraction.

CAFÉ software uses the birth-death model to estimate the size changes of the gene family of the two species relative to the nearest common ancestor, and calculates the pvalue to determine whether a gene family has significantly expanded or contracted. We choose the result with pvalue less than 0.01 as the result of significant change. According to the gene family size of the nearest common ancestor of each branch estimated by CAFÉ, we classify the significant gene families into Expansion and Contraction, and mark on the corresponding branches in the molecular evolutionary tree with orange and light green respectively.

**Intergenomic co-linearity analysis of *W. cocos-L*T and the** ***W. cocos-FL***

*Wolfiporia cocos MD-104 SS10* (*W.cocos-FL*) genome^8^ and annotation File were downloaded from NCBI. We artificially integrated all the configs of *Wolfiporia cocos MD-104 SS10* into a large block without change the gene sequence to conduct co-linearity analysis with our assembled *W.cocos-LT* genome. We used the software MCscan (https://github.com/tanghaibao/jcvi/wiki/MCscan-(Python-version)) to compare the CDS sequences of the large block with the seven chromosomes of *W.cocos-LT*, and draw using the software's own mapping tool.

**Mating type genes**

The observation of *W. cocos* mycelium showed that it had no obvious clamp connection, which made it difficult to identify the reproductive mating of *W.cocos*. We identified four MAT-B locus genes (WC00116, WC07958, WC10813 and WC06039) containing both pheromone B alpha receptor and pheromone mating factor (STE3) domains through CDD annotation. What’s more, we found a MAT-A locus with one pair of divergently transcribed HD1(WC04599) and HD2(WC04600) but no linkage to the P/R genes. This may means that *W. cocos* is a tetrapolar heterogeneous combination. However, previous studies have shown that its MAT-A locus cannot be effectively amplified, so it is inferred that it is a bipolar heterogeneous combination^9^.

**CYP450 annotation and nomenclature**

The CYP450 genes in *W. coc*os were predicted using CYP450 hmm model (PF00067) from Pfam. The predicted CYP450 sequences were submitted to the the CYP International Nomenclature Committee for naming.

**The extraction of total RNA of MYC, IP and EP of *W. cocos*** The mycelium is cultivated in the laboratory, and the inner part tissues and epidermis of the sclerotium are cut from sclerotium collected in Luotian, China. *W. cocos* have high polysaccharide content in various tissues. In order to improve the quality of RNA, the extraction process has been optimized. Take out the dry heat sterilized mortar and place it on ice, pre-cool with a small amount of liquid nitrogen and add samples of different tissues. Add liquid nitrogen and quickly grind the sample. All EP tubes and mortars need to be pre-cooled in advance. Put 100 mg of sample powder into a 1.5 ml EP tube with 1ml TRIZOL reagent, add 200 μL of chloroform and then add 60 μL of 2mol/l sodium acetate solution to create a high salt environment to precipitate polysaccharides, and then add 10 μL of β-Mercaptoethanol to prevent Phenols are oxidized, shake vigorously for 5 minutes and incubate at room temperature for 5 minutes. Centrifuge at 12000 rpm for 10 minutes at 4°C. Gently pipet the supernatant into another 1.5ml EP tube. Add the same volume of isopropanol as the supernatant, invert 6 times, and incubate at room temperature for 10 minutes. Centrifuge at 12000 rpm for 10 minutes at 4°C. Discard the supernatant, add 1ml of 75% ethanol, and gently flick the RNA to suspend it. Centrifuge at 2500 rpm for 5 minutes at 4°C. Repeat once the steps of adding ethanol and centrifuging. Leave it on a sterile workbench with ventilation for 10 minutes to dry the RNA and then use 50 μL DEPC water to dissolve RNA.

Use 1% agarose gel ,1 μL RNA, 1 μL loading buffer and make up the reaction sysem to 5 μL with ultrapure water to detect RNA quality by electrophoresis.

Use a nucleic acid protein quantifier to determine the RNA concentration and quality.

**Real-Time Quantitative PCR**

Because a large number of CYP genes have high sequence similarity, it is not accurate enough to define gene expression only through transcriptome short-read sequencing. We performed real-time fluorescent quantitative PCR (qRT-PCR) to verify CYP genes expression. In additional, the expression of genes encoding key enzymes in the lanosterol biosynthesis pathway and speculative giant sclerotium formation regulates gene were verified.

Use 1000 ng RNA, 3 μl gDNA Earser Mix, 4 μl 5*TRUE RT MIX and add ultrapure water to make up to 20 μl. (Operate on the PCR machine) Mix gently RNA, gDNA Earser Mix and ultrapure water, incubate at 42°C for 2 minutes. Centrifuge the reaction solution to the bottom of the tube, add 4μl 5*TRUE RT MIX to the same tube. Mix gently and incubate at 42°C for 20 minutes, and then inactivate gDNA Earser Mix and TRUE RT MIX at 85°C for 5 seconds. Dilute the reverse transcription product and store it at -20℃ for experiment.

25 μl reaction system:12.5 μl 2*SYBR qPCR Mix,1μl DNA template, 0.5 μl Forward Primer (10 μM), 0.5 μl Reverse Primer (10 μM) ,add ddH_2_O to 25 μl.

The PCR amplification program consisted of denaturation at 94°C for 2 min followed by 40 cycles of 94°C for 15 s, 60°C for 20 sec and 72°C for 30 sec, and then 95 ℃ 15 second and 60 ℃ 1 min for dissociation. The HIS-3 gene was selected as the internal reference gene for gene expression data normalization. The relative expression levels were calculated by comparing the Ct (cycle threshold) of the target gene with that of the internal reference gene HIS-3 using the 2^-ΔΔCt^ method. The all gene primers were designed using NCBI Primer-BLAST website online. ( https://www.ncbi.nlm.nih.gov/tools/primer-blast/index.cgi)

**Polysaccharide biosynthesis**

Polysaccharide substance is another active substance with extremely high content in addition to triterpenes in *W. cocos*. The water-soluble 1,3-β- and 1,6-β-glucans are the most active ones for immunomodulatory compounds. We identified two 1,3-beta-glucan synthase coding genes (WC02118.t1, WC04098.t1) and one each of glucokinase (WC02709.t1), phosphoglucomutase (WC04166.t1), two UTP-glucose-1-phosphate coding genes (WC05152.t1, WC05158.t1).

Eight genes contain β-glucan biosynthesis-associated proteins SKN1 (WC00019.t1 WC04387.t1 WC04388.t1 WC09549.t1 WC09965.t1 WC09967.t1 WC09969.t1 WC09971.t1) domain and three of them (WC00019.t1 WC09969.t1 WC09971.t1) encoding Beta-glucan synthesis-associated protein KRE6 were also discovered in the *W.cocos* genome.

**Carbohydrate-active enzymes**

We used CAZy database (www.cazy.org) to identify the carbohydrate-active enzymes family of 16 representative white rot or brown rot fungi, and count the number of genes in each family. A total of 331 *W. cocos* genes could be assigned to carbohydrate-active enzymes (CAZymes) families. This total cover 154 glycoside hydrolase genes contain 12 chitinase (GH18) and 17 cellulose degradation (GH5), 79 glycosyltransferase genes, 49 Auxiliary Activities, 44 carbohydrate esterase genes, 3 polysaccharide lyase genes and 53 carbohydrate-binding genes. The size of this carbohydrate family in *W. cocos* is at a low level among the 16 selected fungi species.

**Biosynthesis gene clusters**

In order to explore the key genes of secondary metabolite biosynthesis. We use antiSMASH fungal version website (https://fungismash.secondarymetabolites.org) to predict the secondary metabolite biosynthesis gene clusters in *W. cocos* genome^10^. We found a total of 39 gene clusters distributed on 7 chromosomes, containing 18 TERPENE gene clusters, 8 T1PKS gene clusters, 12 NRPS-LIKE gene clusters and one INDOLE gene cluster.

**Transport proteins**

The transporter in *W. cocos* was identified online by the Transporter Classification Database (http://tcdb.org/) ^11^(Supplementary Data 10).

**Regulatory protein**

We found 726 genes contain various regulatory protein domains via CDD and Interpro annotations (Supplementary Data 11). A t-cell immunomodulatory protein and 14 velvet-domain-containing proteins were identified in *W. cocos* genome. Proteins containing zinc finger domains such as CCHC-containing proteins, C2H2-containing proteins and Zn2Cys6-containing proteins account for a large portion.

**SUPPLEMENTARY FIGURES**


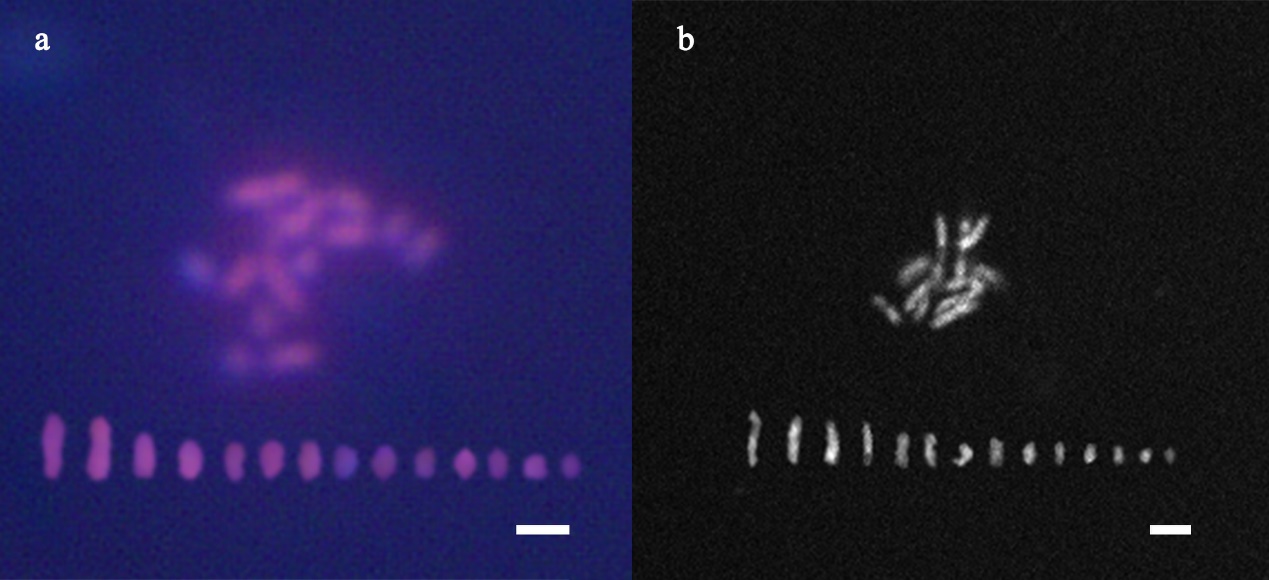


**Supplementary Figure S1. Observation of 14 chromosomes of the diploid *W. cocos* by Germ burst method, stained by 1 μg/mL DAPI and PI solution.** (a) Chromosomes observation by optical fluorescence microscope. (b) Chromosomes observation by confocal laser scanning microscope. Chromosomes are roughly depicted and arranged in size below the picture. Bar, 2 μm.


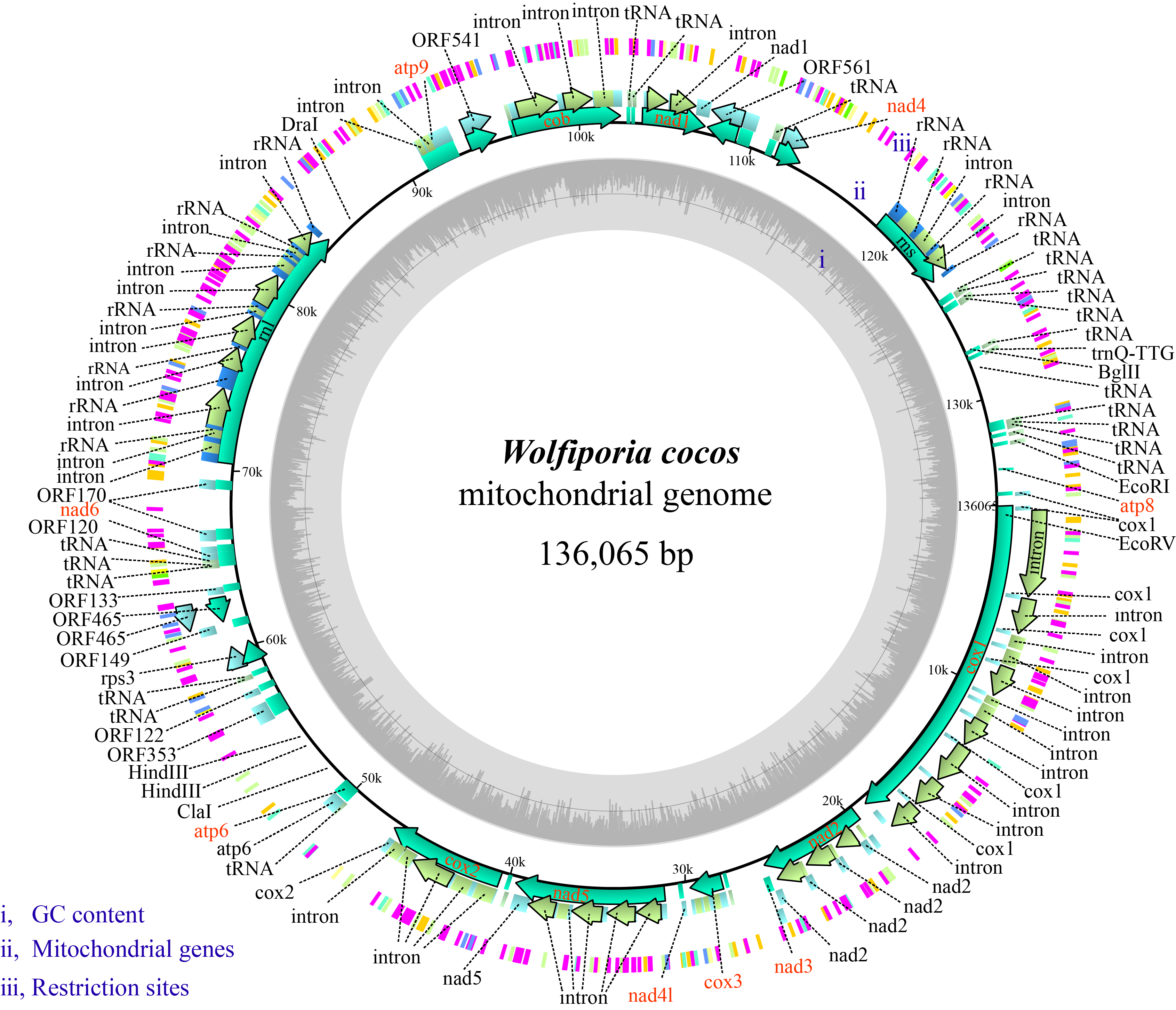


**Supplementary Figure S2. The mitochondrial genome of *W. cocos*.** ⅰ, GC content in different sites of circular mitochondrial genome. ⅱ, Mitochondrial genes, different sites are labeled. ⅲ, Restriction sites.

**
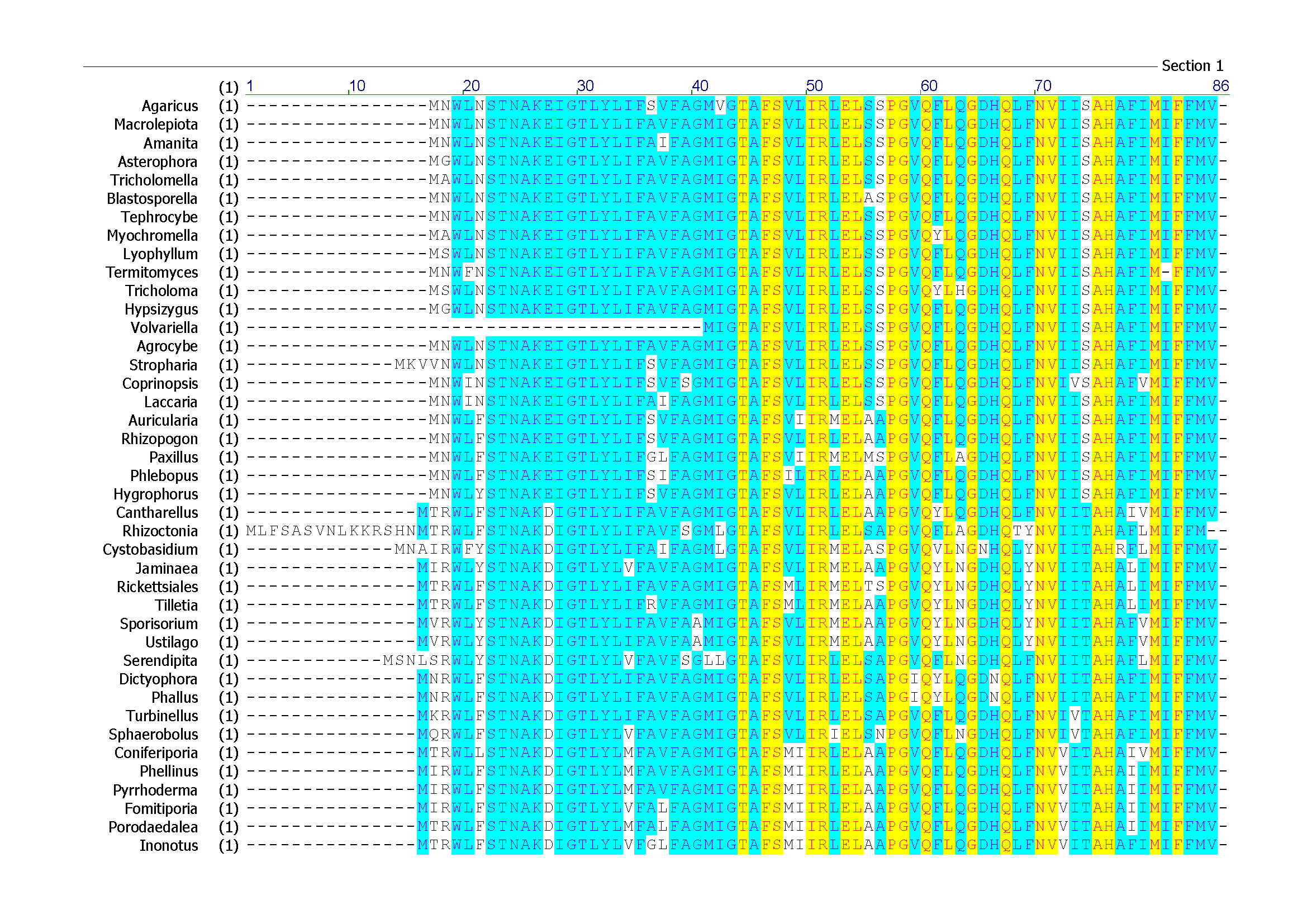
**

**
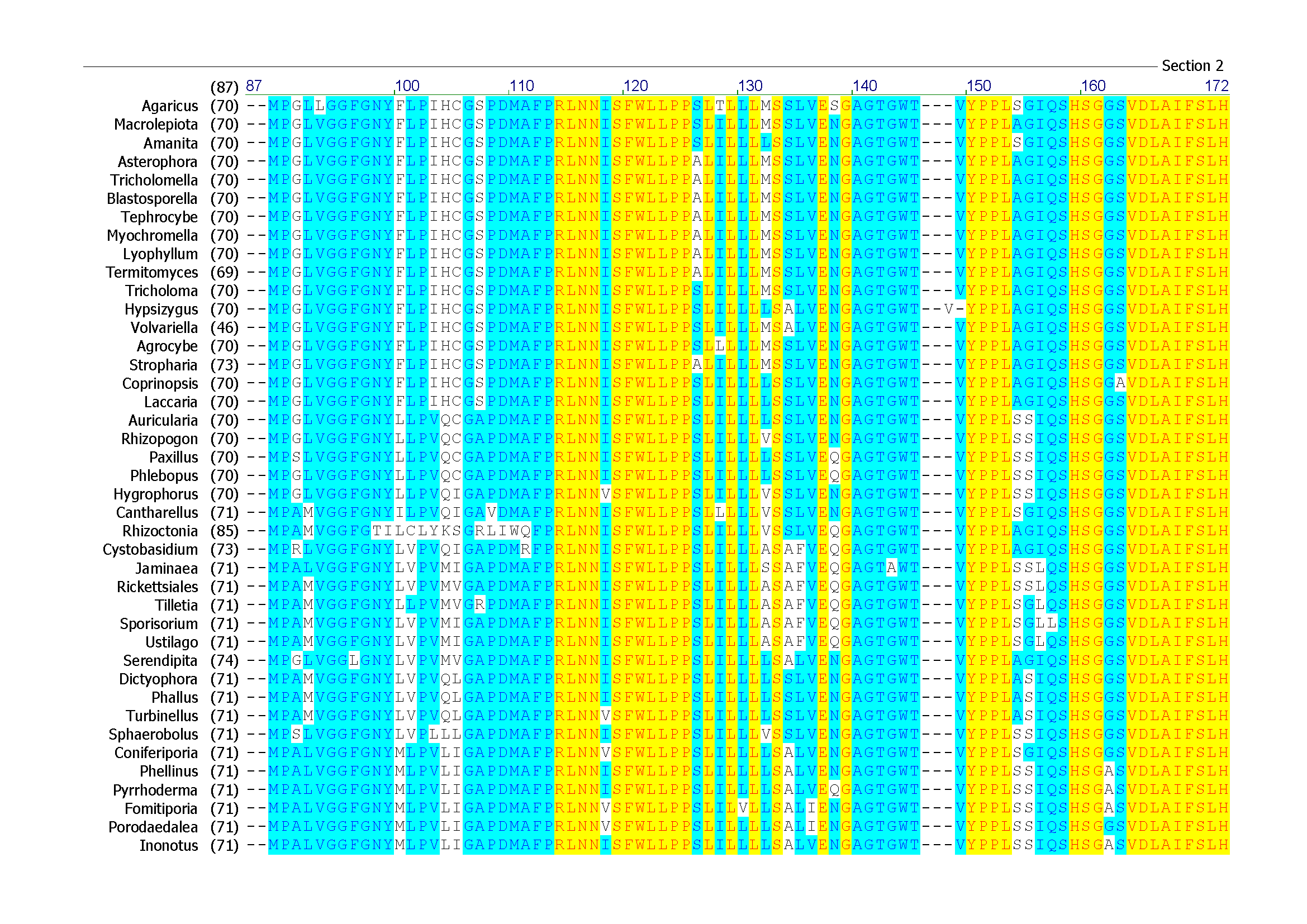
**

**
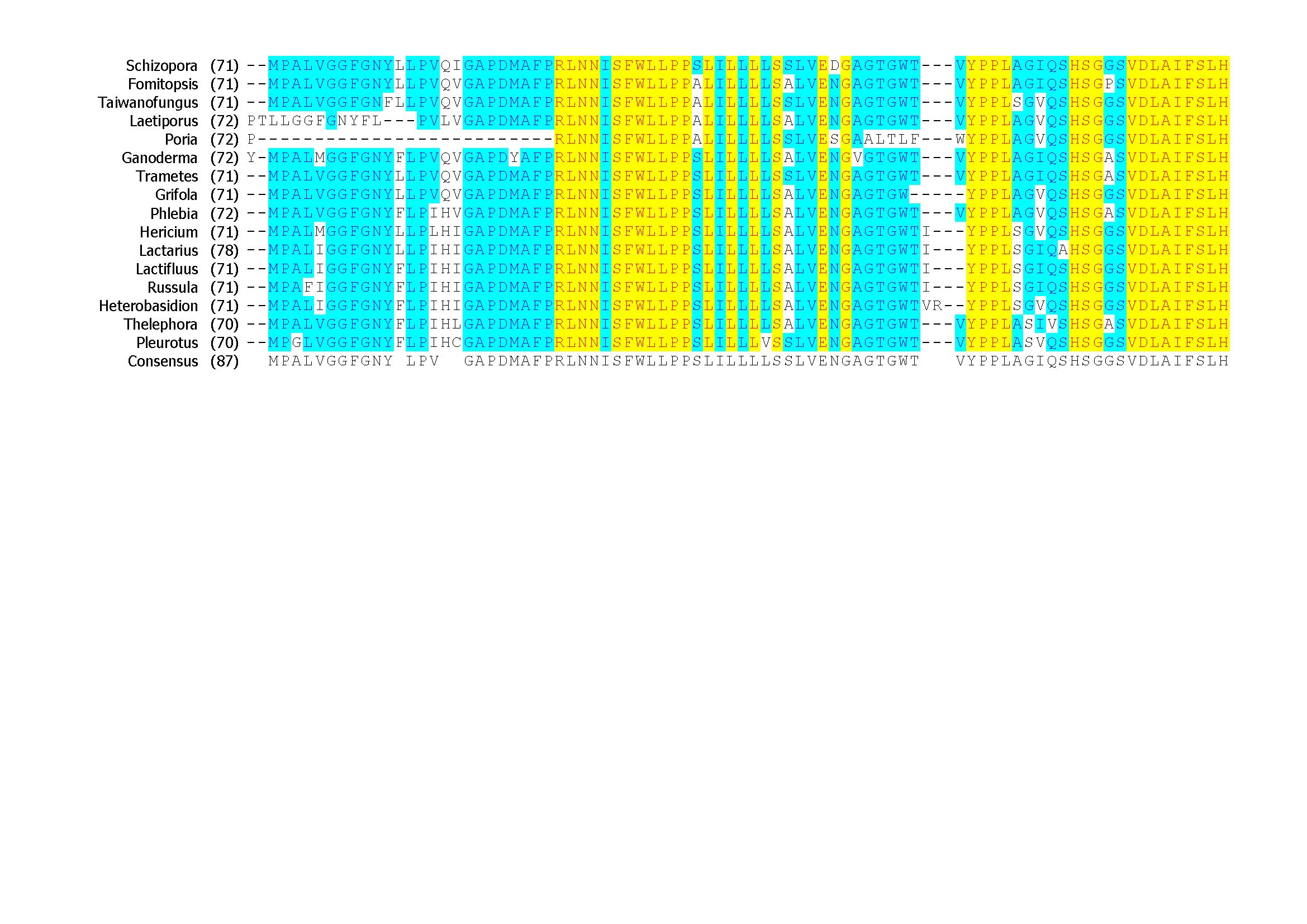
**

**
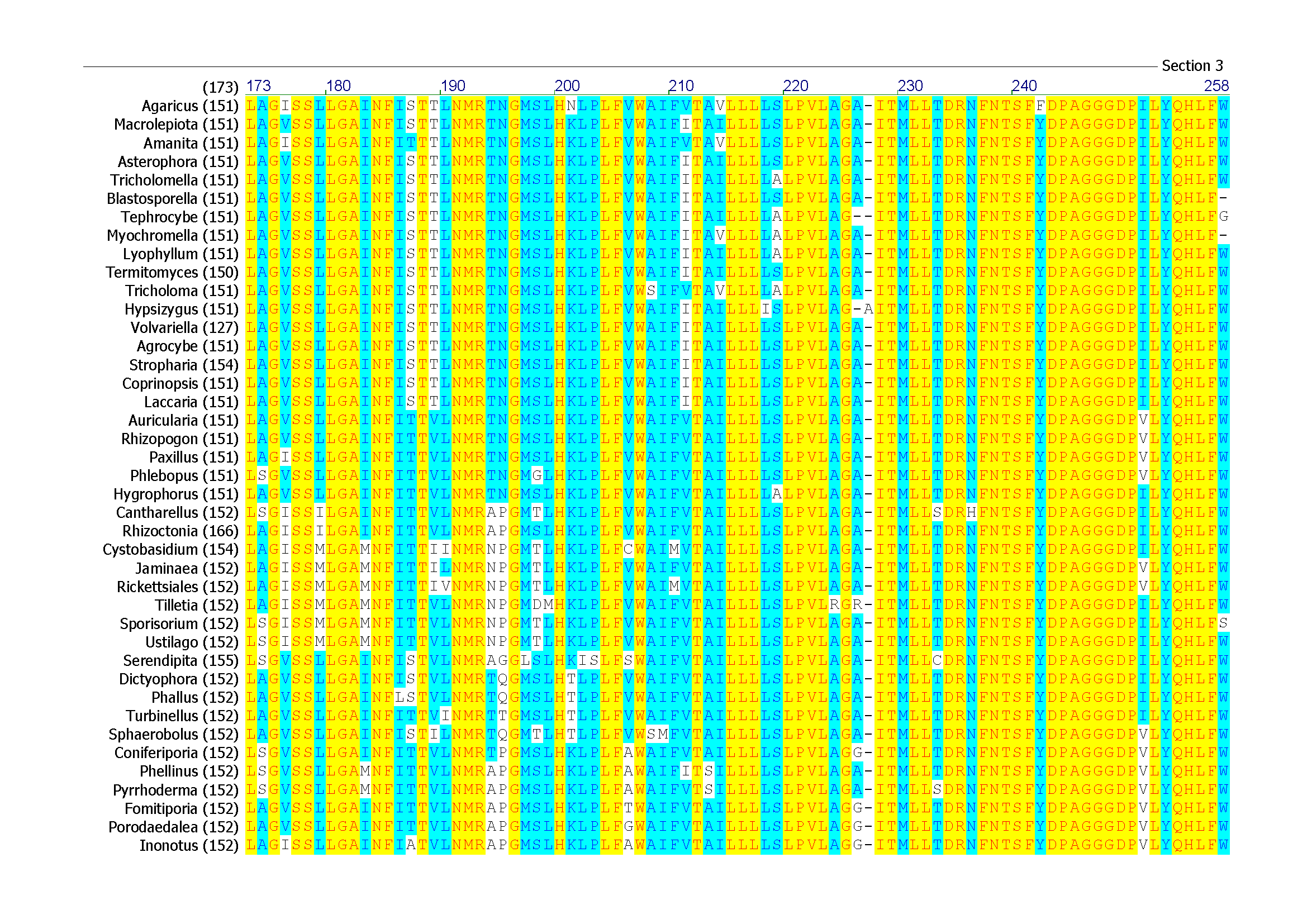
**

**
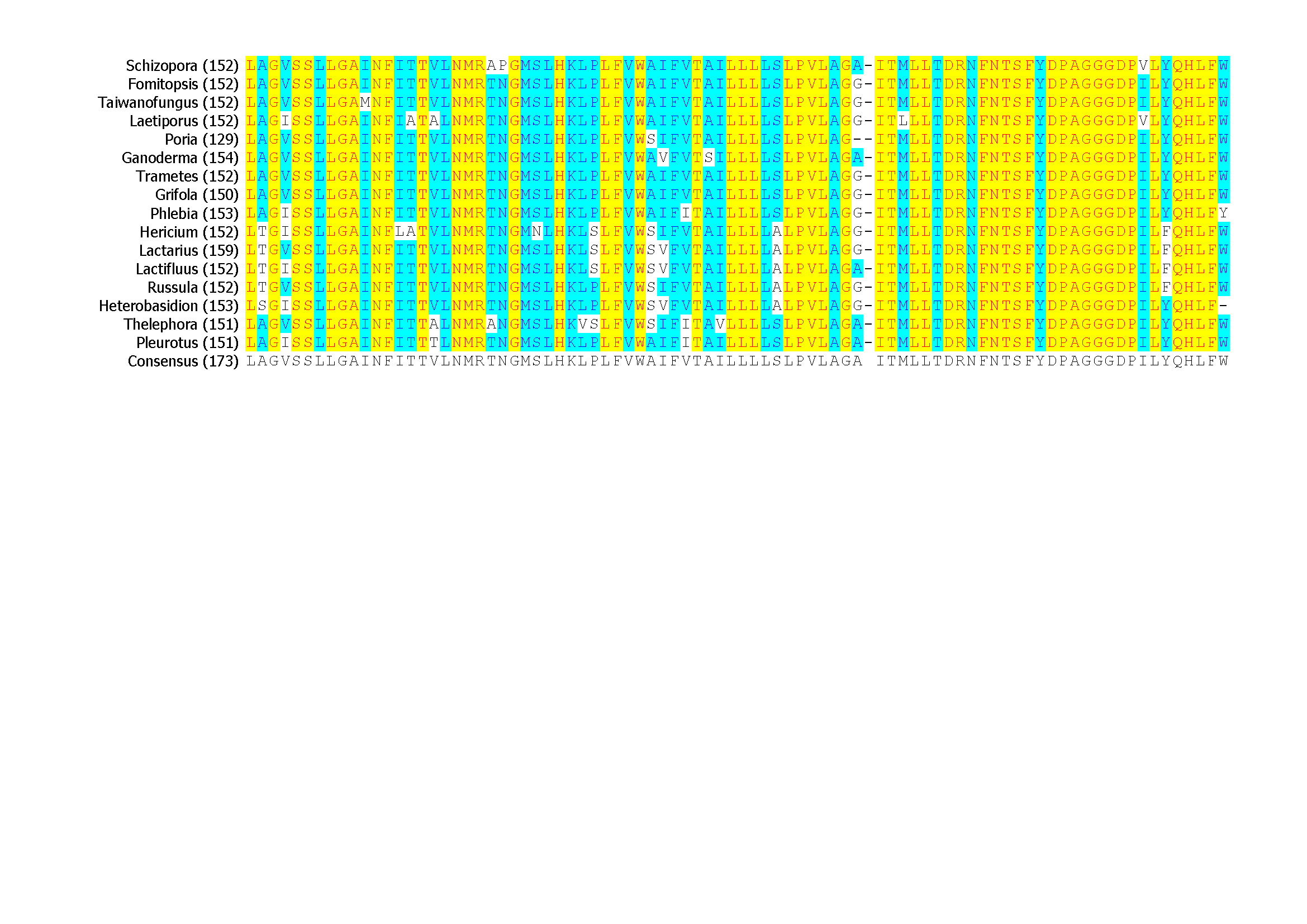
**

**
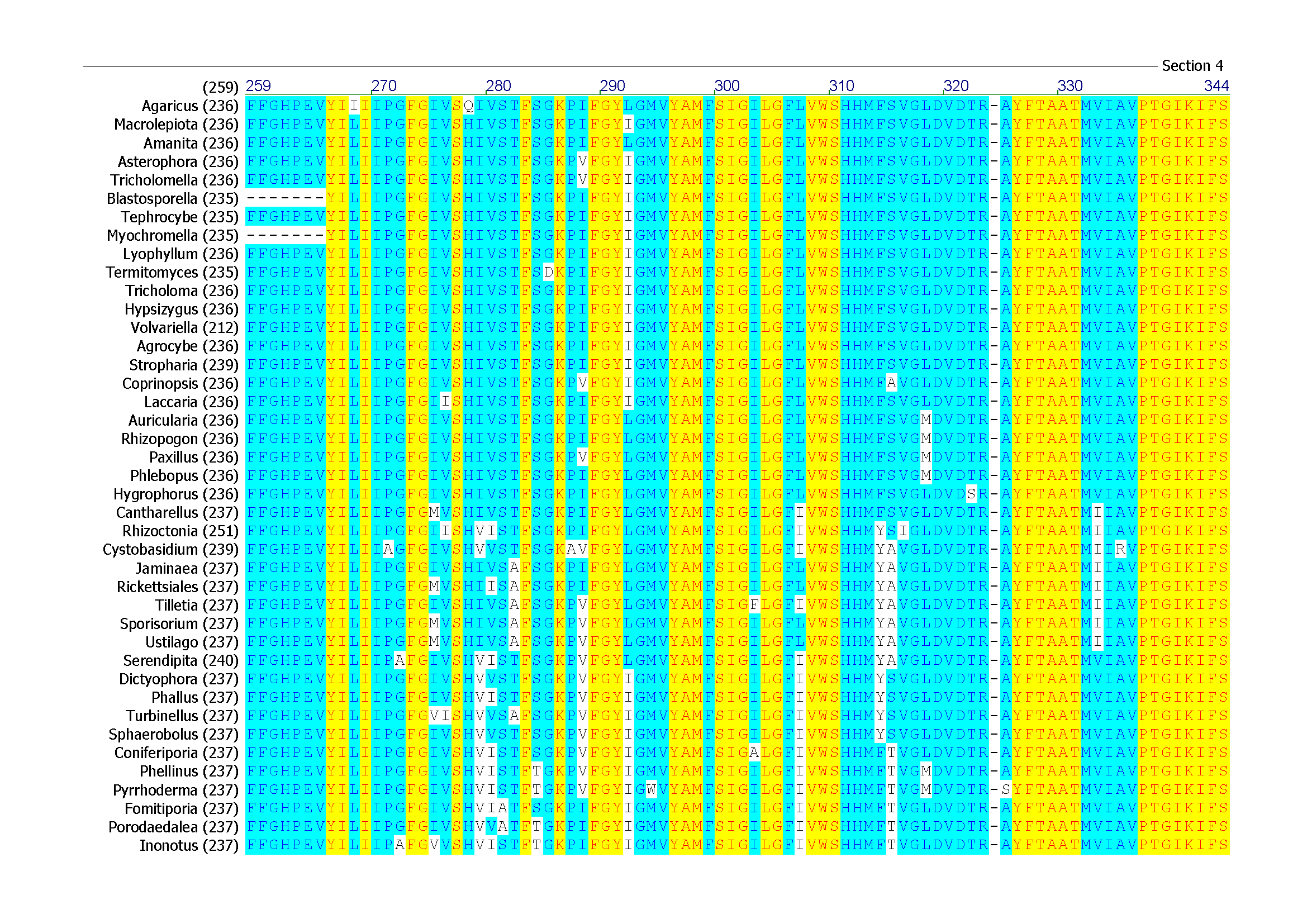
**

**
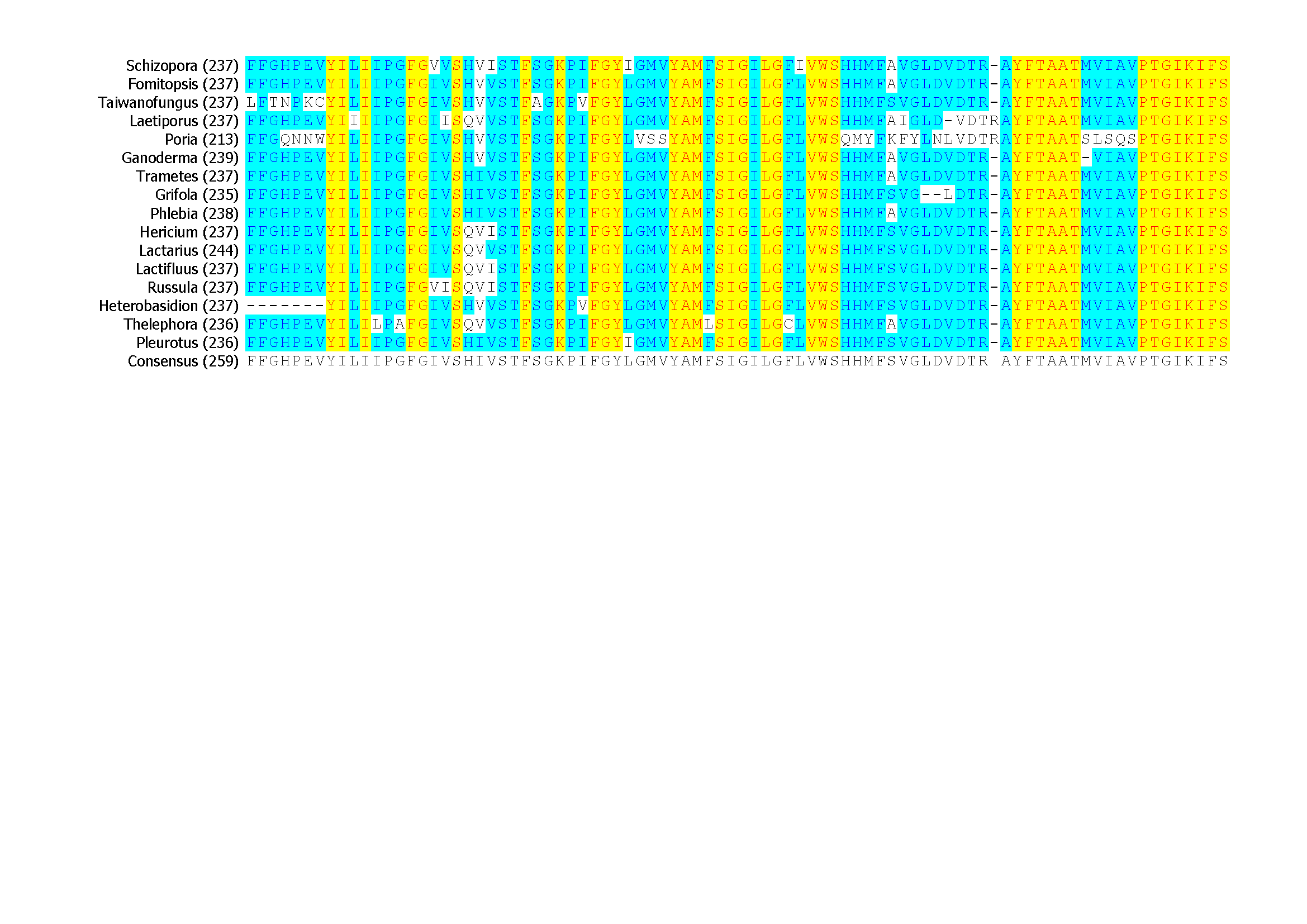

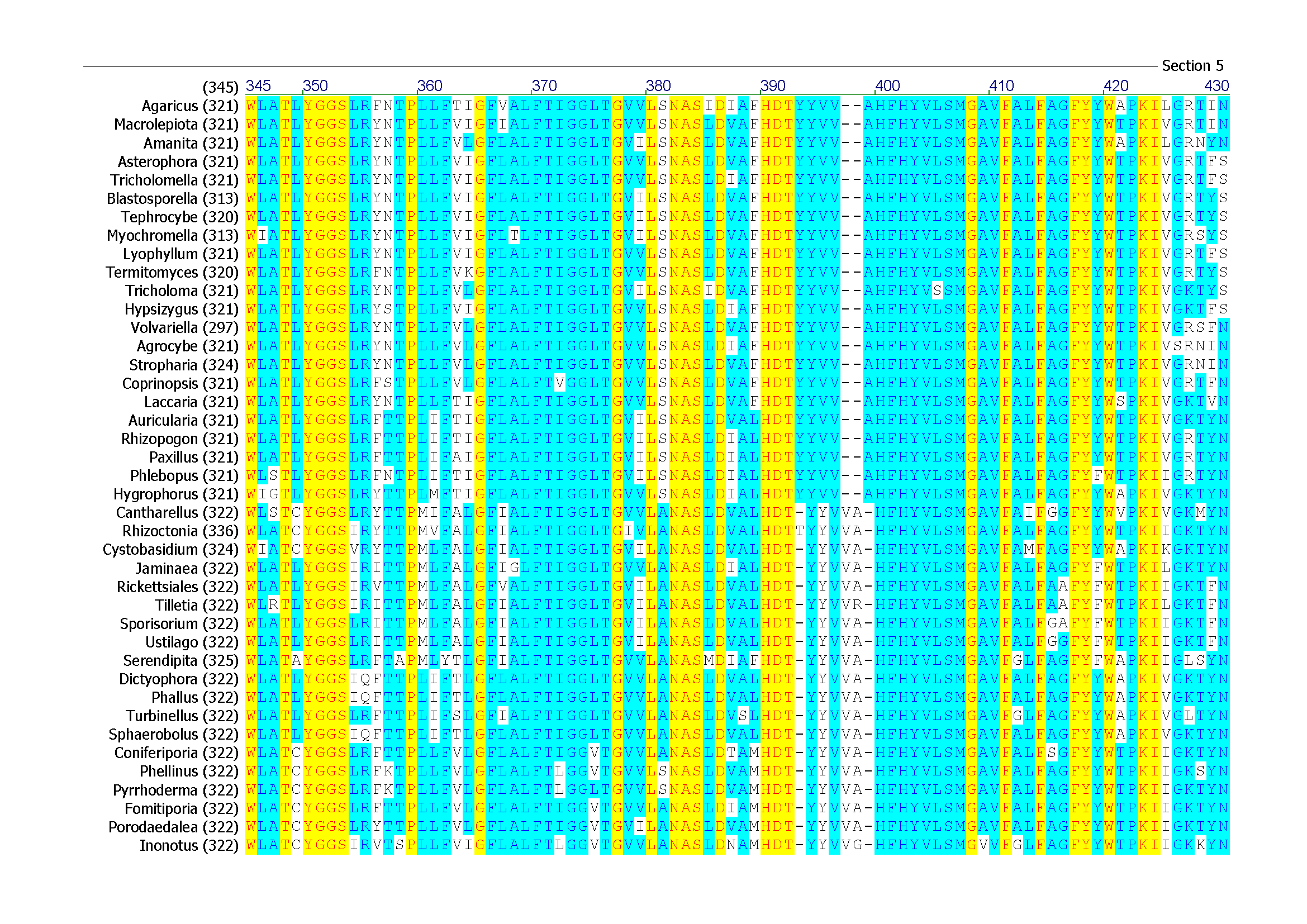
**

**
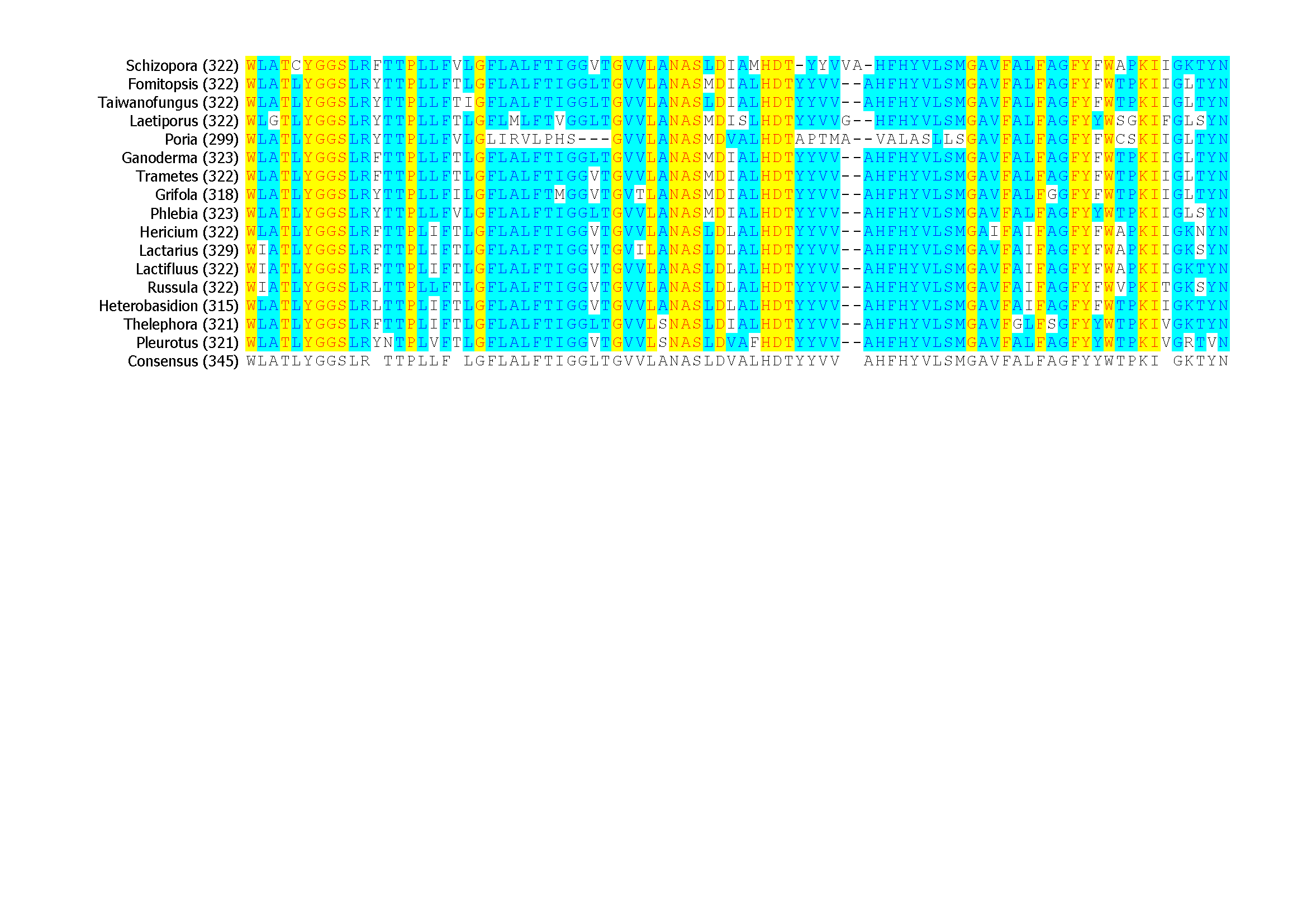
**

**
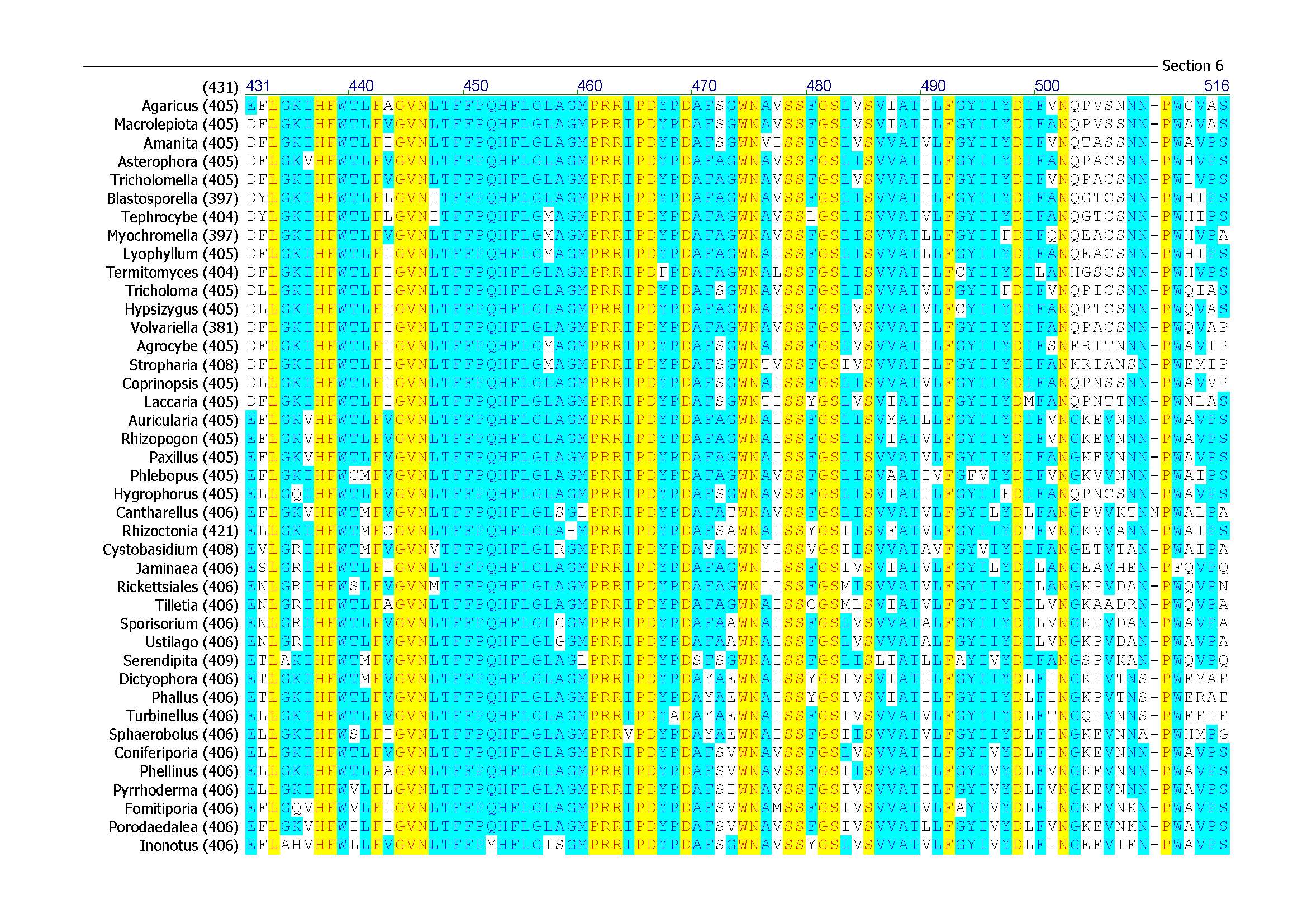
**

**
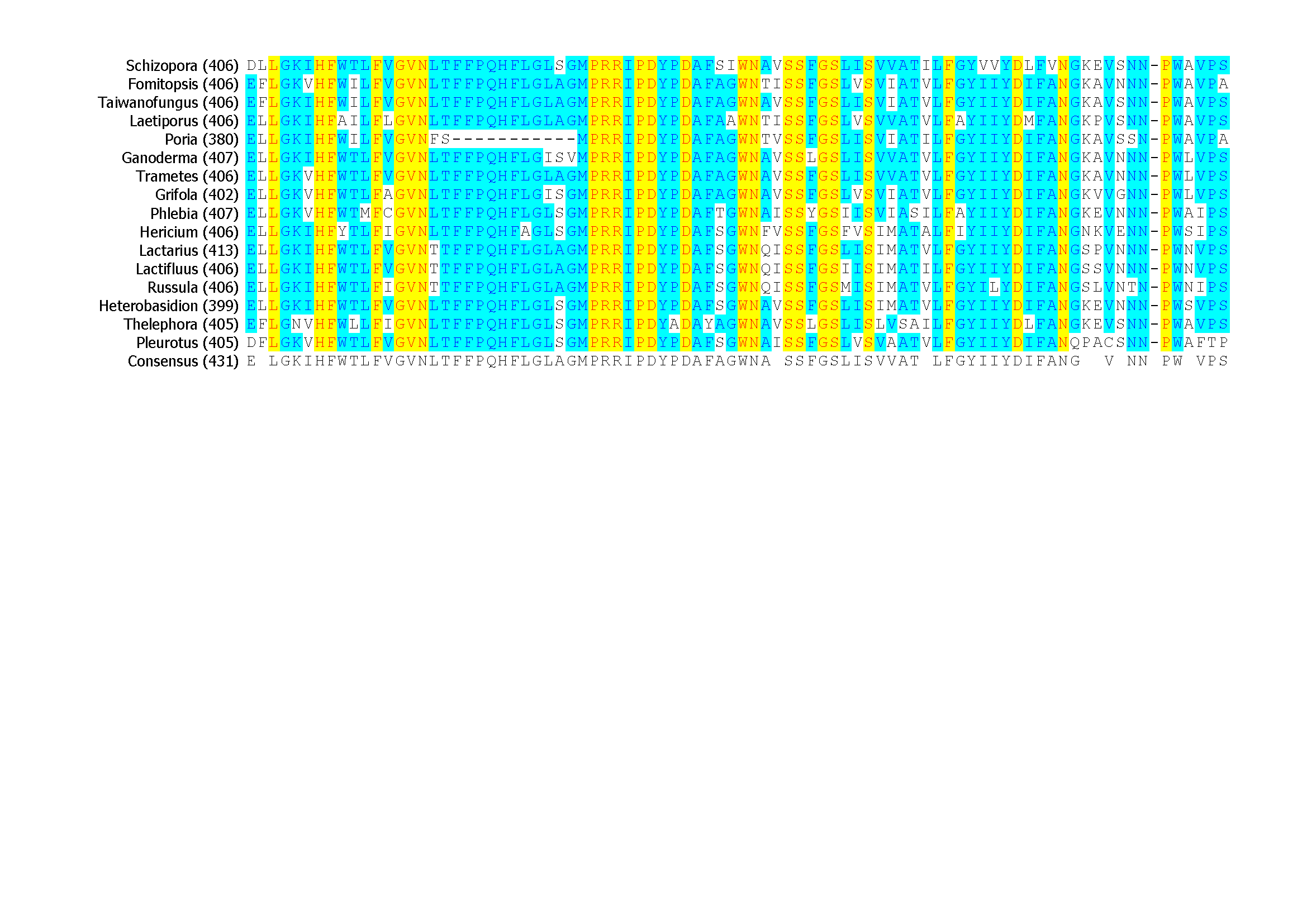
**

**
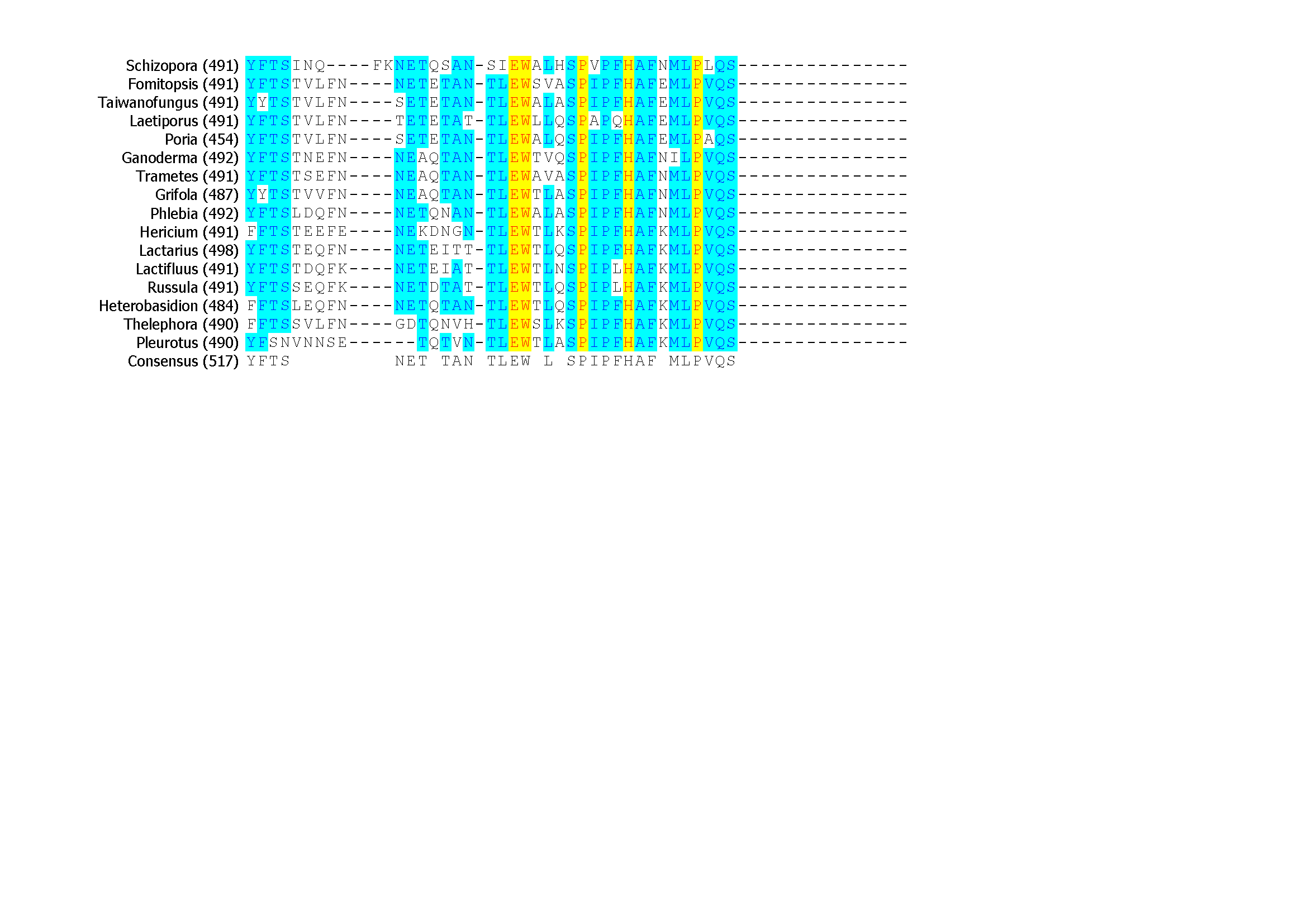

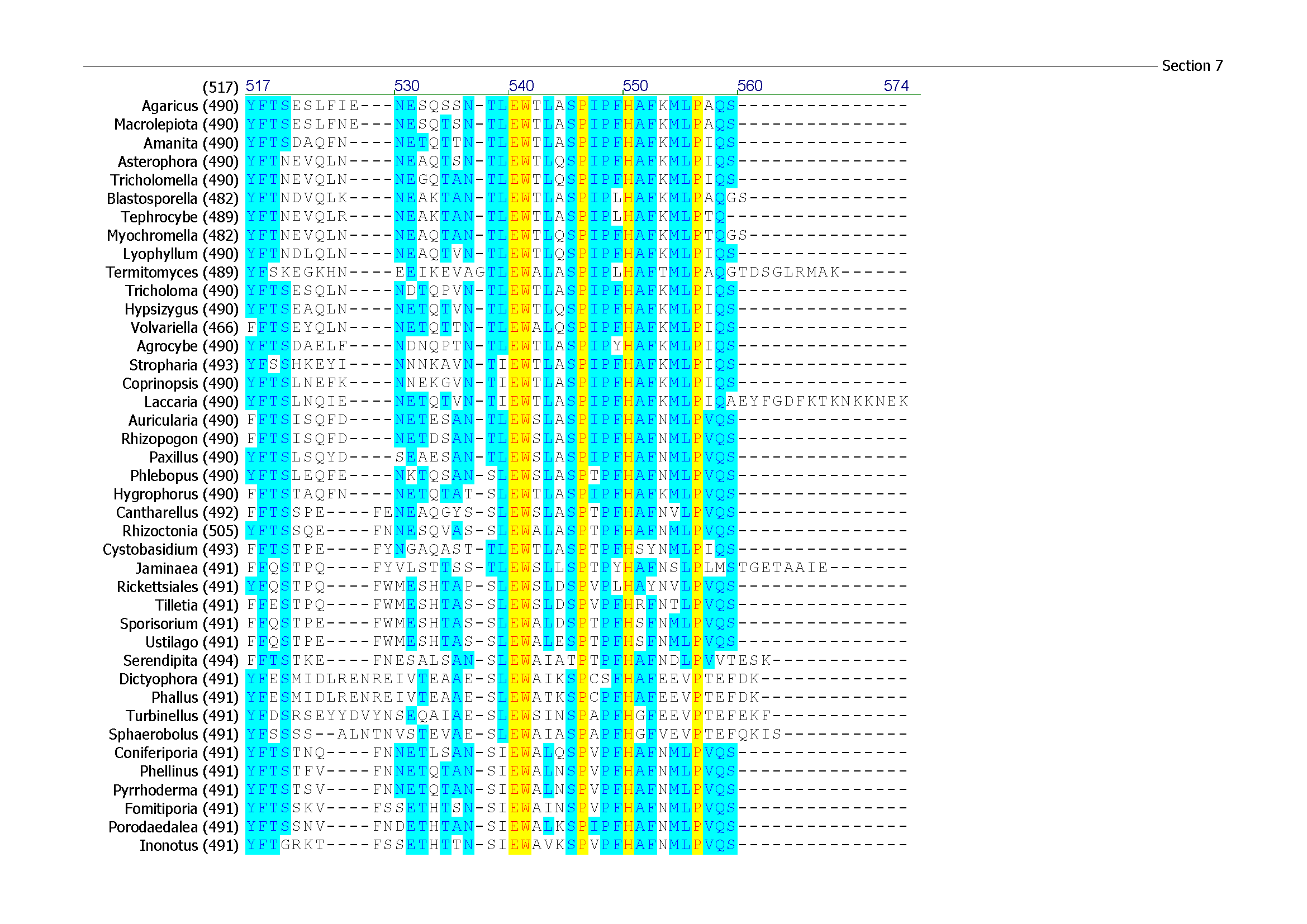
**

**Supplementary Figure S3a. Sequence of Mitochondria COX1 genes in different fungi**

**
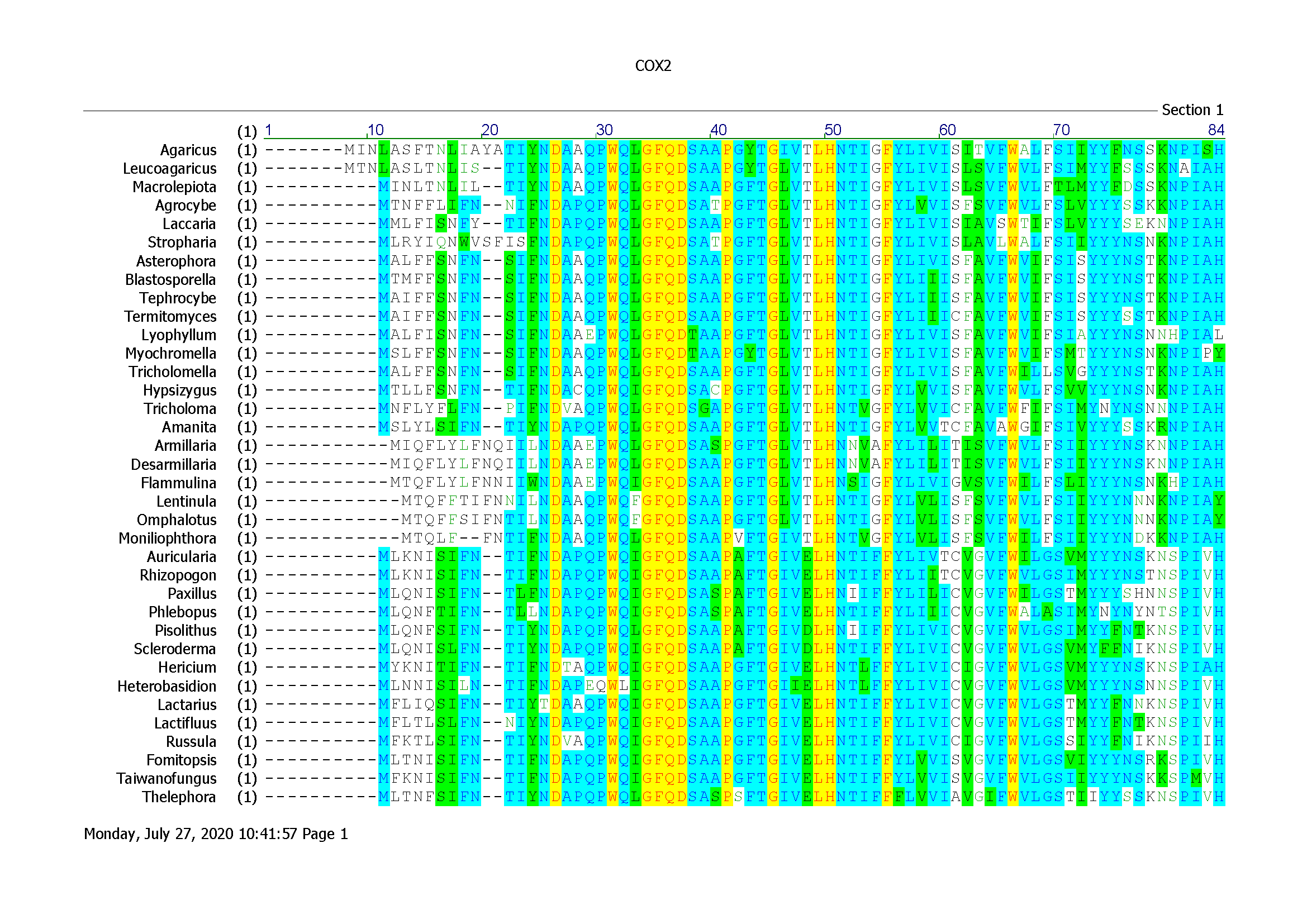
**

**
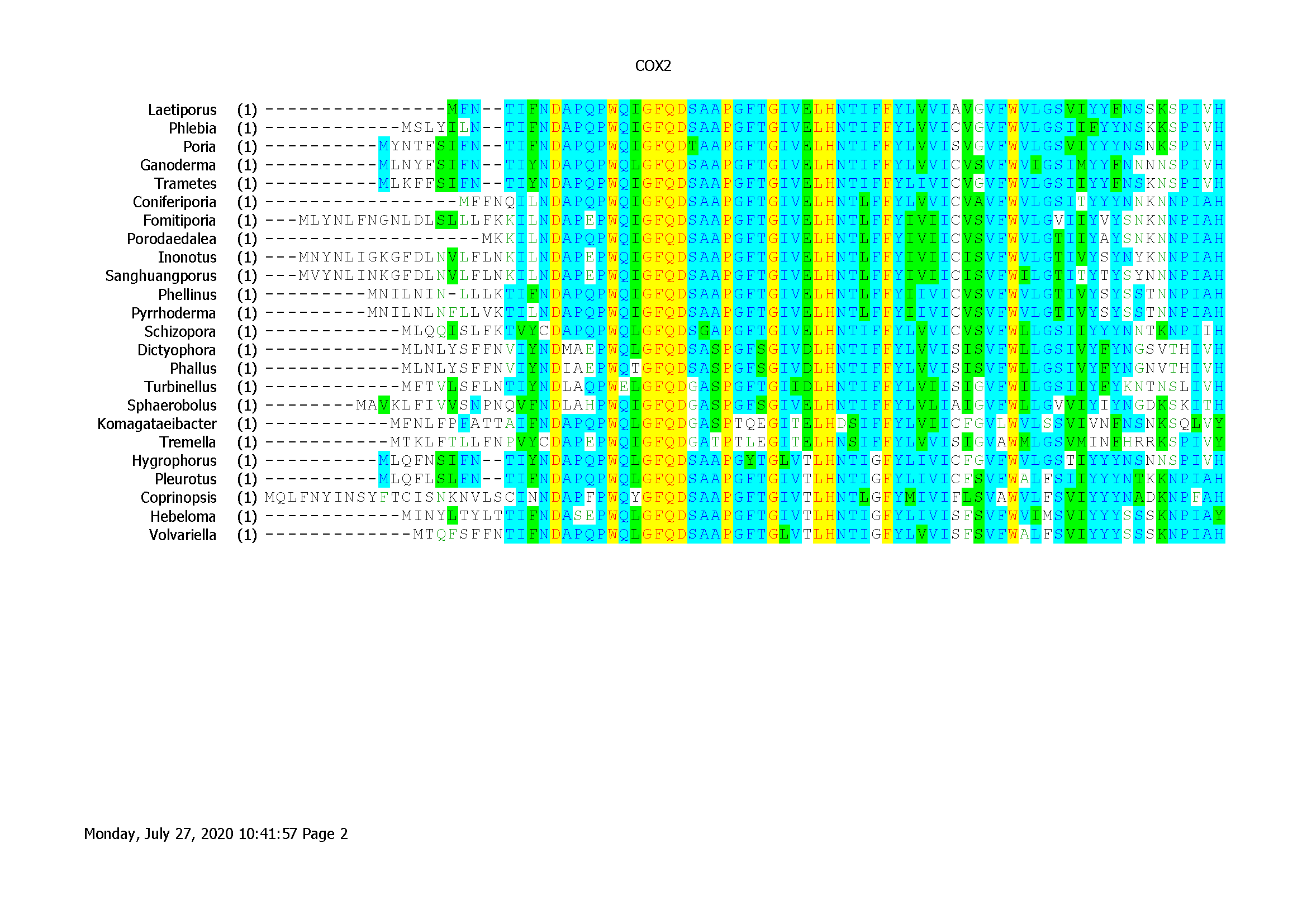
**

**
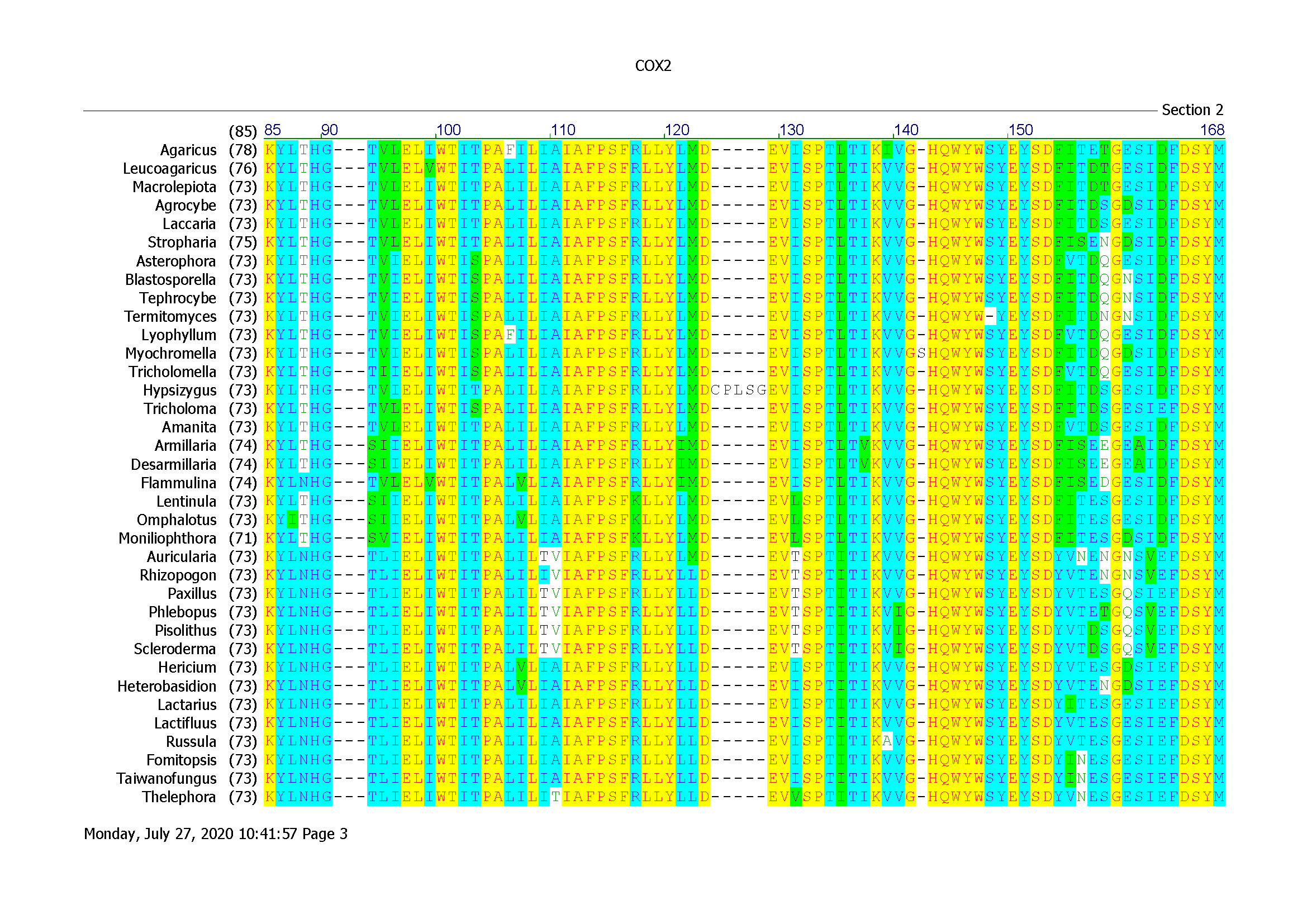

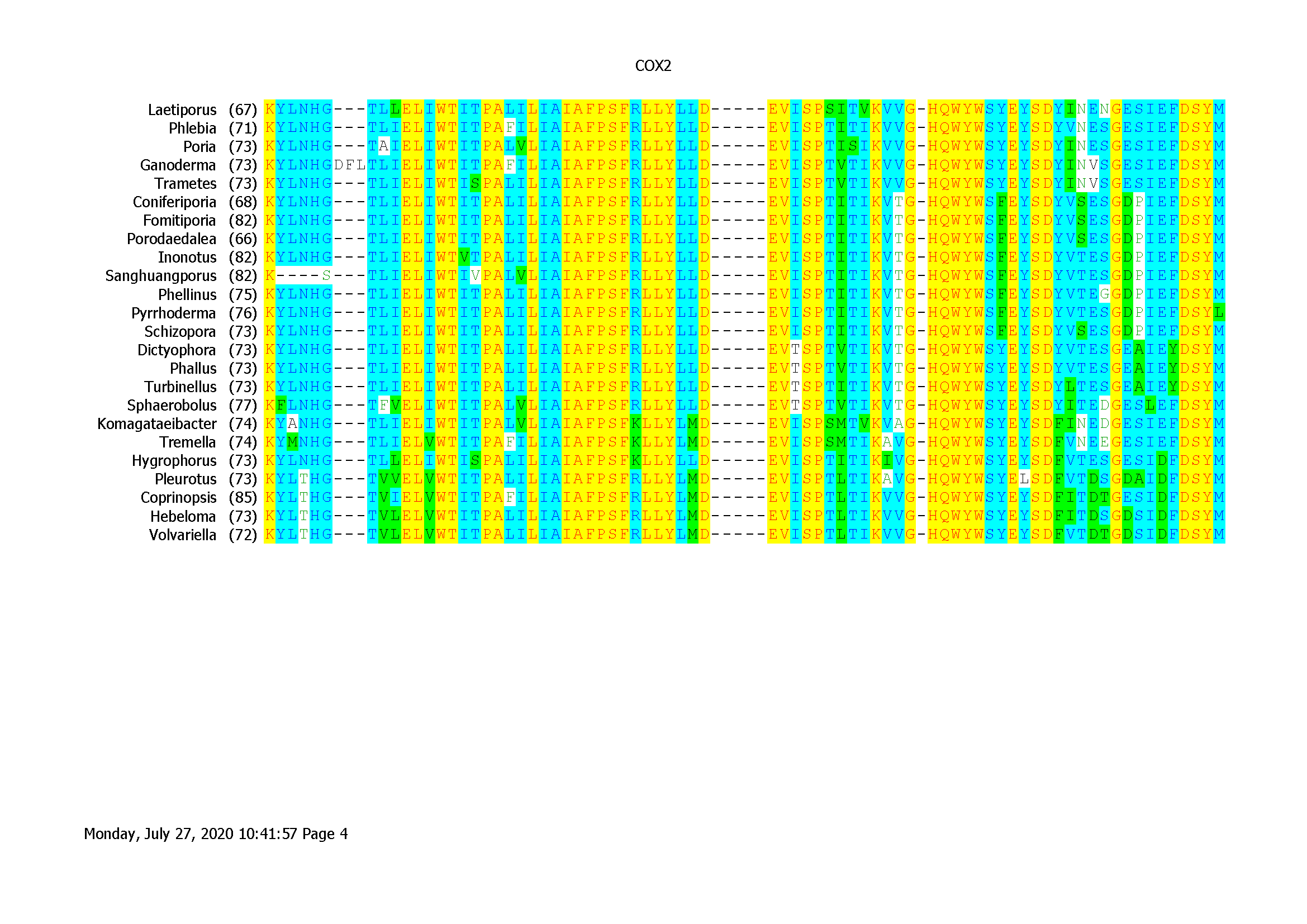
**

**
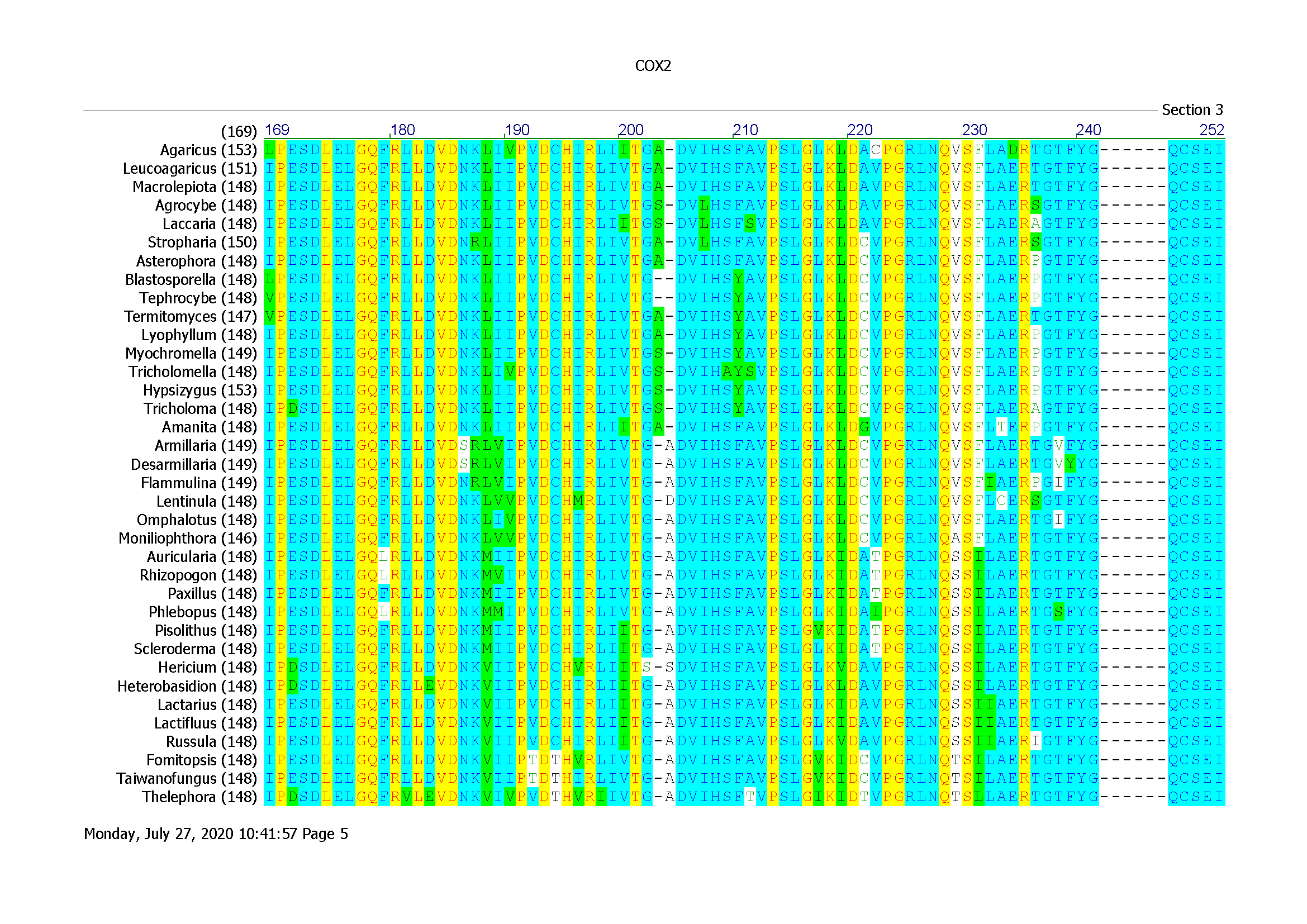
**

**
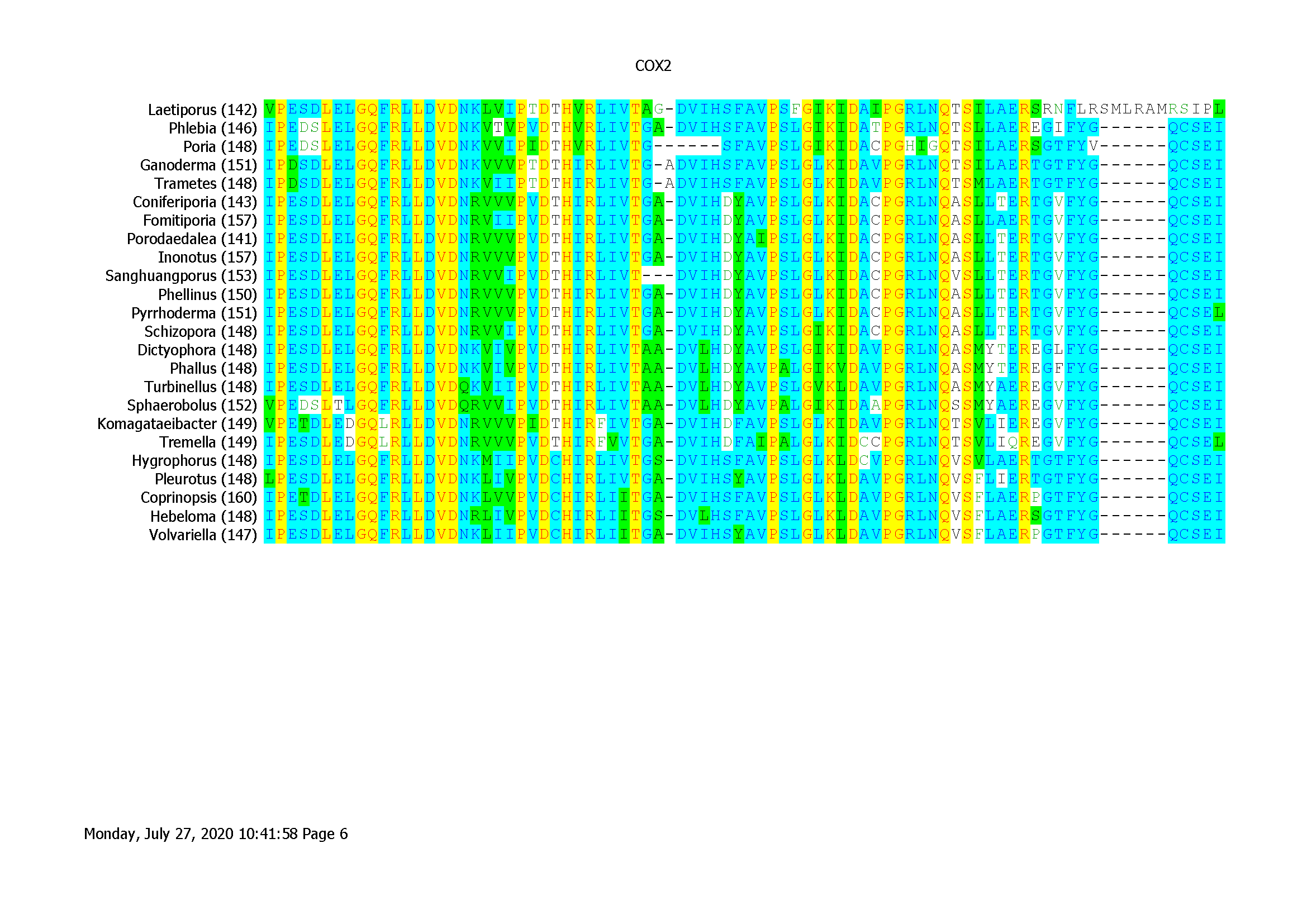
**

**
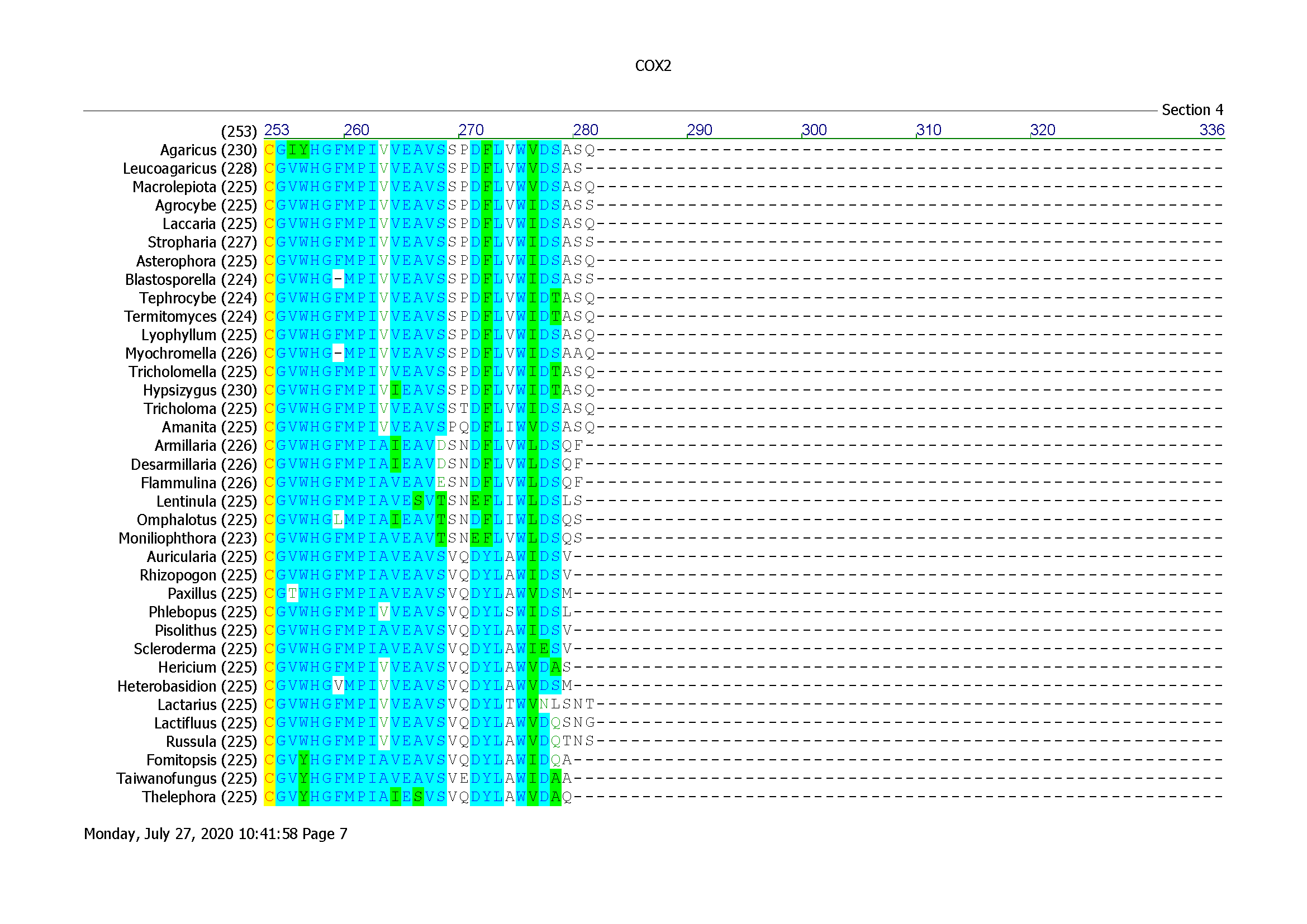
**

**
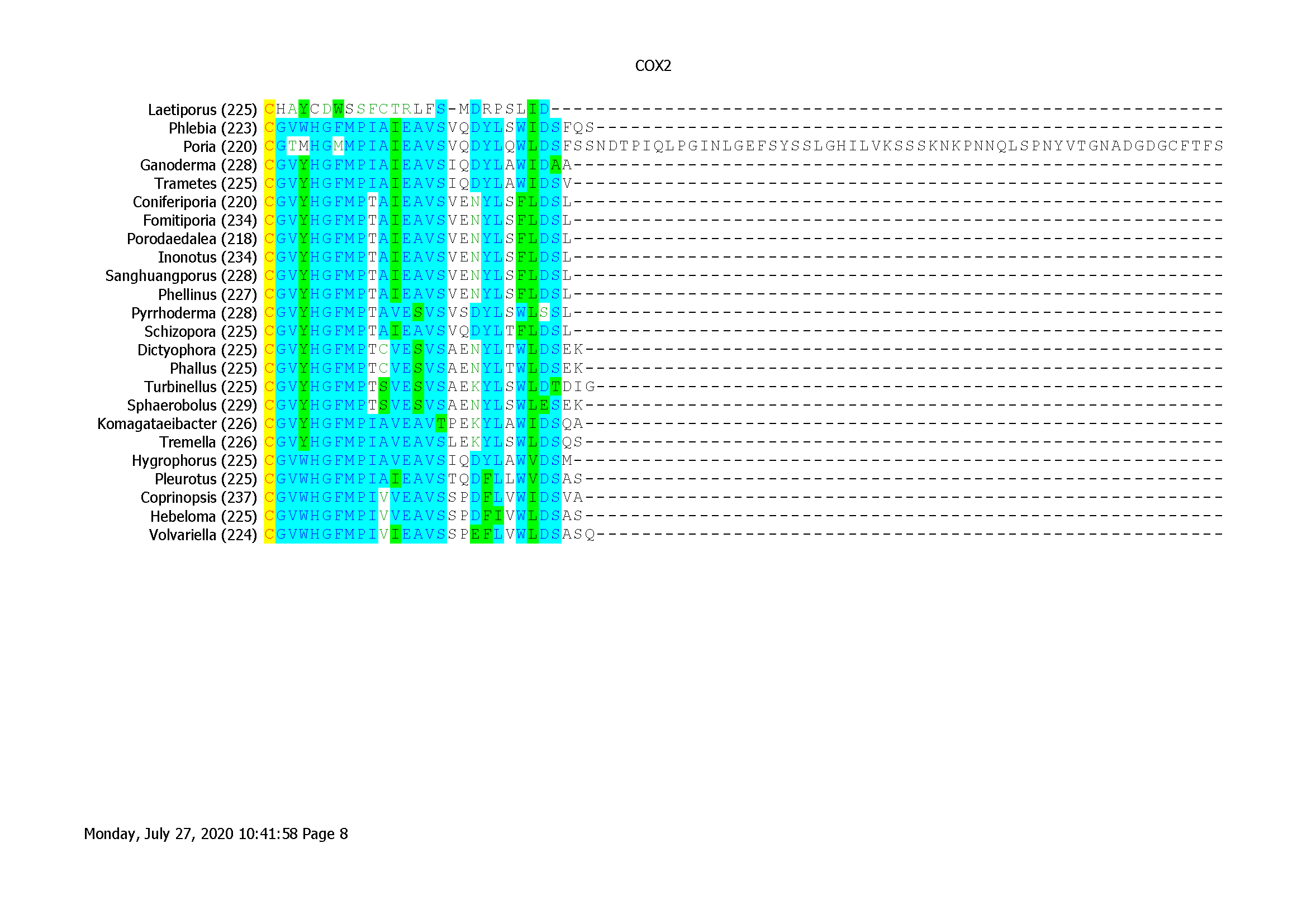
**

**
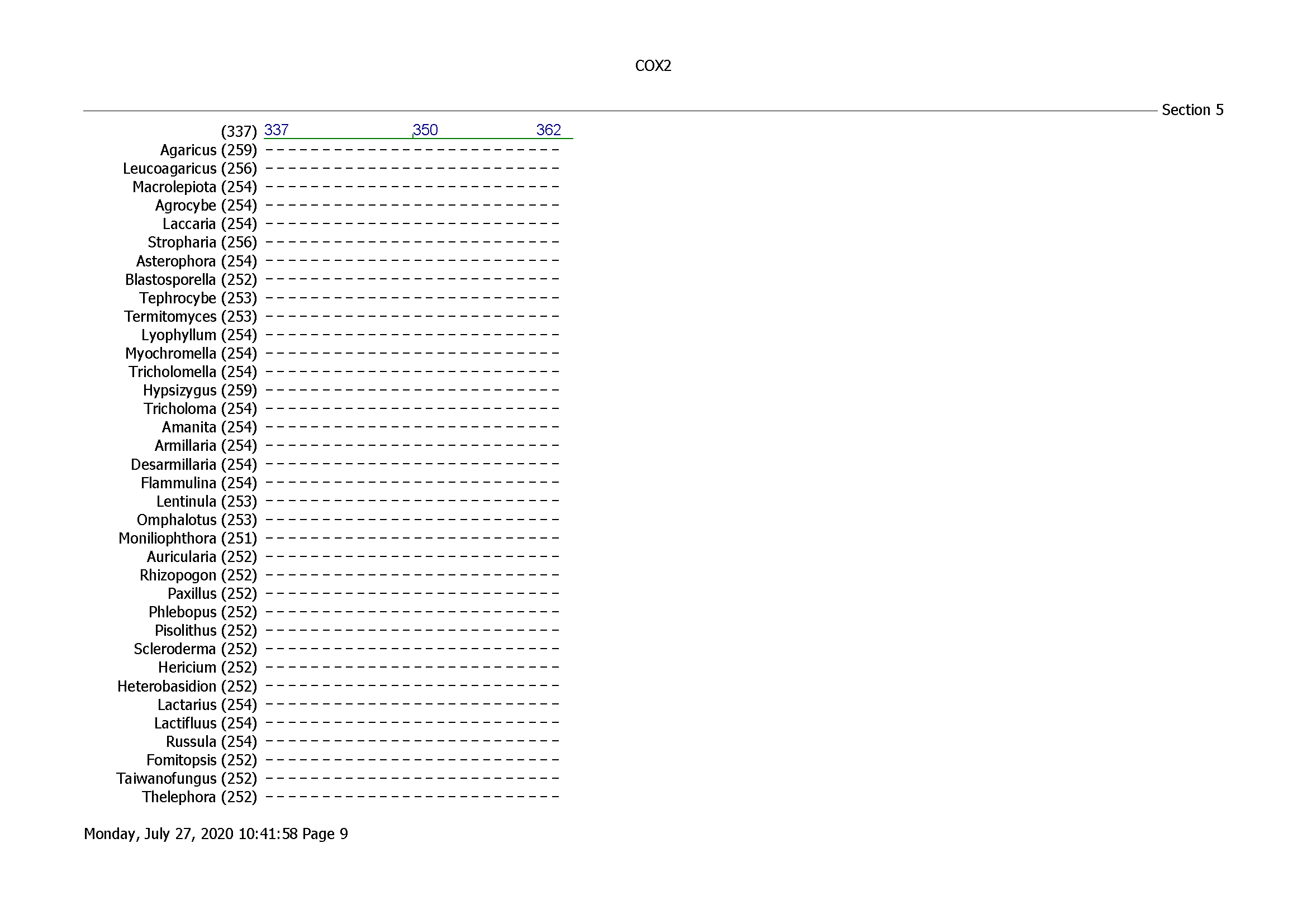
**

**

**

**Supplementary Figure S3b. Sequence of Mitochondria COX2 genes in different fungi**

**
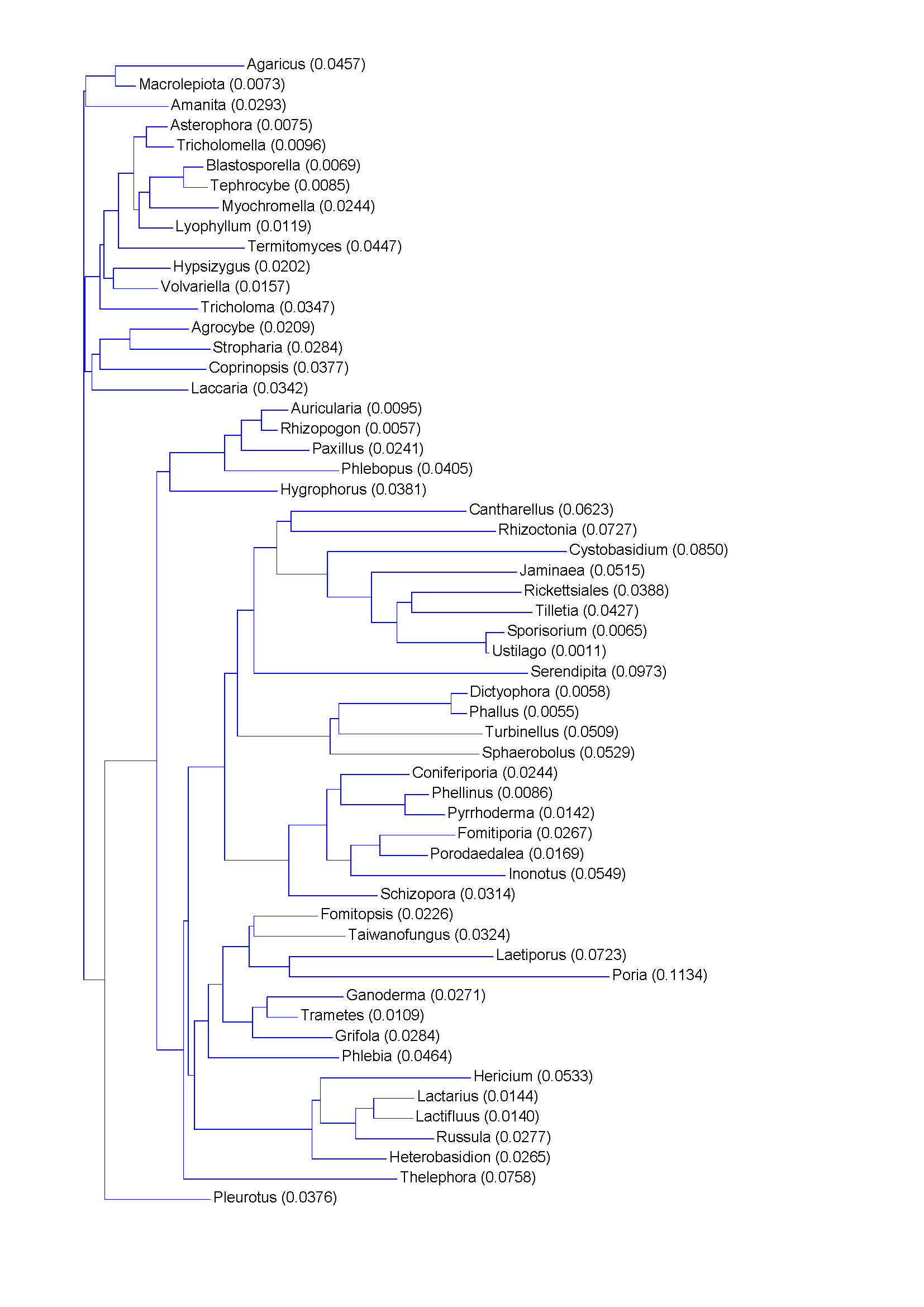
**

**Supplementary Figure S3c. Phylogenic tree of Mitochondria COX1 genes from different fungi**

**
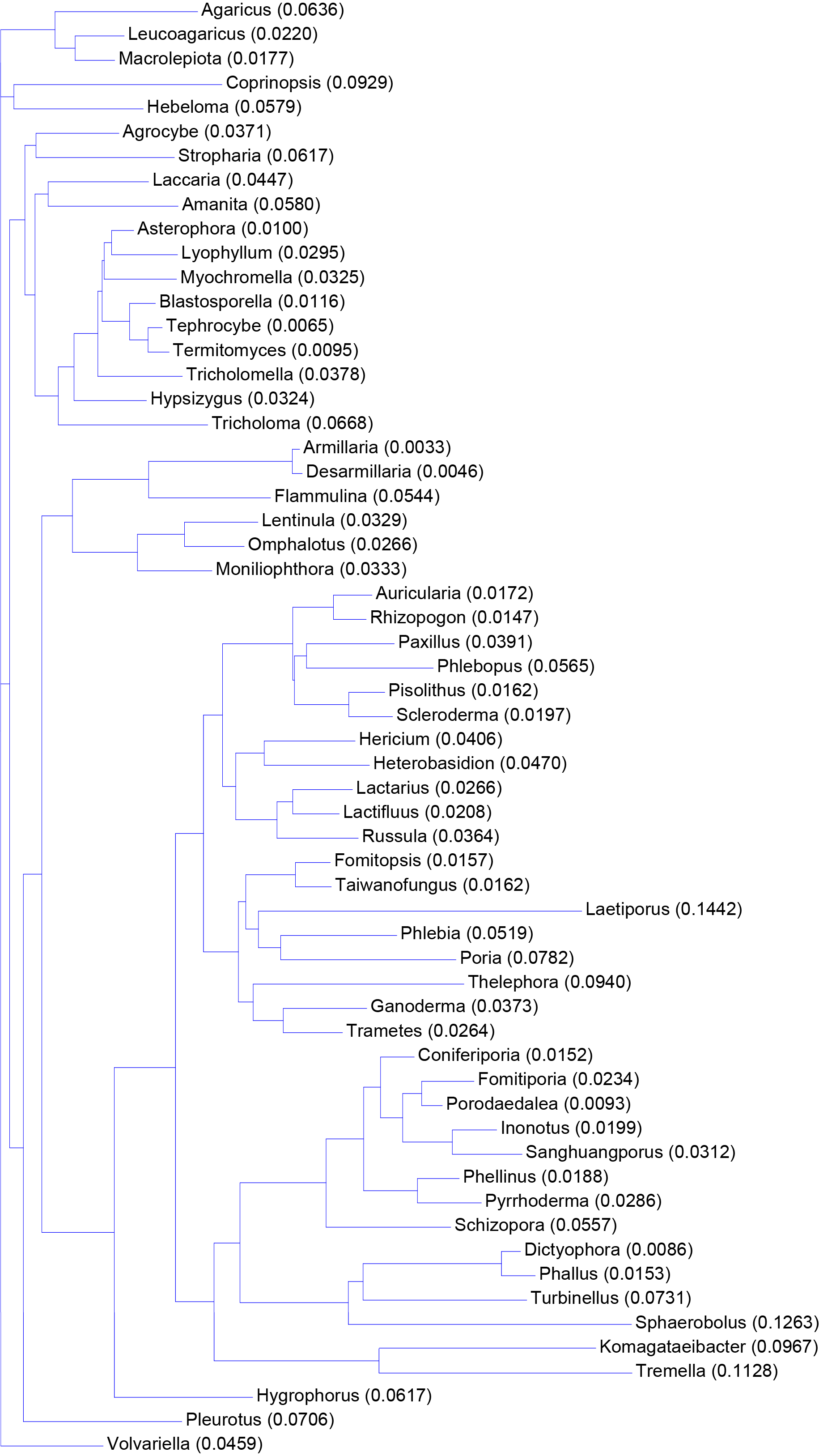
**

**Supplementary Figure S3d. Phylogenic tree of Mitochondria COX2 genes from different fungi**


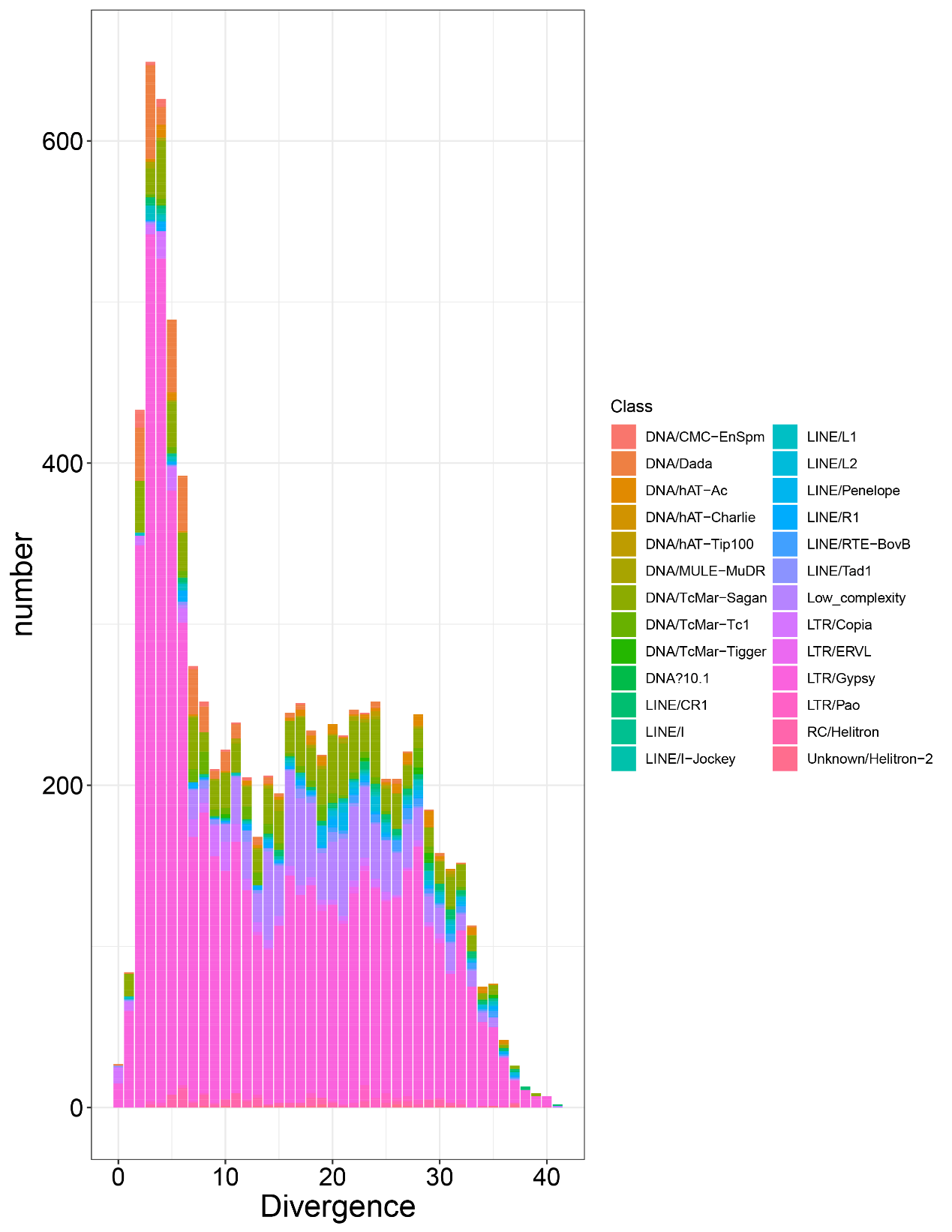


**Supplementary Figure S4. *W. cocos* genome transposable elements (TE) landscape map.** Repeat sequences are predicted and masked by RepeatMasker and RepeatModeler.


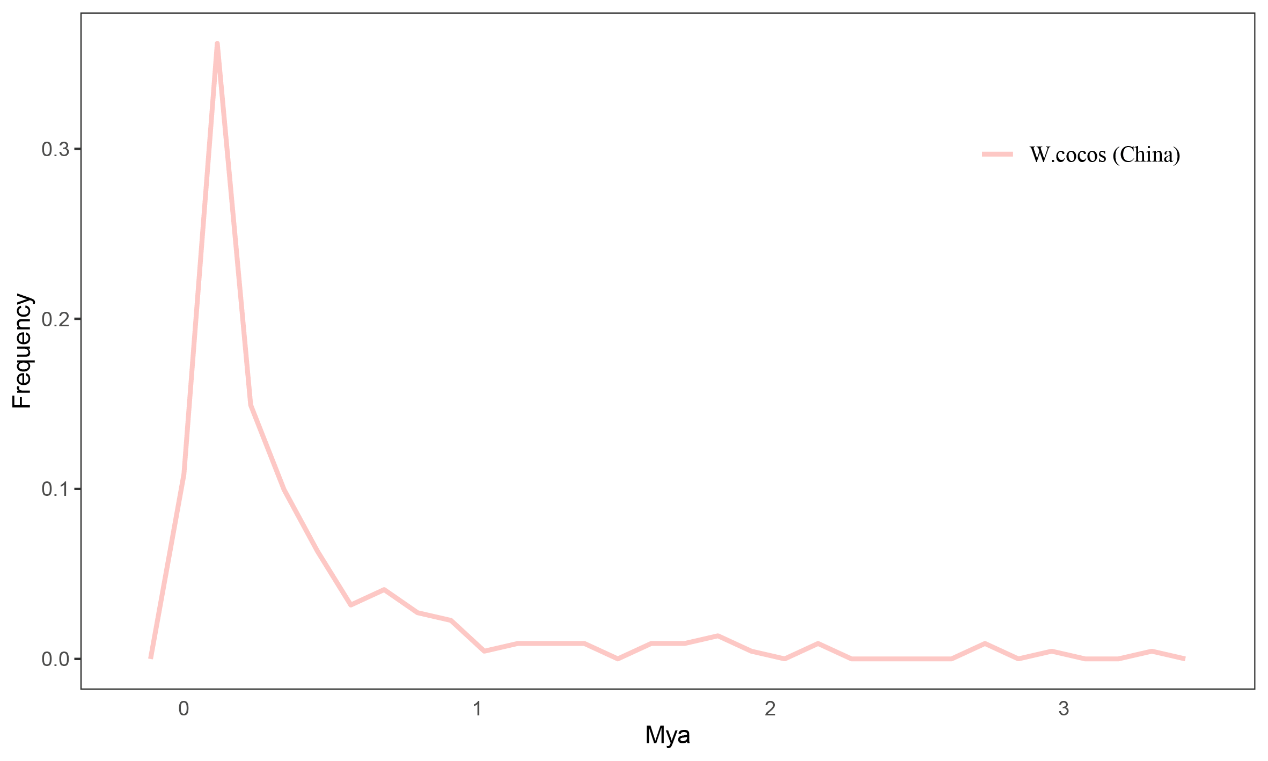


**Supplementary Figure S5. The estimated insertion time of LTR (long terminal repeat) in *W. cocos* genome.** LTR sequence was predicted by LTR-finder, and then LTR_retriever software was used to estimate the LTR insertion time.


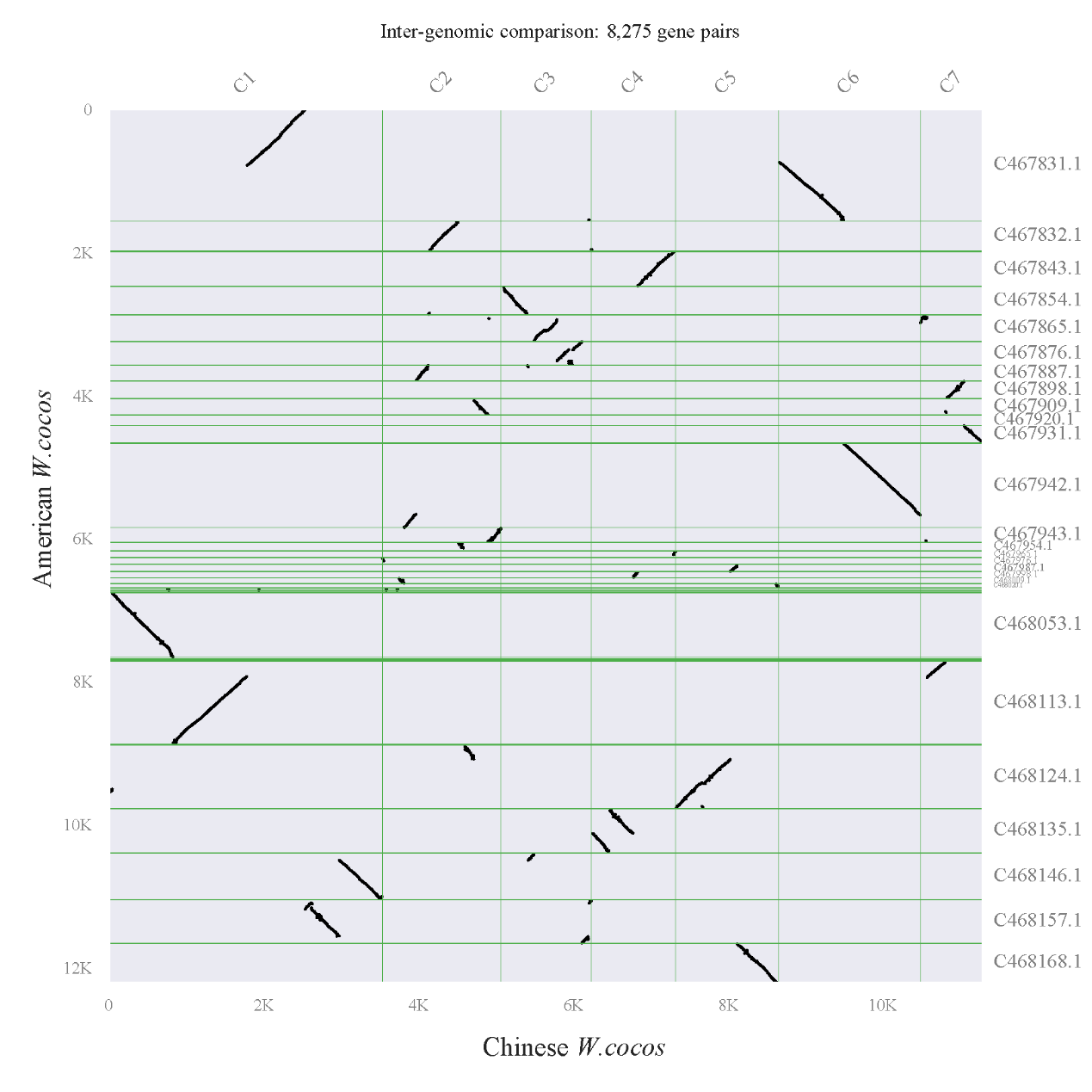


**Supplementary Figure S6. Syntenic dot plots show chromosomal relationship between Chinese and American *W. cocos* genome.**


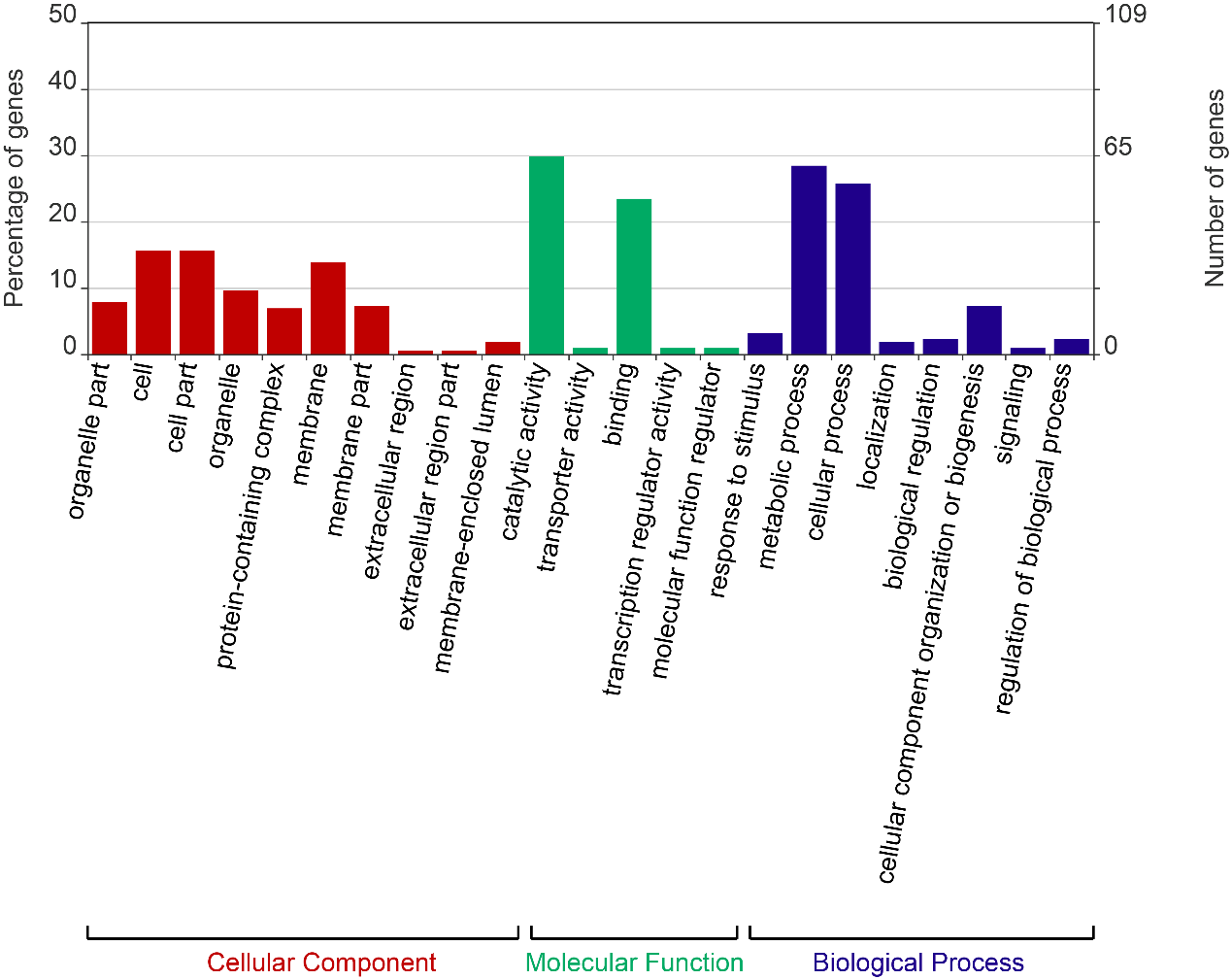


**Supplementary Figure S7. The GO function annotations of a block containing 242 gens only exit in Chinese *W. cocos*.**


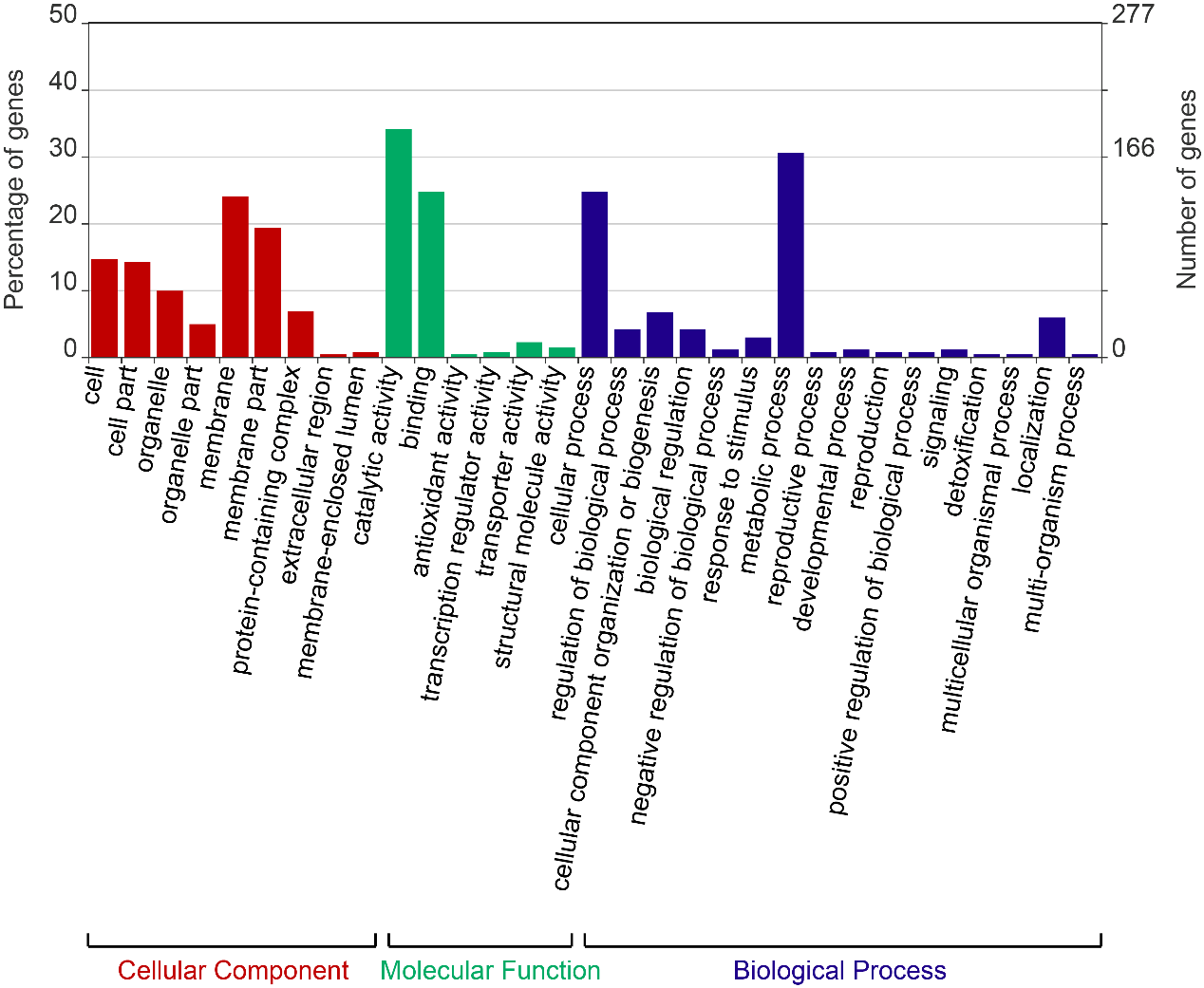


**Supplementary Figure S8.** **The GO function annotations of 555 genes participated in WGD events of *W. cocos*.**

**
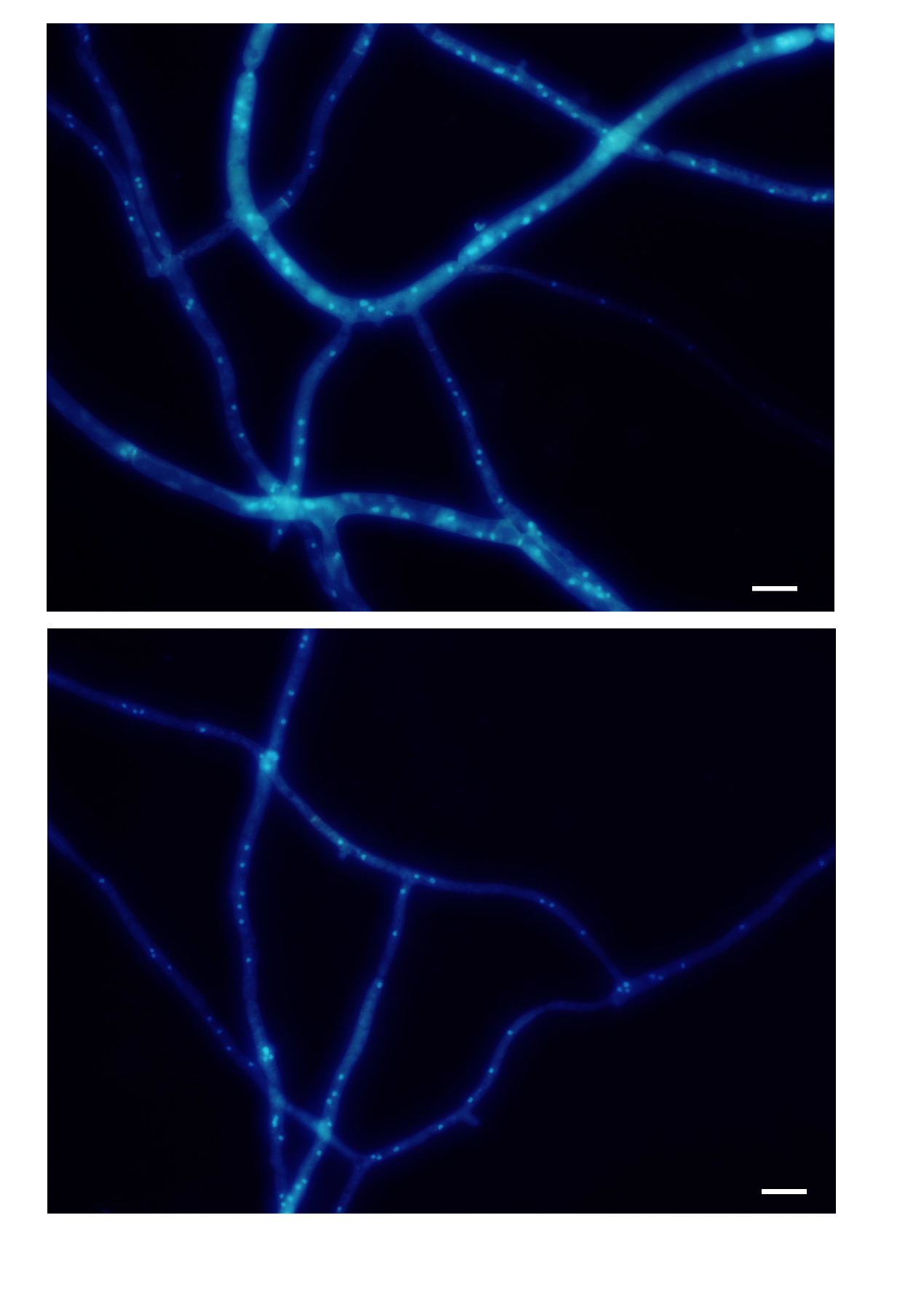
**

**Supplementary Figure S9. Observation of cultured mycelia by fluorescence microscope.** Upper: wild type mycelia. Below: screened mycelia grew from protoplasm with lesser polynuclear cells. Nuclei are stained by 50 μg/ml Hoechst 33258 solution. Bar, 20 μm


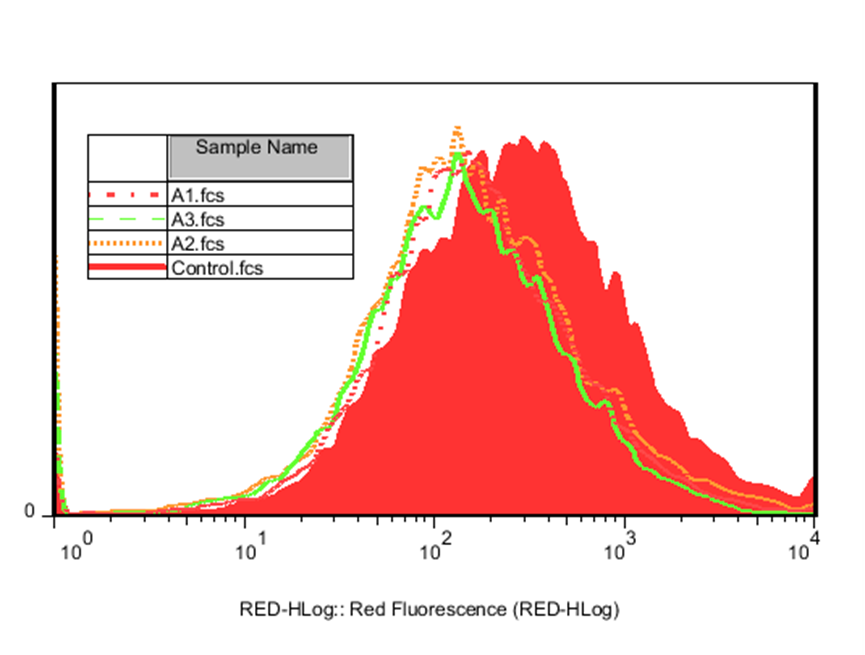


**Supplementary Figure S10.** **Flow cytometry analysis of the fluorescence expression of lesser nuclear cells and wild *W. cocos* strains.** A1,A2,A3 are three biological replicates of lesser nuclear strain. Control is wild strain. The main peak position of lesser nuclear strain fluorescence detected by flow cytometry is about 144, and the peak of wild strain is about 260. The average fluorescence expression of single cells is close to 1:2.

**
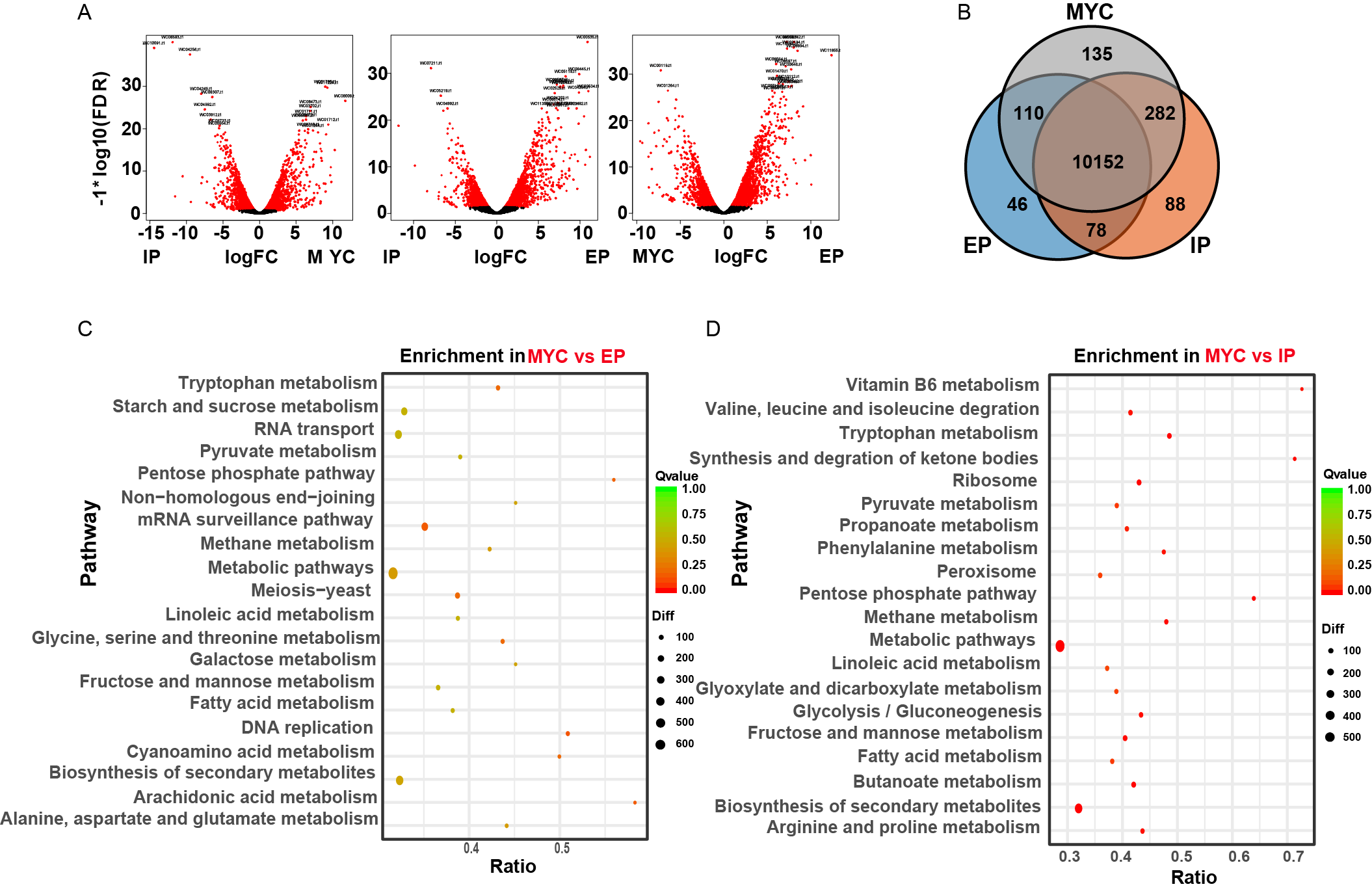
**

**Supplementary Figure S11. Analysis of gene expression in different tissues of WCLT.** A, Volcano plots of differentially expressed genes in mycelia (MYC), inner part (IP) and epidermis of sclerotium (EP). B, A Venn diagram of differentially expressed genes in MYC, IP and EP. C, Pathway Enrichment of differentially expressed genes for MYC-vs-EP. D, Pathway Enrichment of differentially expressed genes for MYC-vs-IP.


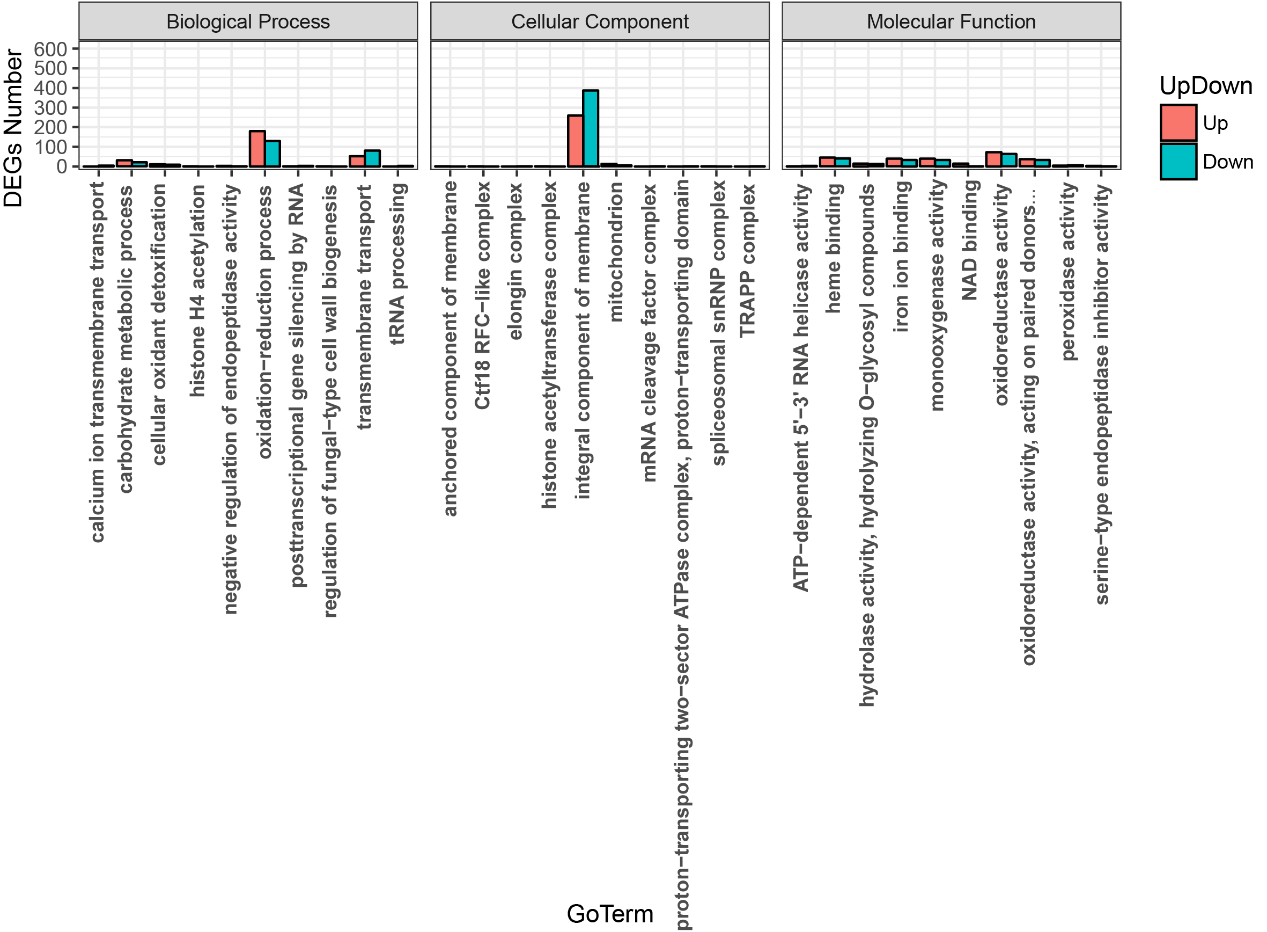


**Supplementary Figure S12a. DEGs (****comparison between MYC and IP) number of the most enriched GO Term**


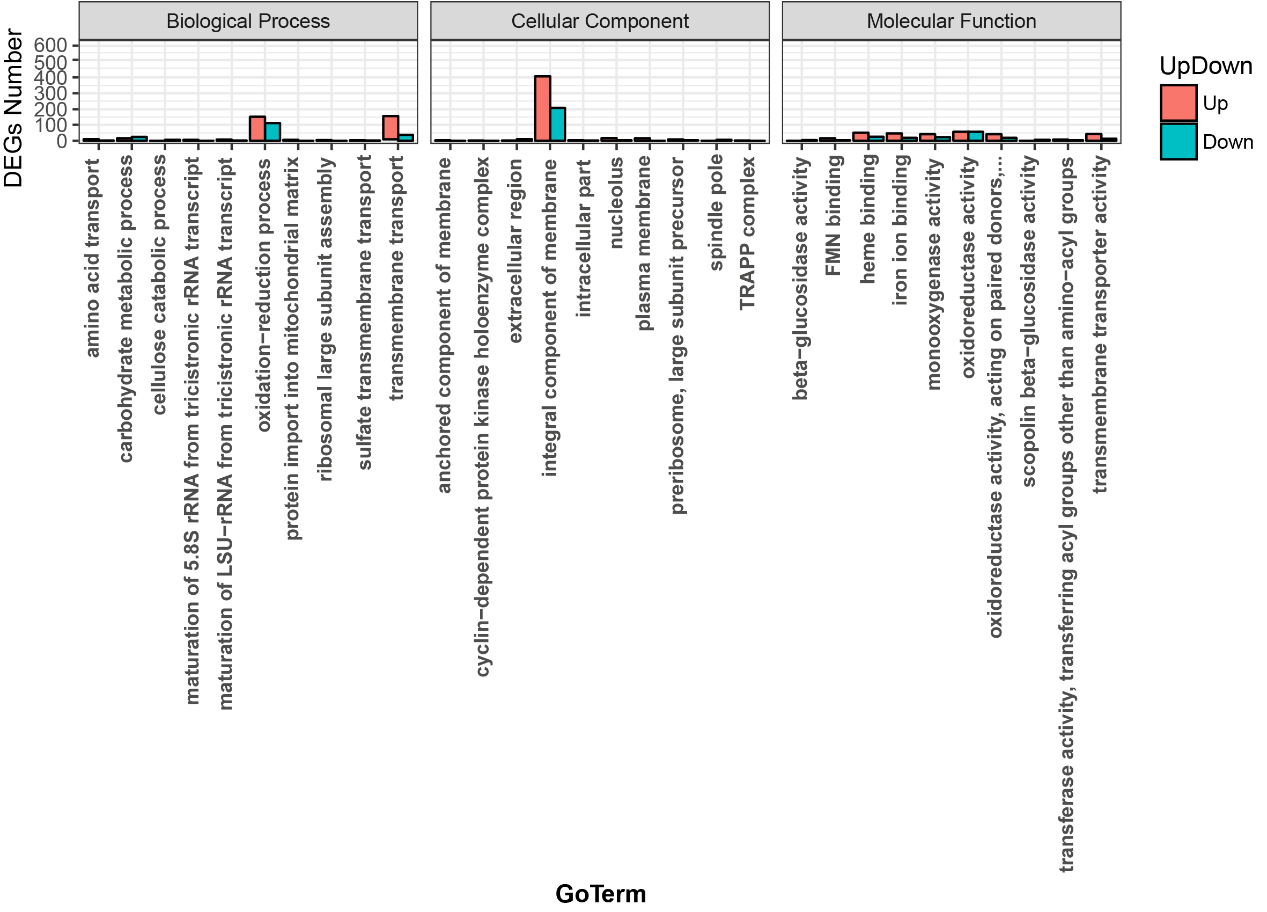


**Supplementary Figure S12b. DEGs (comparison between EP and IP) number of the most enriched GO Term.**


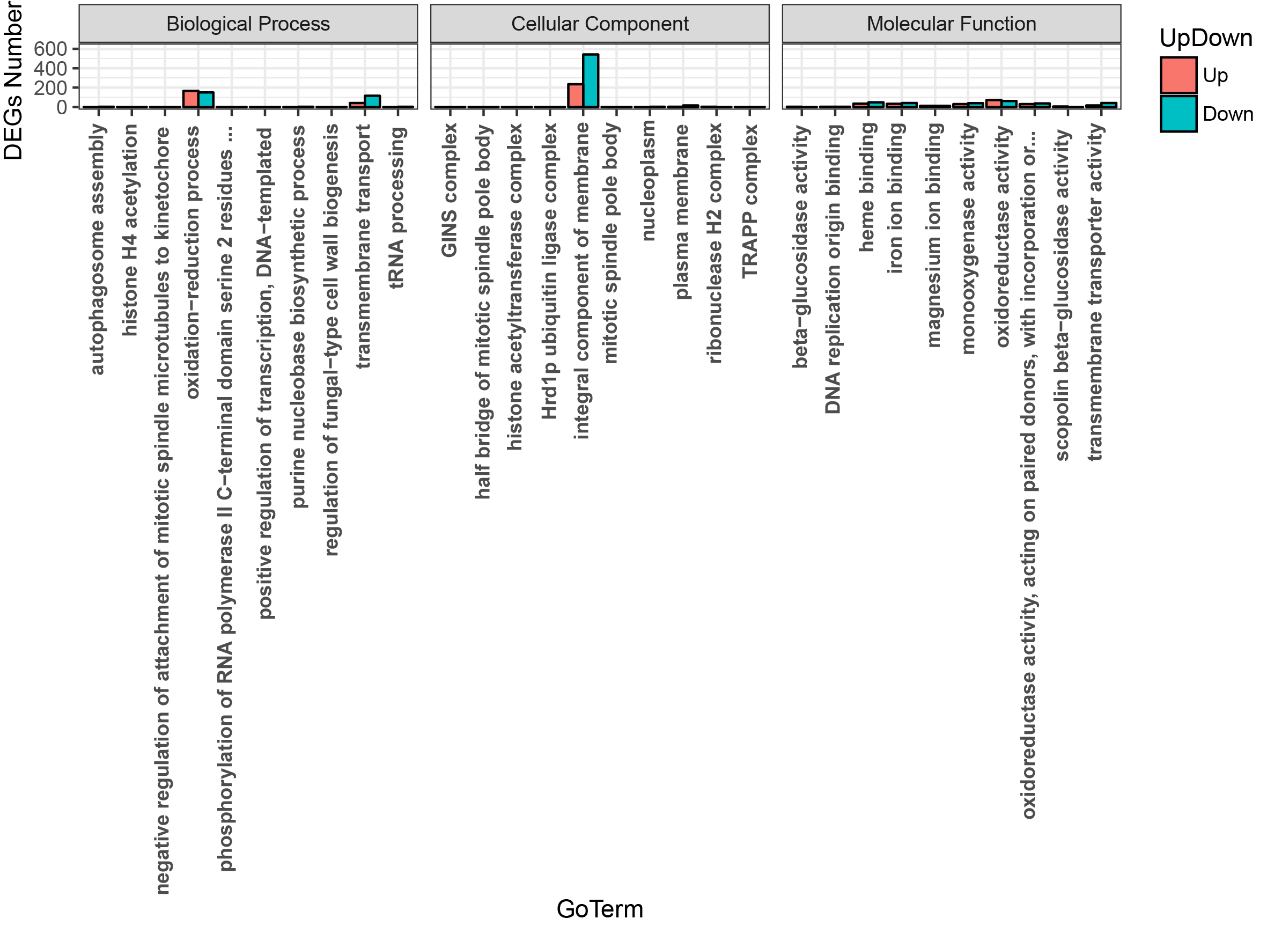


**Supplementary Figure S12c. DEGs (comparison between EP and MYC) number of the most enriched GO Term.**


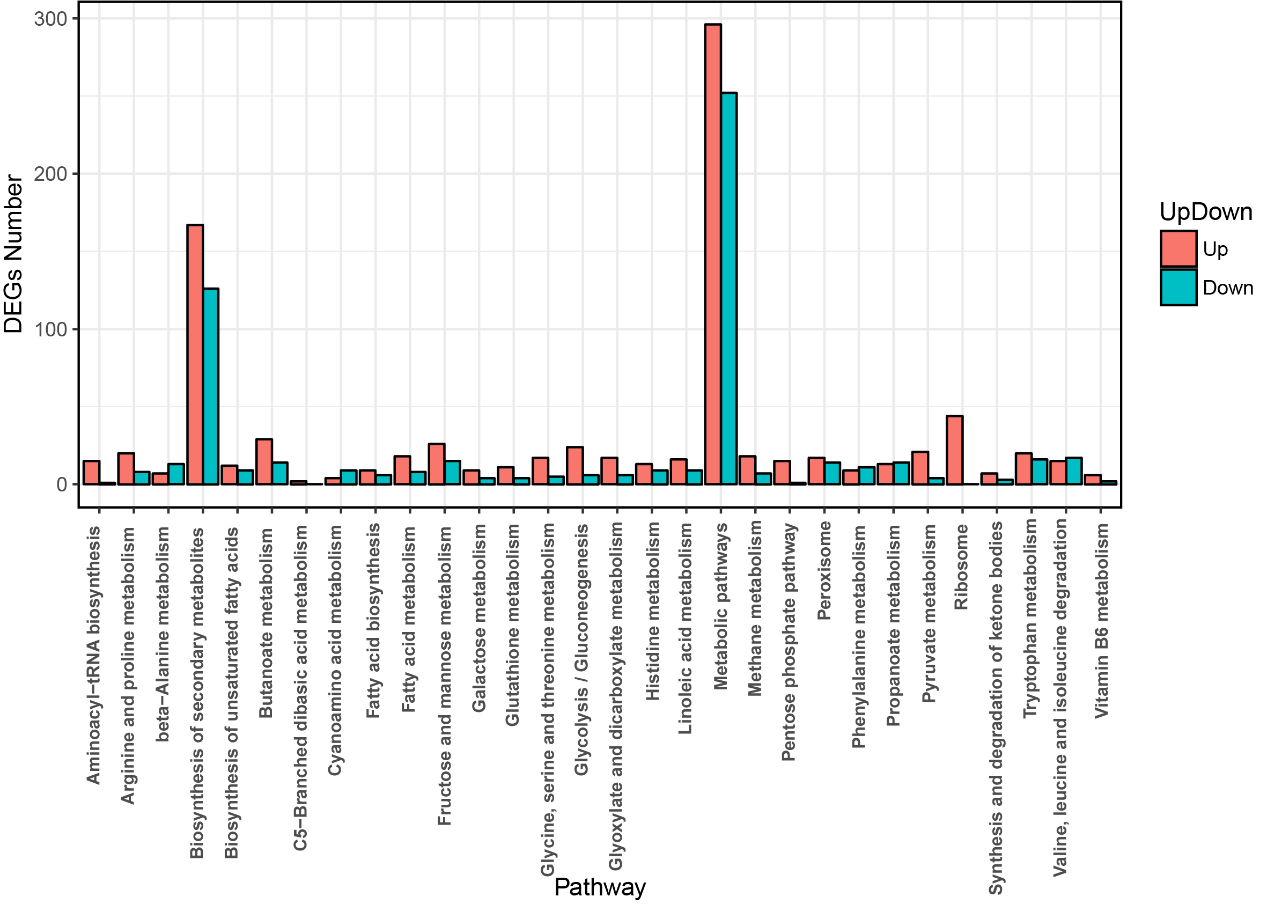


**Supplementary Figure S13a. DEGs (comparison between MYC and IP) number of the most enriched Pathway.**


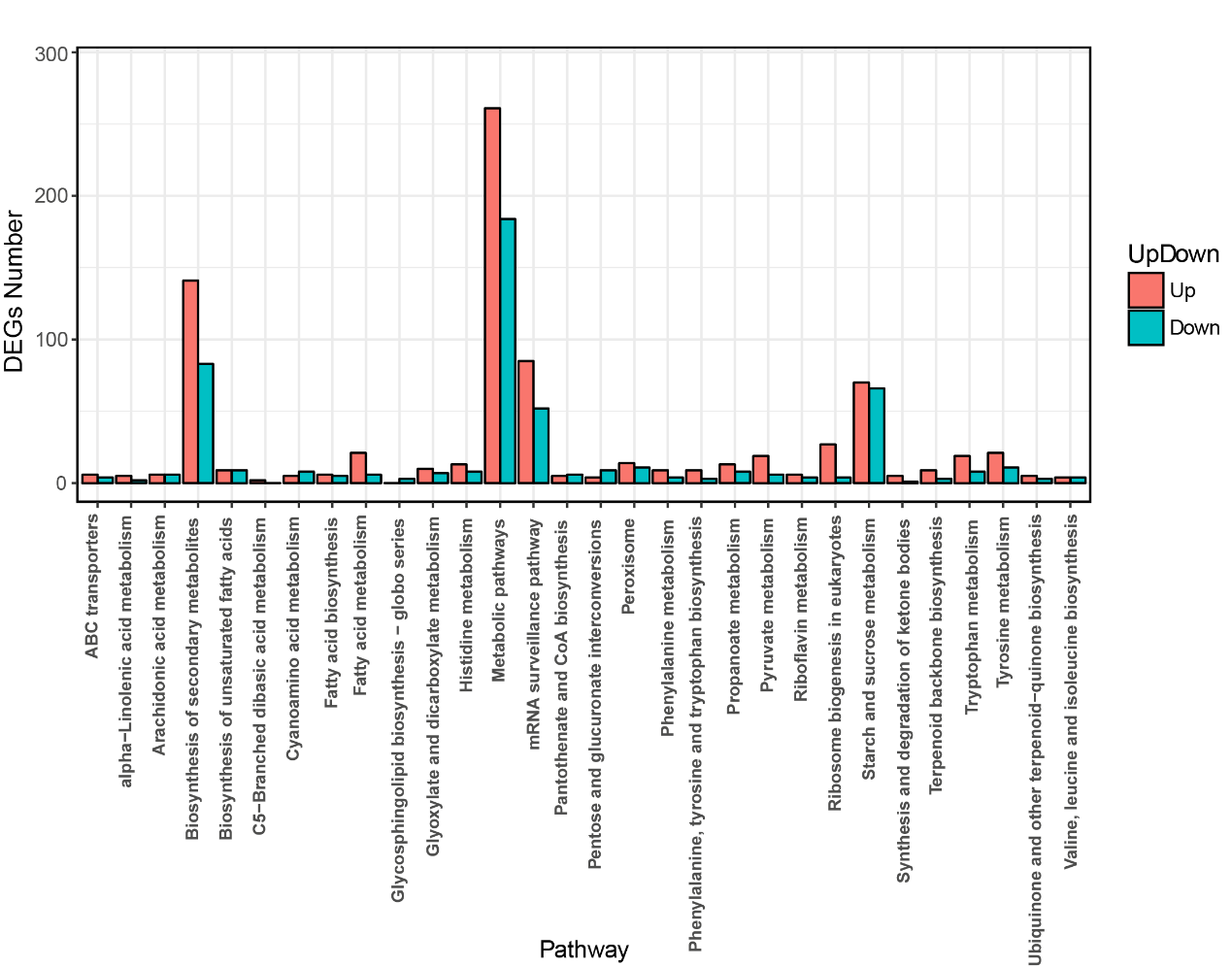


**Supplementary Figure S13b. DEGs (comparison between EP and IP) number of the most enriched Pathway.**


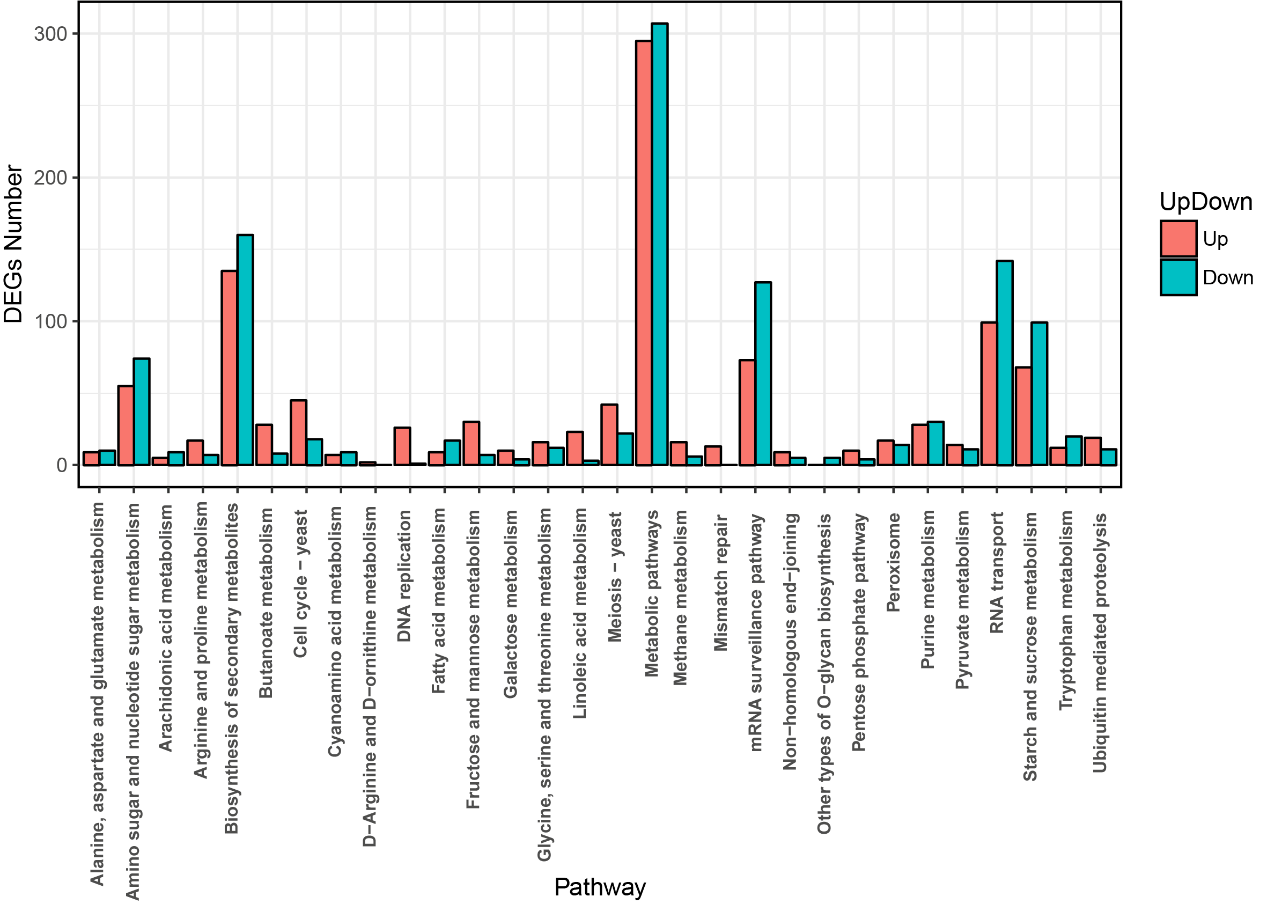


**Supplementary Figure S13c. DEGs (comparison between MYC and EP) number of the most enriched Pathway.**


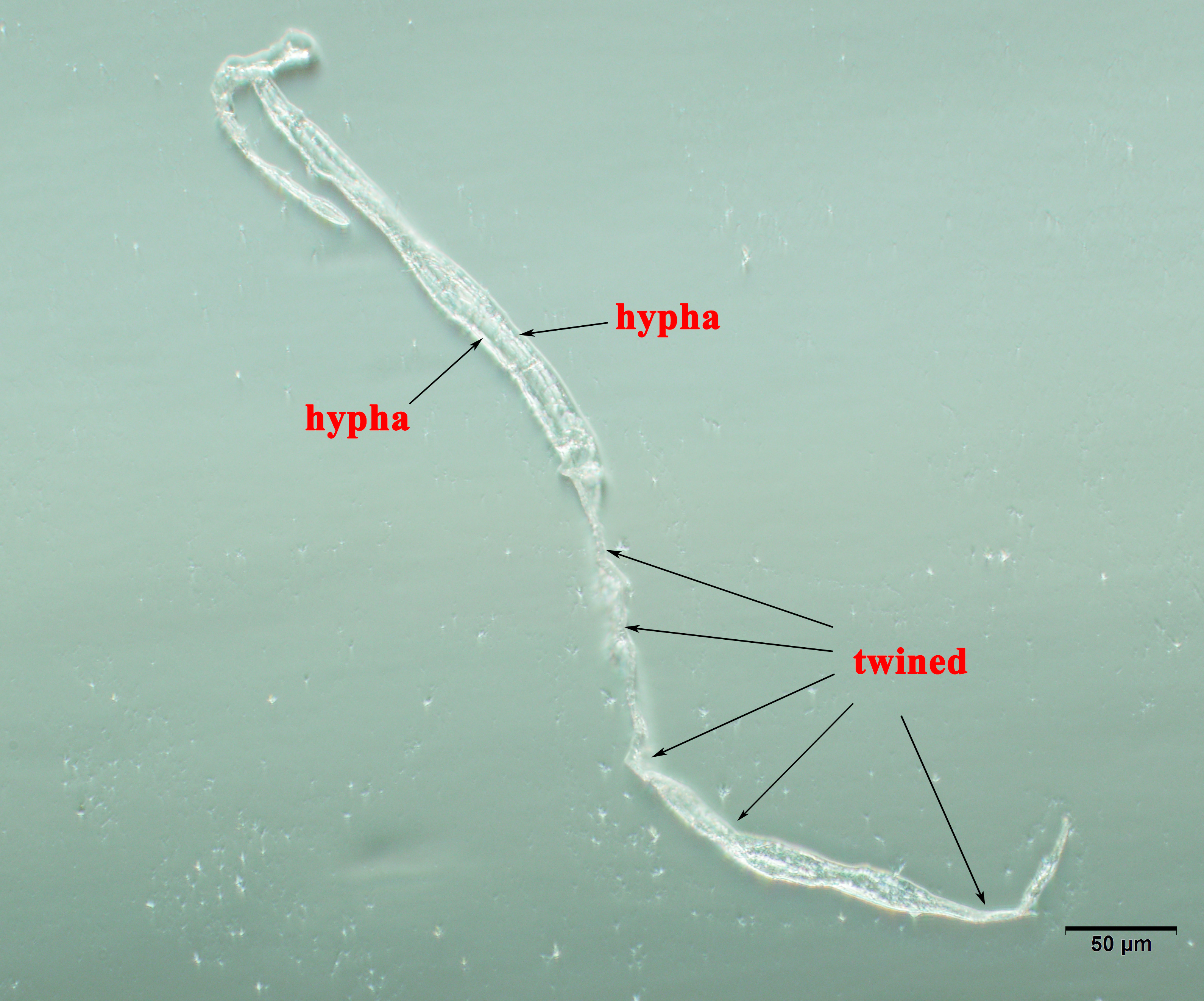


**Supplementary Figure S14. Adhesion multihyphal structures in inner part of *W.cocos* sclerotium**


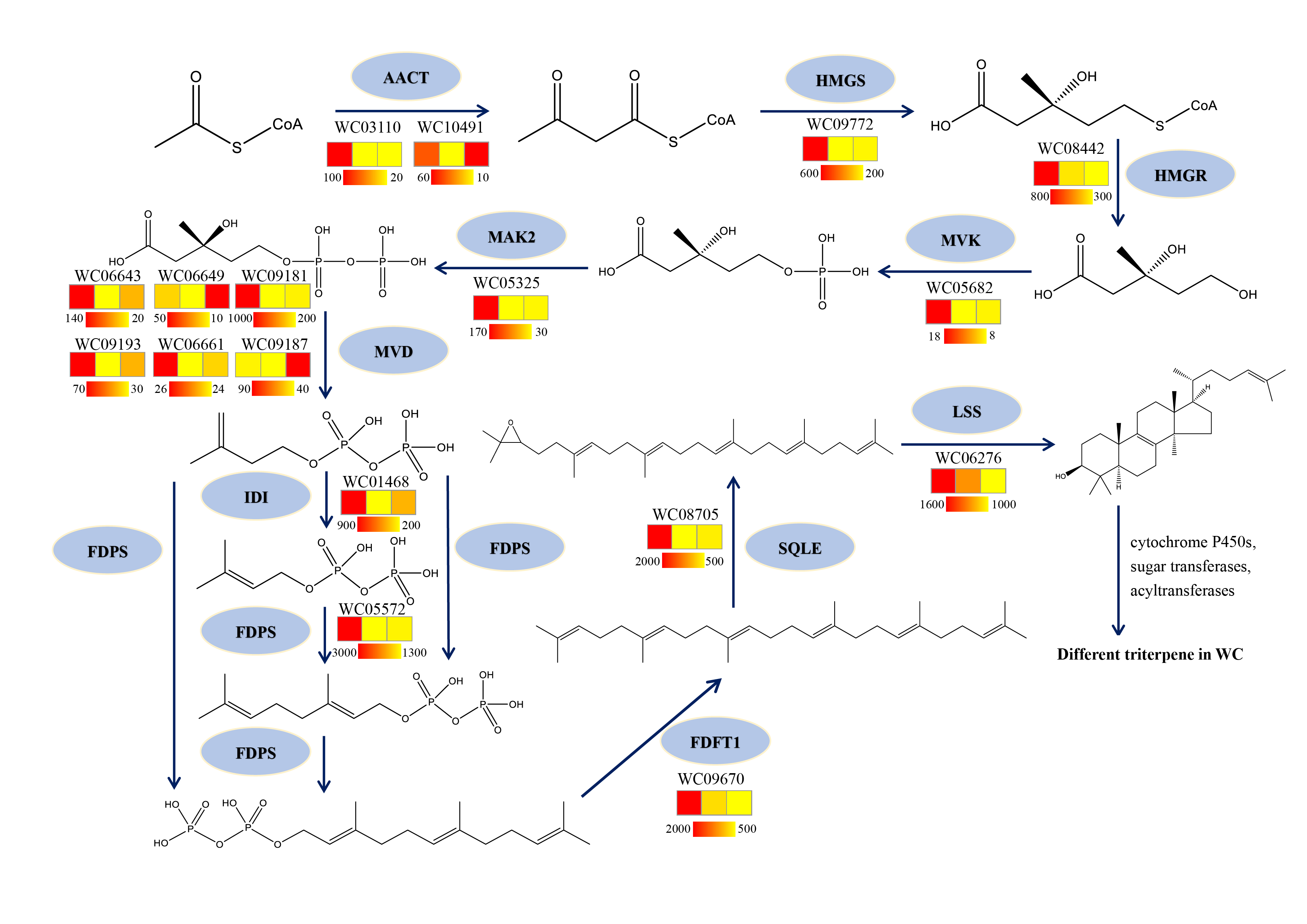


**Supplementary Figure S15.** **The gene expression of Lanosterol biosynthesis pathway.** The heat map labels represent the gene expression of EP, IP and MYC from left to right, and the color from red to yellow means expression level from high to low. and each heatmap is independent of each other, every heatmap has unique scale. The abbreviation and full name of the enzyme can be seen in Supplementary Table S4.

The enzyme’s acronyms used in the figure are: AACT: acetyl CoA: acetyl CoA C-acetyltransferase; HMGS: 3-hydroxy-3-methylglutaryl CoA synthase; HMGR: 3-hydroxy-3-methylglutaryl CoA reductase; MAK2: Phosphomevalonate kinase; MVD, mevalonate pyrophosphate decarboxylase; IDI, Isopentenyldiphosphate isomerase; FDPS, farnesyl-diphosphate synthase; SQS: squalene synthase; SQLE, squalene monooxygenase; LSS: lanosterol synthase. FDFT1, farnesyl-diphosphate farnesyltransferease.

**
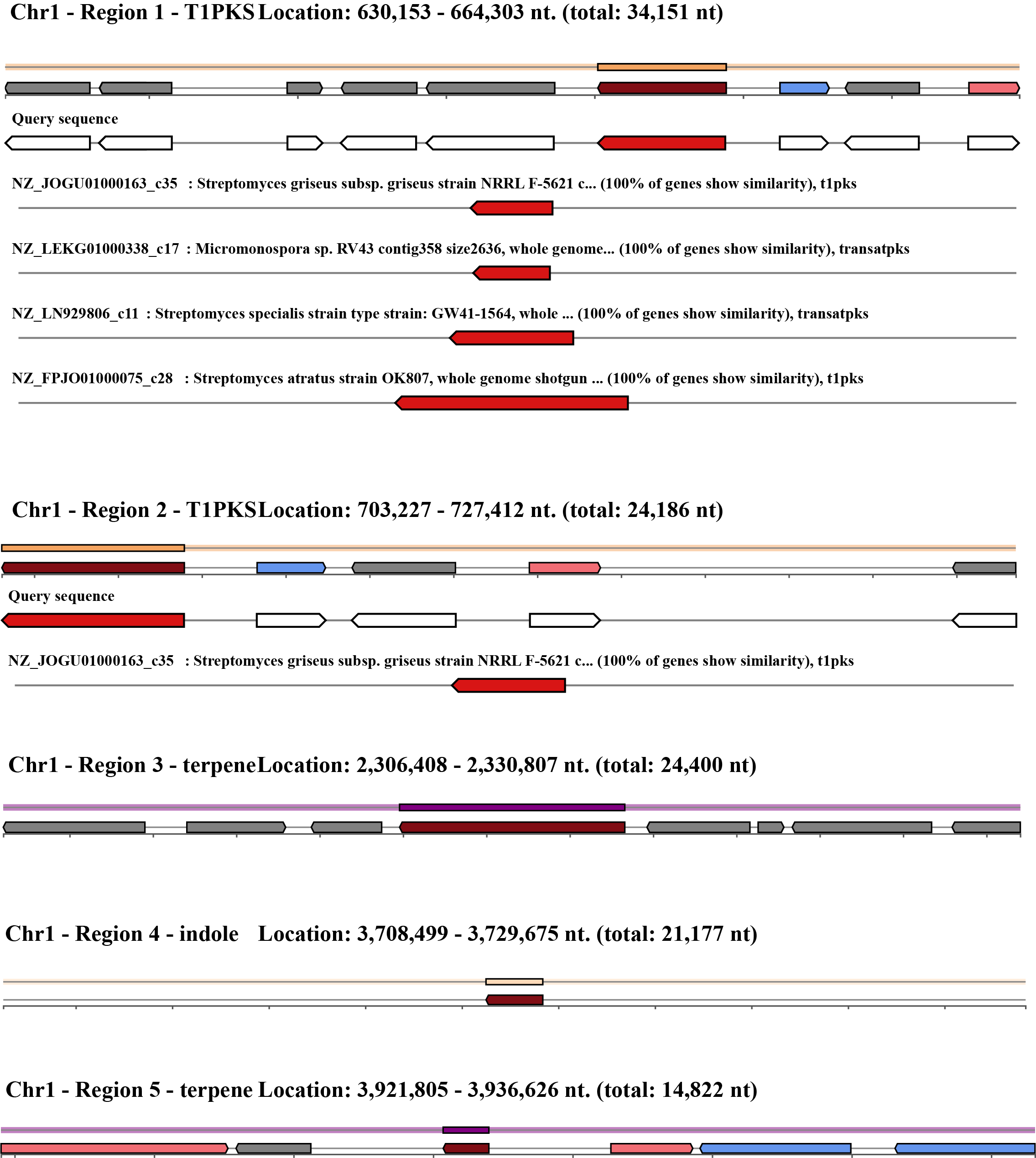

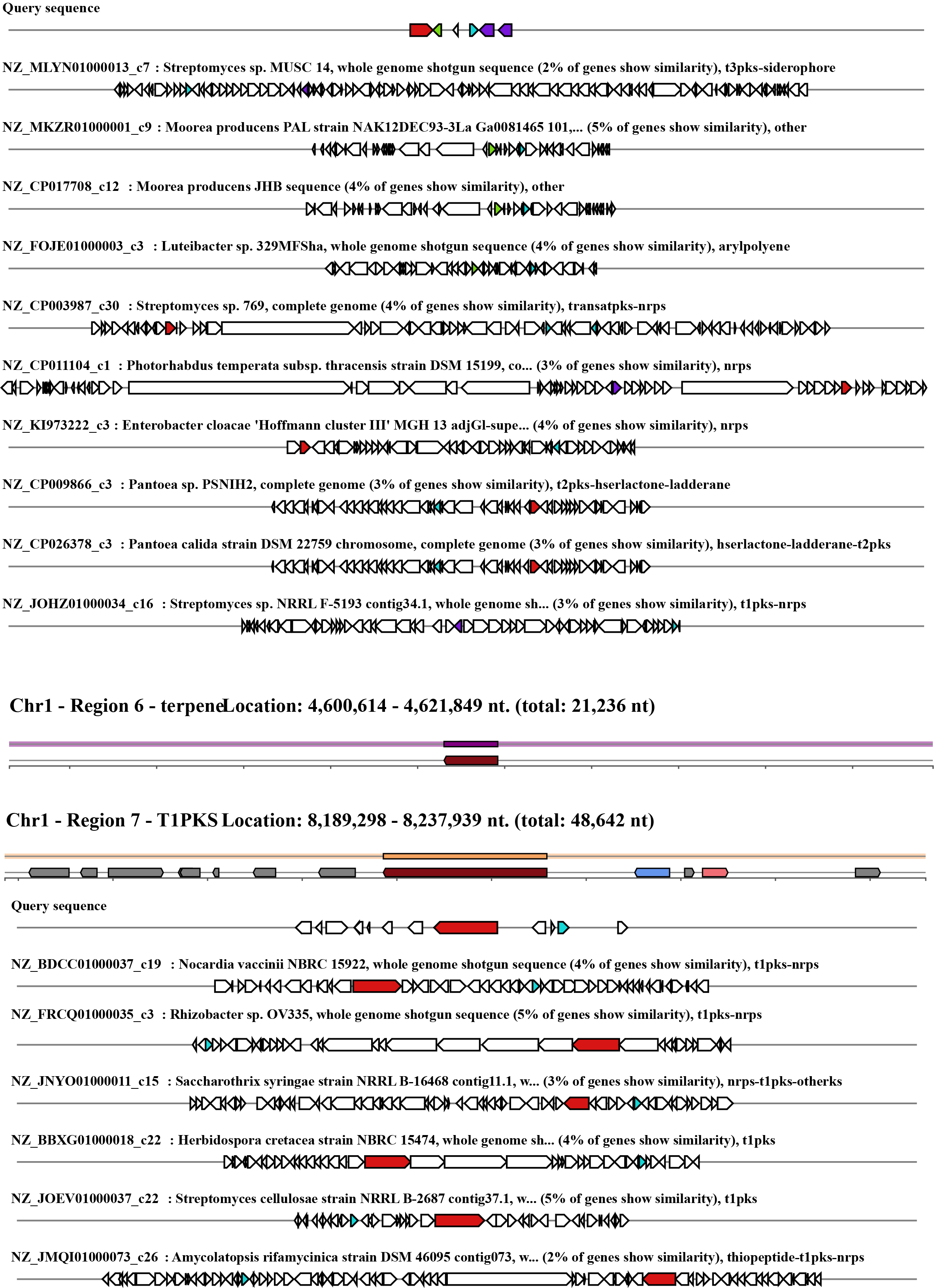
****
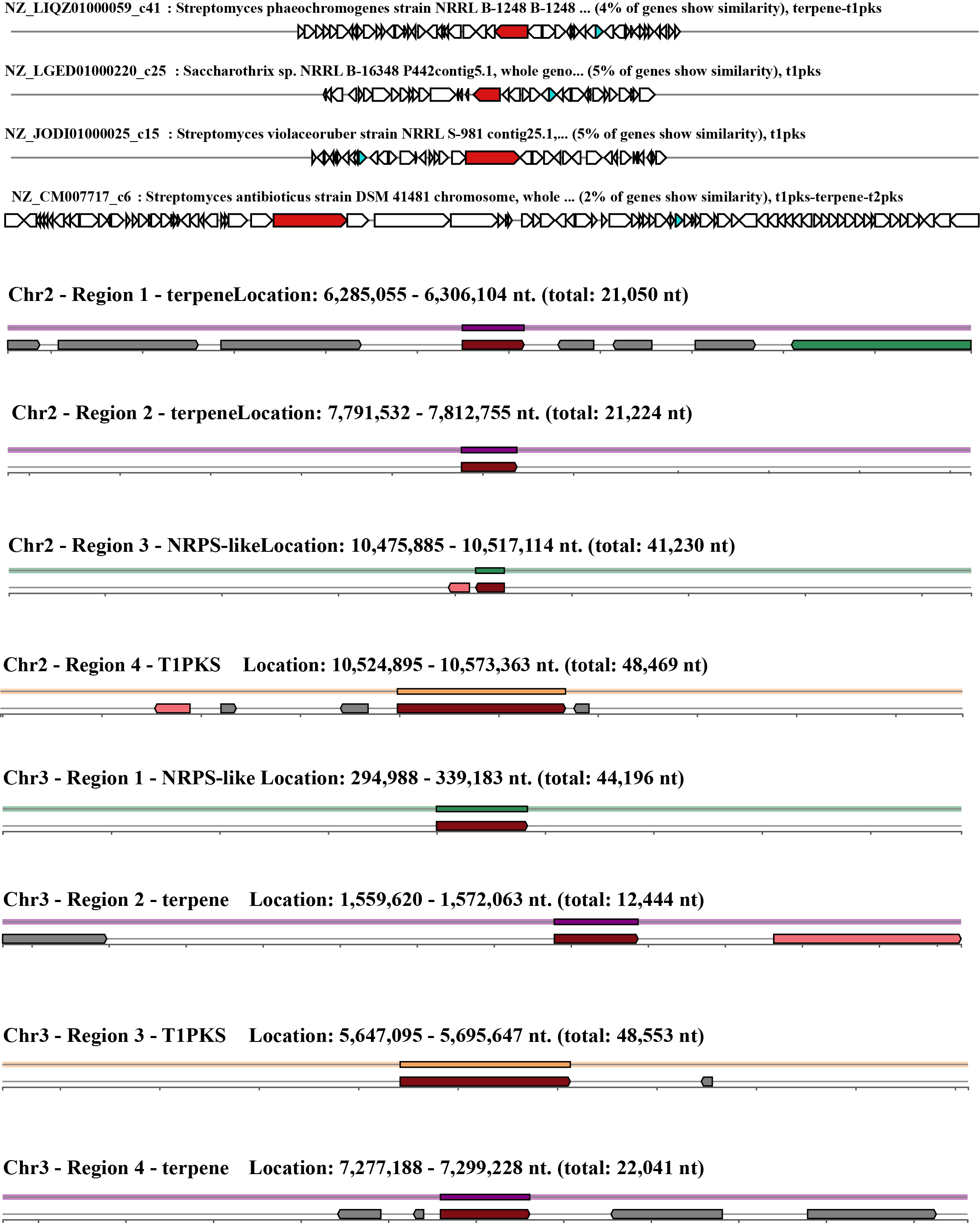

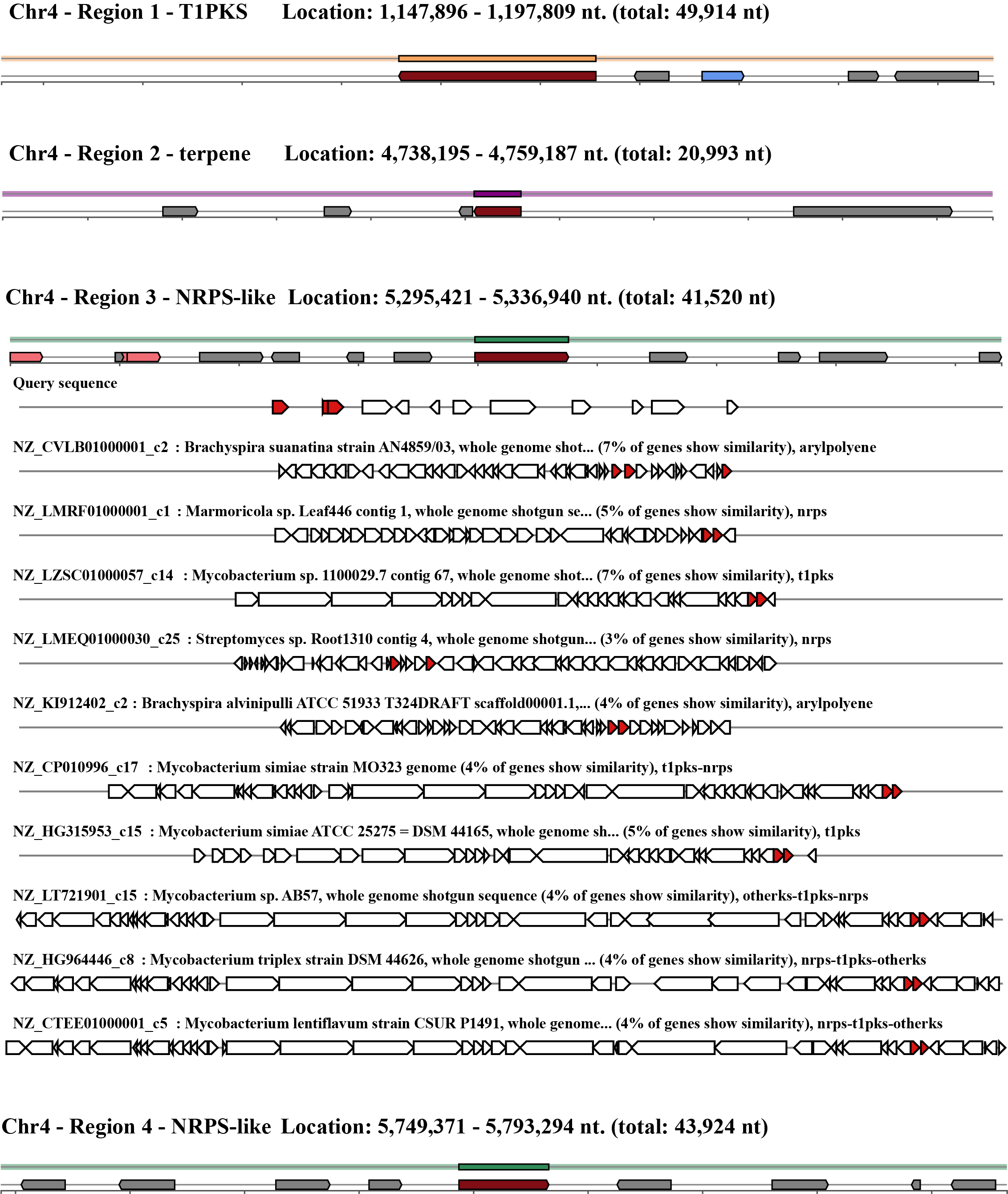

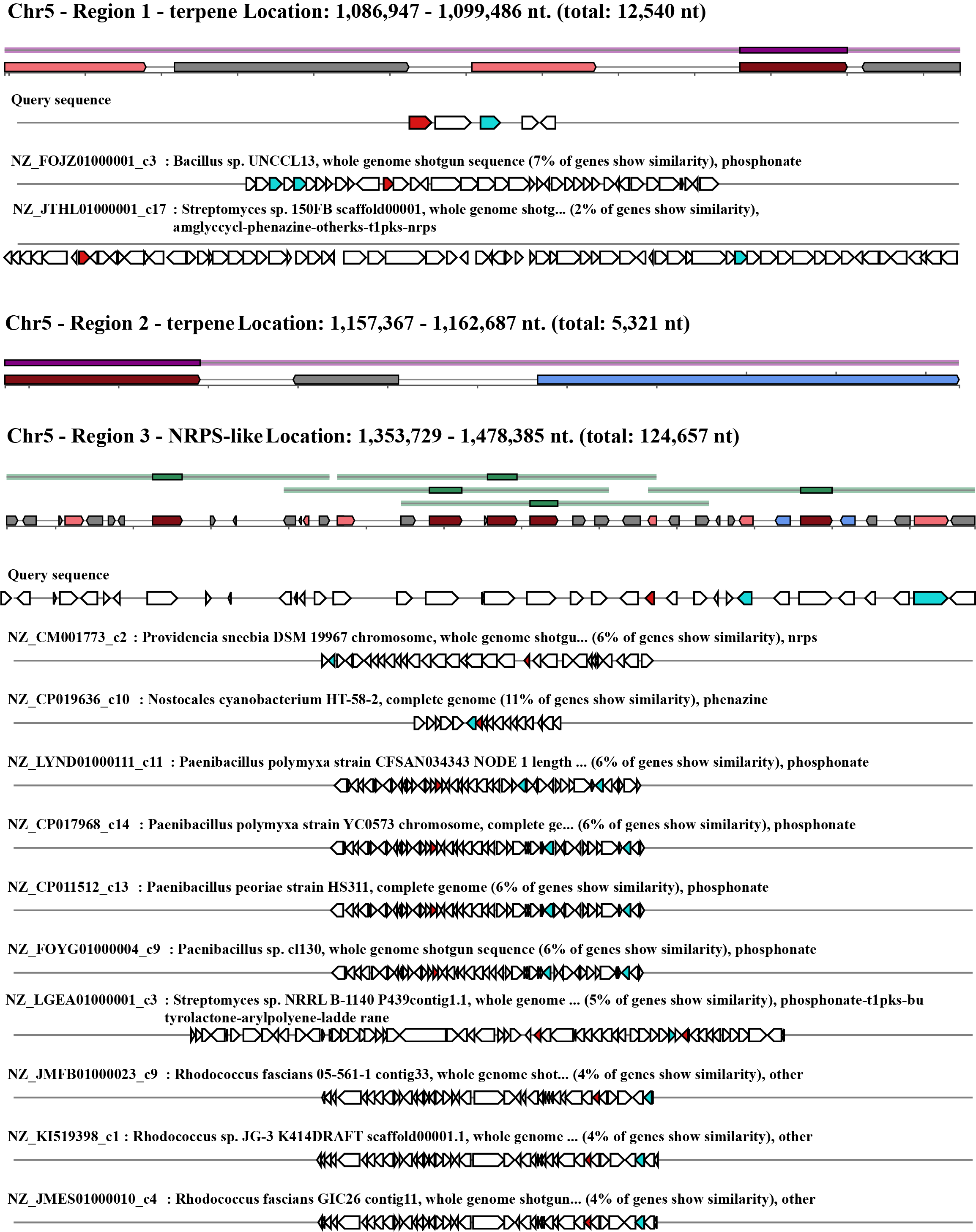
**

**
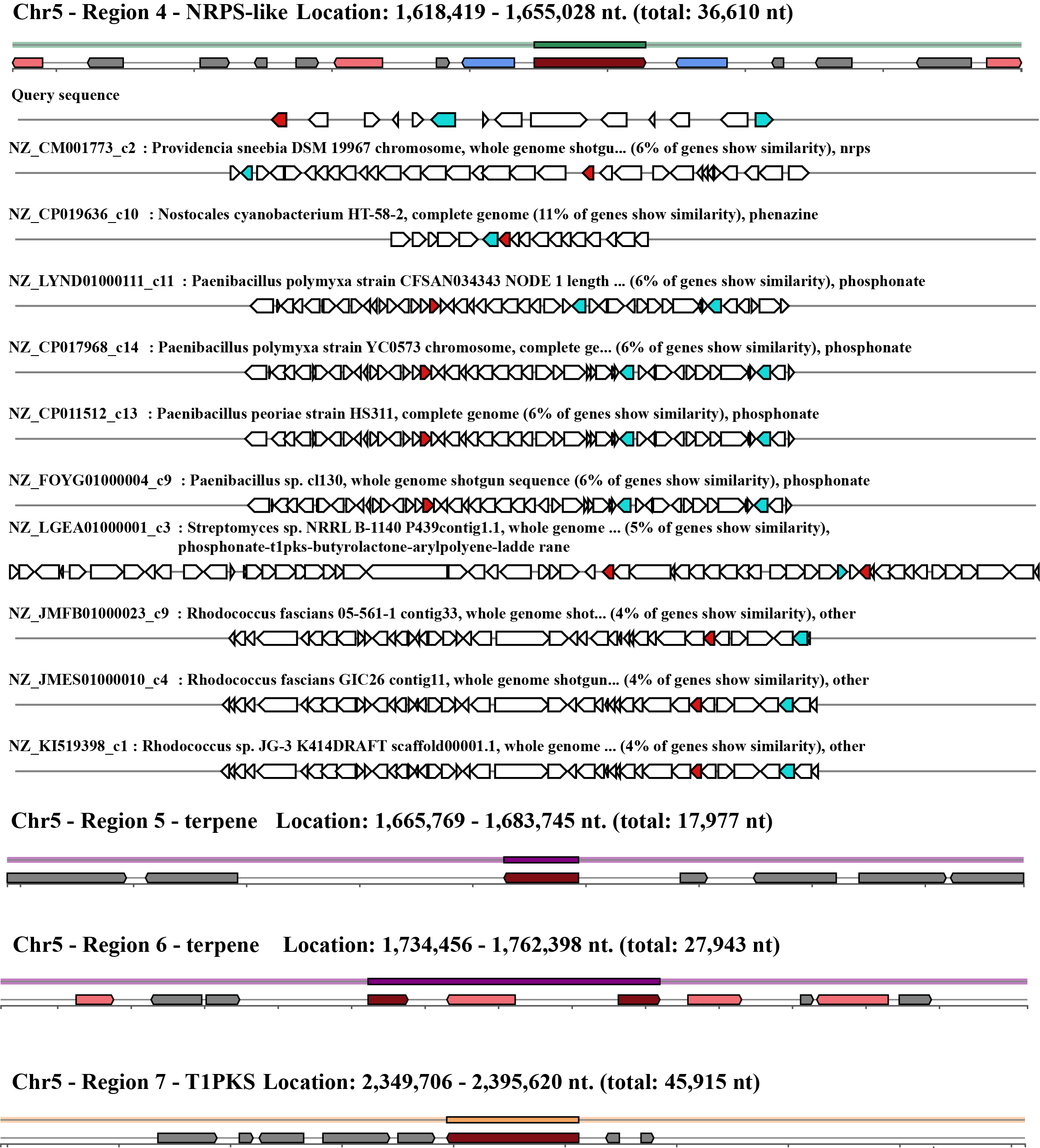

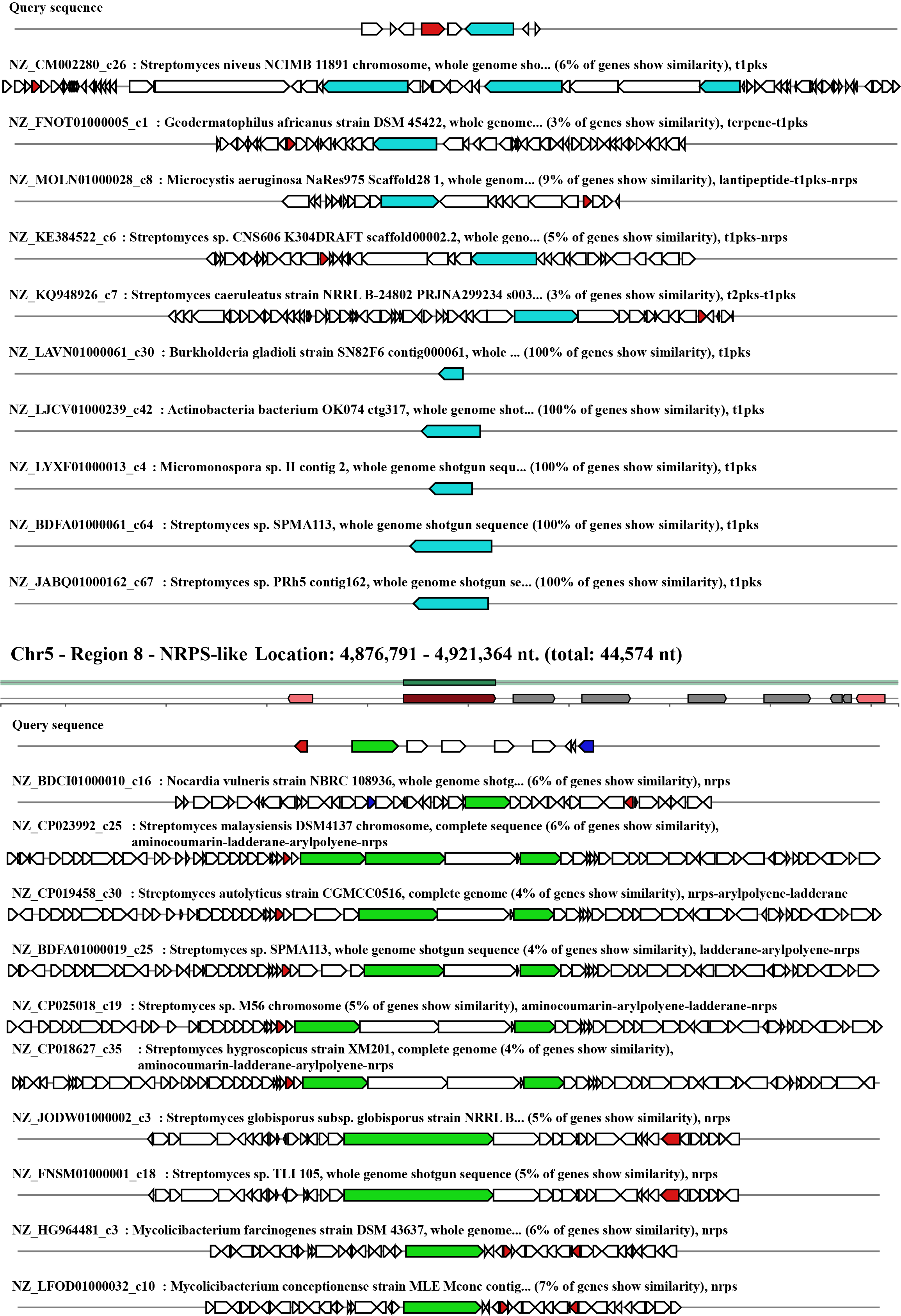

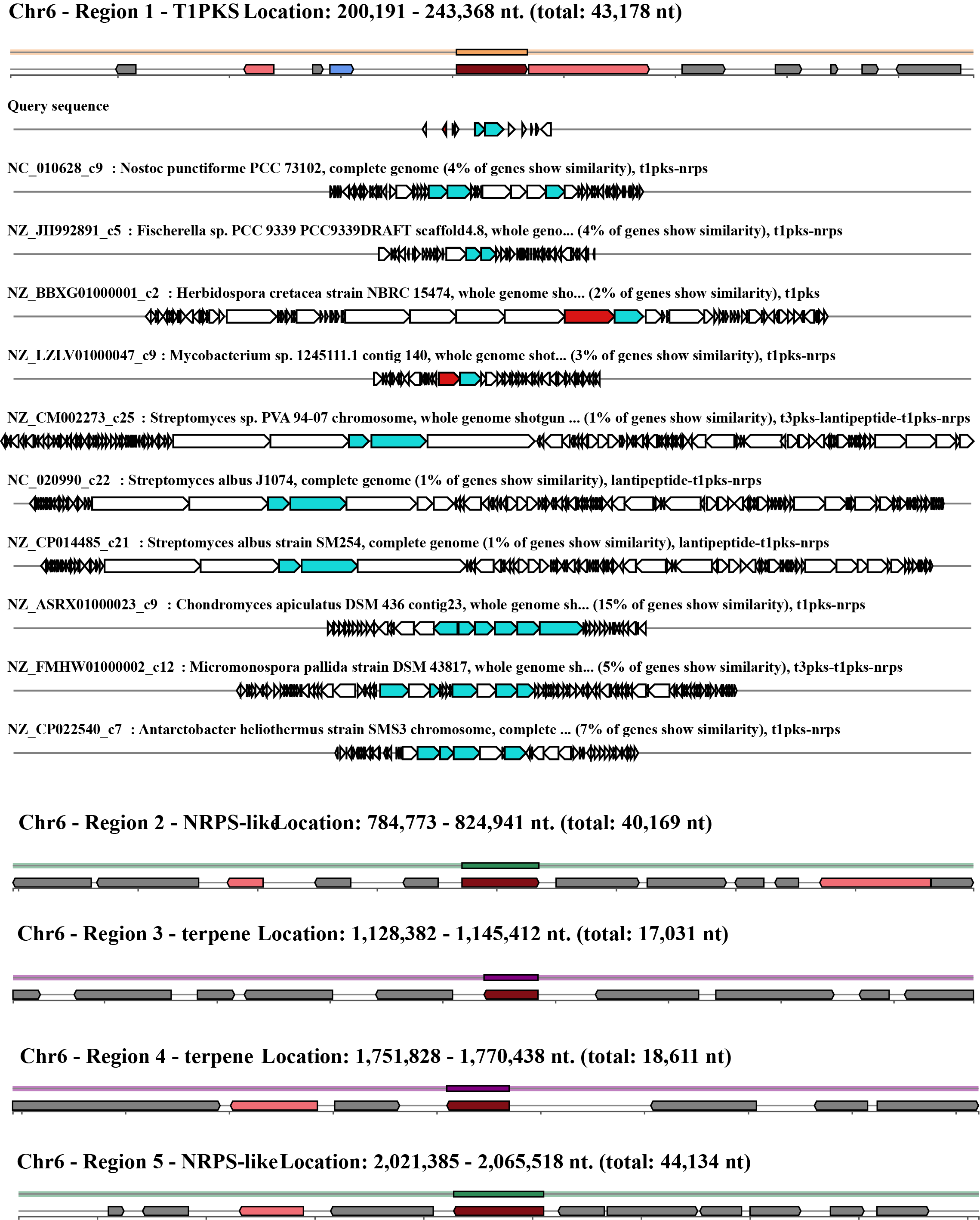

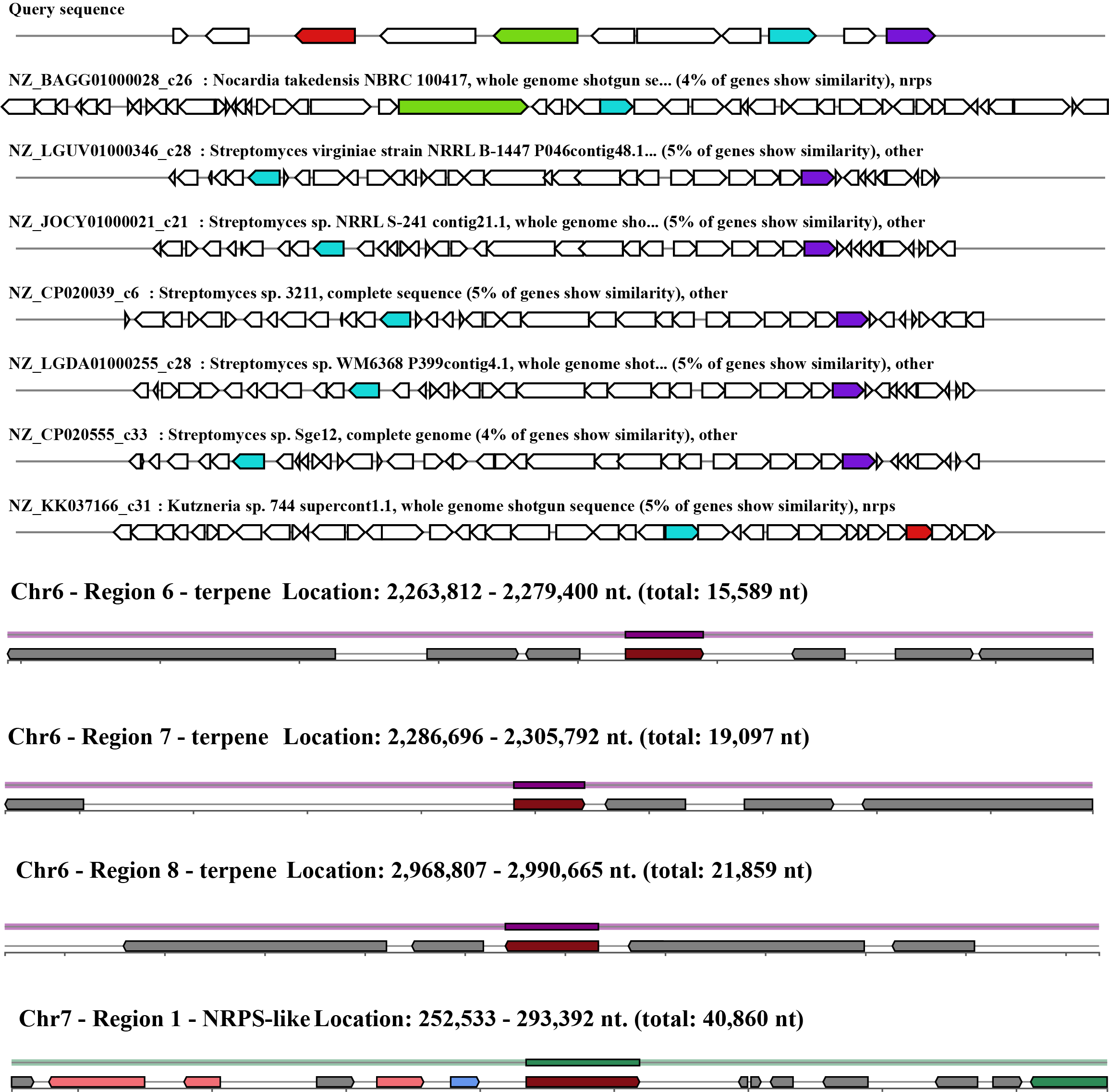
****
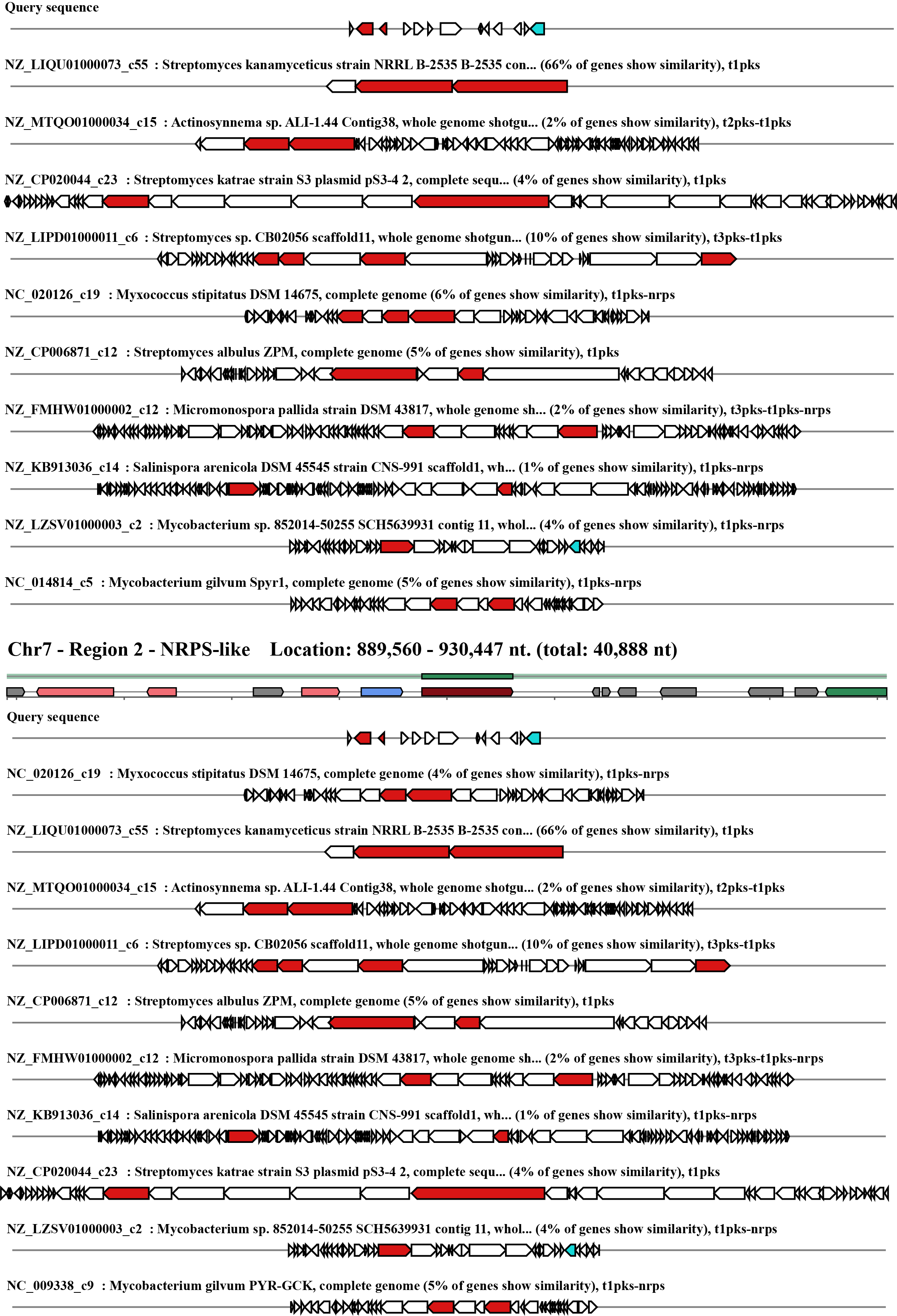

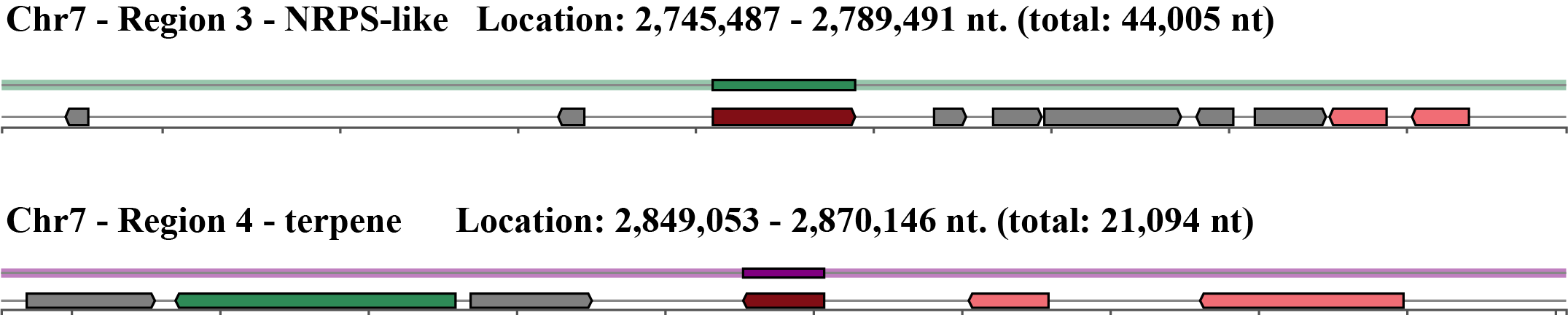
**


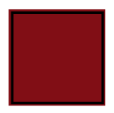

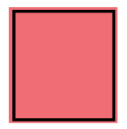

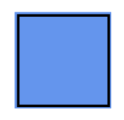

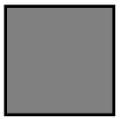

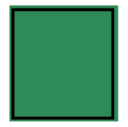
**Supplementary Figure S16. Biosynthetic gene clusters in the *W. cocos* genome.** The predicted functions genes are labeled: core biosynthetic genes ( ), additional biosynthetic genes ( ), transport-related genes ( ), regulatory genes ( ) and other genes ( ).


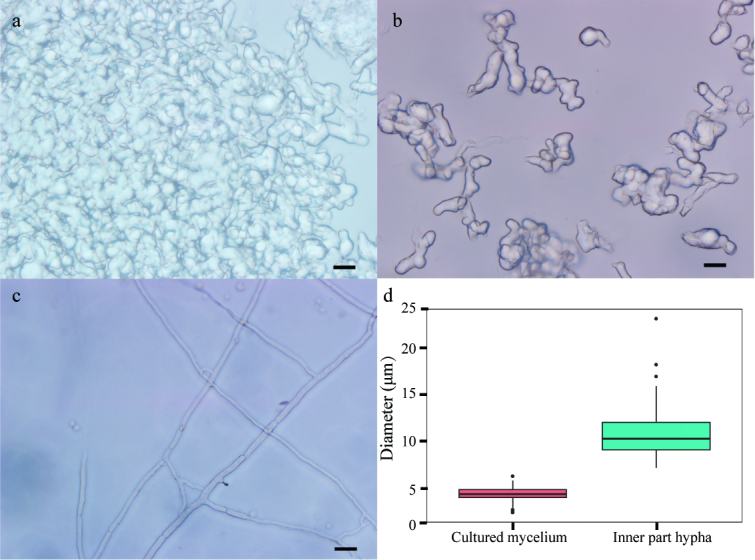


**Supplementary Figure S17. The microscopic morphology of inner part of sclerotium of *W. cocos.*** (a) Morphology of internal organization of sclerotium under 40x light microscope. (b) Scattered internal organization of sclerotium under light microscope. (c) *W. cocos* normal mycelium cultured in plate. (d) A comparison of the diameters of mycelium grew in pates and from sclerotium. Bar, 20 μm.

**SUPPLEMENTARY TABLES**

**Supplementary Table S1. LAI of seven assembled chromosome.**

| **Chromosome** | Chr1 | Chr2 | Chr3 | Chr4 | Chr5 | Chr6 | Chr7 |
| --- | --- | --- | --- | --- | --- | --- | --- |
| **LAI** | 24.9 | 21.85 | 22.88 | 24.18 | 21.64 | 29.06 | 26.67 |

**Supplementary Table S2. Length of different encoding proteins in the fungal mitochondrial genome.**

|  | Wolfiporia cocos | Ganoderma lucidum | Ganodermasinense | Trametes cingulata | Metarhizium anisopliae | Smittium culiseta |
| --- | --- | --- | --- | --- | --- | --- |
| Cox1 | 491 | 528 | 528 | 528 | 526 | 535 |
| Cox2 | 329 | 251 | 251 | 251 | 249 | 254 |
| Cox3 | 269 | 269 | 269 | 269 | 269 | 260 |
| Nad1 | 338 | 338 | 338 | 338 | 374 | 323 |
| Nad2 | 505 | 502 | 502 | 499 | 559 | 479 |
| Nad3 | 121 | 119 | 119 | 130 | 137 | 113 |
| Nad4 | 492 | 454 | 454 | 484 | 484 | 484 |
| Nad4l | 84 | 88 | 88 | 160 | 90 | 102 |
| Nad5 | 654 | 660 | 660 | 679 | 686 | 649 |
| Nad6 | 203 | 203 | 203 | 203 | 225 | 170 |
| cob | 416 | 385 | 385 | 370 | 390 | 396 |
| Atp6 | 256 | 257 | 257 | 257 | 261 | 248 |
| Atp8 | 51 | 52 | 52 | 52 | 55 | 50 |
| Atp9 | 88 | 73 | 73 | 86 | 74` | 73 |
|  | Trametes hirsuta | Antrodia camphorata | Lactarius hatsudake | Hannaella oryzae | Agrocybe aegerita | Tilletia  indica |
| Cox1 | 528 | 528 | 535 | 529 | 527 | 528 |
| Cox2 | 251 | 251 | 251 | 253 | 253 | 258 |
| Cox3 | 269 | 270 | 272 | 276 | 269 | 279 |
| Nad1 | 338 | 338 | 338 | 329 | 338 | 323 |
| Nad2 | 501 | 564 | 549 | 515 | 535 | 597 |
| Nad3 | 119 | 128 | 124 | 125 | 137 | 128 |
| Nad4 | 484 | 482 | 483 | 481 | 482 | 457 |
| Nad4l | 88 | 88 | 90 | 95 | 88 | 88 |
| Nad5 | 1130 | 663 | 664 | 671 | 691 | 684 |
| Nad6 | 201 | 205 | 203 | 204 | 215 | 294 |
| cob | 337 | 383 | 386 | 389 | 387 | 398 |
| Atp6 | 257 | 261 | 258 | 256 | 263 | 261 |
| Atp8 | 52 | 52 | 52 | 48 | 52 | 48 |
| Atp9 | 92 | 73 | 73 | 72 | 73 | 73 |

**Supplementary Table S3. Expression of ATP synthase encoding genes in different tissues of *Wolfiporia cocos***

| **Gene ID** | **MYC** | **IP** | **EP** | **Description** |
| --- | --- | --- | --- | --- |
| WC06527 | 346.11 | 184.23 | 159.14 | ATP synthase |
| WC00663 | 289.70 | 213.93 | 194.03 | ATP synthase subunit f, mitochondrial |
| WC05404 | 398.15 | 208.03 | 205.32 | ATP synthase subunit O, mitochondrial |
| WC09334 | 1024.63 | 474.48 | 374.53 | ATP synthase subunit delta, mitochondrial |
| WC07799 | 95.25 | 49.25 | 46.49 | ATP synthase subunit g, mitochondrial |
| WC01193 | 325.44 | 130.74 | 100.34 | vacuolar ATP synthase |
| WC02096 | 143.22 | 113.66 | 113.70 | vacuolar ATP synthase subunit D |
| WC11399 | 96.86 | 64.61 | 99.70 | vacuolar ATP synthase proteolipid subunit |
| WC01673 | 217.37 | 182.98 | 326.16 | ATP synthase F1 beta subunit |
| WC02710 | 466.43 | 300.67 | 246.42 | ATP synthase d subunit |
| WC04118 | 109.14 | 36.13 | 37.30 | ATP synthase F1 gamma subunit |
| WC06179 | 434.68 | 325.31 | 279.87 | ATP synthase F1 alpha subunit |

**Supplementary Table S4. Key genes of fungal lanosterol synthesis pathway in *Wolfiporia cocos.***

| **Gene ID** | **Name of Gene** | **Abbreviations** | **Accession number** | **Organisam** | |
| --- | --- | --- | --- | --- | --- |
| WC03110 | acetyl-CoA  acetyltransferase | AACT | RDB22486.1 | | Hypsizygus marmoreus |
| WC10491 | acetyl-CoA  acetyltransferase | AACT | PCH44012.1 | | Wolfiporia cocos MD-104 SS10 |
| WC09772 | 3-hydroxy-3-methylglutaryl-CoA synthase | HMGS | PIL24742.1 | | Ganoderma sinense ZZ0214-1 |
| WC08442 | 3-hydroxy-3-methylglutaryl-CoA reductase | HMGR | PIL27711.1 | | Ganoderma sinense ZZ0214-1 |
| WC05682 | Mevalonate kinase | MK | PIL35742.1 | | Ganoderma sinense ZZ0214-1 |
| WC05325 | Phosphomevalonate kinase | MAK2 | KZT08314.1 | | Laetiporus sulphureus 93-53 |
| WC09193  WC06649  WC09181 | mevalonate pyrophosphate decarboxylase | MVD | ALI96784.1 | | Taiwanofungus camphoratus |
| WC06643  WC09187  WC06661 | mevalonate pyrophosphate decarboxylase | MVD | PIL35998.1 | | Ganoderma sinense ZZ0214-1 |
| WC01468 | Isopentenyldiphosphate isomerase | IDI | PCH33661.1 | | Wolfiporia cocos MD-104 SS10 |
| WC05572 | farnesyl-diphosphate synthase | FDPS | AFR13039.1 | | Wolfiporia cocos |
| WC09670 | squalene synthase | SQS | AFR13032.1 | | Wolfiporia cocos |
| WC08705 | squalene monooxygenase | SQLE | KZT21548.1 | | Neolentinus lepideus HHB14362 ss-1 |
| WC06276 | 2,3-oxidosqualene- lanosterol cyclase | LSS | PIL24816.1 | | Ganoderma sinense ZZ0214-1 |

**Supplementary Table S5. The qRT-PCR results of CYPs.**

|  | Family | EP | IP | MYC | Correlation (R) |
| --- | --- | --- | --- | --- | --- |
| WC06276 | LSS | 0.168019 | 0.042651 | 0.022192 | 1 |
| WC05906 | CYP512CF1 | 0.044502 | 0.009753 | 0.0041 | 1 |
| WC07850 | CYP63A86 | 0.074842 | 0.017458 | 0.00849 | 0.999989 |
| WC09072 | CYP5037BD1 | 0.016863 | 0.002339 | 0.000143 | 0.999965 |
| WC08328 | CYP5027B10 | 0.08362 | 0.019915 | 0.01038 | 0.999954 |
| WC06071 | CYP5350G1 | 0.168404 | 0.032577 | 0.008032 | 0.999916 |
| WC04847 | CYP512N9 | 0.009958 | 0.002981 | 0.00146 | 0.999237 |
| WC06856 | CYP5139D35 | 0.070316 | 0.018453 | 0.006346 | 0.998773 |
| WC06799 | CYP5139D | 0.368567 | 0.047366 | 0.018326 | 0.9985 |
| WC08092 | CYP6384A1 | 0.018841 | 0.002524 | 0.001099 | 0.998364 |
| WC03745 | CYP5341B9 | 0.008431 | 0.002123 | 0.001642 | 0.997821 |
| WC02438 | CYP5153M1 | 0.10083 | 0.012691 | 0.006896 | 0.997227 |
| WC06158 | CYP53D10 | 0.05329 | 0.017948 | 0.016516 | 0.995474 |
| WC08335 | CYP5027B12 | 1 | 0.054788 | 0.02836 | 0.994421 |
| WC06190 | CYP53D16 | 3.97237 | 0.598739 | 0.528509 | 0.993766 |
| WC09933 | CYP5149F1 | 0.00357 | 0.000131 | 6.77E-05 | 0.993535 |
| WC09071 | CYP5037BC4 | 0.108819 | 0.003645 | 0.001861 | 0.993396 |
| WC08665 | CYP512M2 | 2.549121 | 0.08362 | 0.057512 | 0.992756 |
| WC09716 | CYP6005A10 | 0.486327 | 0.006944 | 0.010237 | 0.99083 |
| WC06906 | CYP5037BC3 | 0.668964 | 0.001325 | 0.006434 | 0.990738 |
| WC09172 | CYP5348AD8 | 0.279322 | 0.008729 | 0.010972 | 0.990662 |
| WC09073 | CYP5037BD2 | 0.135842 | 0.000237 | 0.001654 | 0.990403 |
| WC06905 | CYP5037BC2 | 0.002228 | 1.94E-05 | 4.29E-05 | 0.990382 |
| WC04972 | CYP512P9 | 0.817902 | 0.011049 | 0.024861 | 0.989577 |
| WC06171 | CYP53D11 | 0.019777 | 0.006848 | 0.007139 | 0.98888 |
| WC07263 | CYP5348AE1 | 0.230047 | 0.068393 | 0.073812 | 0.987374 |
| WC01717 | CYP5150AV1 | 0.196146 | 0.004776 | 0.011842 | 0.986887 |
| WC05840 | CYP5150AU1 | 0.05329 | 0.020761 | 0.022097 | 0.986277 |
| WC05577 | CYP5150AS1 | 0.110338 | 0.012259 | 0.016631 | 0.98575 |
| WC00047 | CYP51F14 | 0.010309 | 0.000918 | 0.001372 | 0.98517 |
| WC10300 | CYP63B25 | 0.524858 | 0.01937 | 0.045437 | 0.984663 |
| WC04940 | CYP512P10 | 0.036906 | 0.026278 | 0.021493 | 0.984099 |
| WC04233 | CYP5354A | 0.133972 | 0.002981 | 0.011125 | 0.982923 |
| WC06775 | CYP5037BC1 | 0.042101 | 0.010453 | 0.01278 | 0.980937 |
| WC08786 | CYP5350F3 | 0.068393 | 0.031907 | 0.013697 | 0.979372 |
| WC07444 | CYP5035F5 | 0.038473 | 0.003521 | 0.00657 | 0.978366 |
| WC08451 | CYP5037B83 | 0.145592 | 0.002275 | 0.016064 | 0.976575 |
| WC04971 | CYP512P8 | 0.125869 | 0.003721 | 0.015625 | 0.976321 |
| WC03746 | CYP5341B9 | 0.017098 | 0.000589 | 0.002259 | 0.975554 |
| WC00716 | CYP537F3 | 0.046071 | 0.000492 | 0.005336 | 0.974468 |
| WC03390 | CYP5141A34 | 0.064257 | 0.003496 | 0.009958 | 0.974452 |
| WC09067 | CYP5348AH2 | 0.041235 | 0.000509 | 0.005759 | 0.969326 |
| WC02103 | CYP5144 | 0.012174 | 0.005921 | 0.002093 | 0.9674 |
| WC05402 | CYP5035F6 | 0.04095 | 0.007139 | 0.01176 | 0.96743 |
| WC06191 | CYP53D16 | 0.013888 | 0.009099 | 0.006045 | 0.964564 |
| WC09060 | CYP5348AB1 | 0.021051 | 0.003331 | 0.00639 | 0.957743 |
| WC06229 | CYP5037B85 | 0.140632 | 0.019641 | 0.043889 | 0.949201 |
| WC04981 | CYP5139D36 | 0.059129 | 0.039555 | 0.024349 | 0.948713 |
| WC04773 | CYP5350F1 | 0.154963 | 0.041235 | 0.065154 | 0.945926 |
| WC04990 | CYP5139D34 | 0.132127 | 0.04095 | 0.062935 | 0.934947 |
| WC08122 | CYP512CH1 | 0.104386 | 0.002455 | 0.028557 | 0.929179 |
| WC04770 | CYP5350F2 | 0.026096 | 0.013792 | 0.017579 | 0.906976 |
| WC06155 | CYP53D9 | 0.082469 | 0.049721 | 0.006258 | 0.889276 |
| WC00711 | CYP5150D32 | 0.001724 | 0.000452 | 0.000892 | 0.888342 |
| WC00714 | CYP5150D37 | 0.026278 | 0.003933 | 0.011679 | 0.88796 |
| WC00967 | CYP5150AR1 | 0.010525 | 0.002705 | 0.005524 | 0.880712 |
| WC08043 | CYP5139D32 | 0.024181 | 0.017458 | 0.020475 | 0.828677 |
| WC08336 | CYP5027B13 | 0.027776 | 0.020054 | 0.024014 | 0.785087 |
| WC03431 | CYP5037BB1 | 0.016746 | 0.002899 | 0.010525 | 0.757271 |
| WC08839 | CYP6384A1 | 0.012869 | 0.002542 | 0.008315 | 0.751012 |
| WC06202 | CYP63S2 | 0.049721 | 0.017824 | 0.035897 | 0.745231 |
| WC09000 | CYP5348AC2 | 0.048698 | 0.042101 | 0.008258 | 0.721373 |
| WC06188 | CYP53D15 | 0.01468 | 0.013985 | 0.007289 | 0.673108 |
| WC09065 | CYP5348AB | 0.032577 | 0.030607 | 0.010598 | 0.670026 |
| WC00710 | CYP5150D32 | 0.578344 | 0.550953 | 0.032129 | 0.642436 |
| WC08483 | CYP53C27 | 0.125869 | 0.023035 | 0.095391 | 0.633999 |
| WC09016 | CYP5732B1 | 0.014579 | 0.000756 | 0.010672 | 0.622336 |
| WC00717 | CYP537F4 | 0.244855 | 0.072293 | 0.20166 | 0.594739 |
| WC00154 | CYP63S1 | 0.017217 | 0.01209 | 0.015953 | 0.5915 |
| WC08025 | CYP5139D37 | 0.015734 | 0.015953 | 0.007705 | 0.589029 |
| WC06691 | CYP502B12 | 0.320856 | 0.078563 | 0.275476 | 0.54058 |
| WC06181 | CYP53D14 | 0.021642 | 0.023196 | 0.009889 | 0.51941 |
| WC08450 | CYP5037B82 | 0.013888 | 0.00176 | 0.012007 | 0.512963 |
| WC01693 | CYP63A85 | 0.024349 | 0.002008 | 0.021051 | 0.506573 |
| WC04970 | CYP512P7 | 0.006346 | 1.12E-05 | 0.005759 | 0.460013 |
| WC09431 | CYP5141A35 | 0.013602 | 0.005013 | 0.013888 | 0.357587 |
| WC06118 | CYP53D7 | 0.03901 | 0.022718 | 0.043889 | 0.17133 |
| WC06801 | CYP5139D38 | 0.042101 | 0.07911 | 0.006045 | 0.121725 |
| WC00534 | CYP5732B2 | 0.011125 | 0.000324 | 0.015517 | 0.109118 |
| WC00535 | CYP5732B2 | 0.011125 | 0.000324 | 0.015517 | 0.109118 |
| WC05819 | CYP5150AU2 | 0.007813 | 0.004275 | 0.009355 | 0.093379 |
| WC06174 | CYP53D12 | 0.00519 | 0.006708 | 0.004129 | 0.027603 |
| WC10212 | CYP512CG2 | 0.006087 | 0.007813 | 0.004979 | 0.004317 |
| WC08995 | CYP5348AC1 | 0.035158 | 0.024518 | 0.042689 | -0.03109 |
| WC04846 | CYP512N8 | 0.036906 | 0.064704 | 0.021493 | -0.03443 |
| WC03690 | CYP5156F1 | 0.008032 | 0.00942 | 0.00734 | -0.06124 |
| WC07617 | CYP51F1 | 0.05366 | 0.102238 | 0.030186 | -0.0689 |
| WC08329 | CYP5027B11 | 0.076415 | 0.148651 | 0.044502 | -0.09023 |
| WC09447 | CYP5140A20 | 0.005759 | 0.006615 | 0.005411 | -0.10917 |
| WC07876 | CYP63A87 | 0.008912 | 0.000456 | 0.018073 | -0.15207 |
| WC10064 | CYP502B12 | 0.20733 | 0.068393 | 0.366021 | -0.16707 |
| WC06798 | CYP5139D39 | 0.005839 | 0.000502 | 0.012604 | -0.1963 |
| WC03388 | CYP5141A36 | 0.001025 | 0.0002 | 0.002137 | -0.21324 |
| WC02957 | CYP5037B81 | 0.057114 | 0.267943 | 0.015953 | -0.23887 |
| WC08334 | CYP5027B | 0.035897 | 0.140632 | 0.021944 | -0.2824 |
| WC05145 | CYP5348AA1 | 0.009291 | 0.002438 | 0.022097 | -0.29804 |
| WC02340 | CYP5152A12 | 0.017098 | 0.003545 | 0.043889 | -0.31151 |
| WC00549 | CYP5138A17 | 0.033032 | 0.233258 | 0.015093 | -0.31448 |
| WC00811 | CYP512P6 | 0.003308 | 0.000237 | 0.00982 | -0.3278 |
| WC06936 | CYP5365G1 | 0.00613 | 0.01278 | 0.00568 | -0.33105 |
| WC01692 | CYP63A84 | 0.009037 | 0.006003 | 0.015625 | -0.33317 |
| WC06175 | CYP53D13 | 0.007239 | 0.006003 | 0.010097 | -0.34706 |
| WC01084 | CYP61A1 | 0.033262 | 0.00176 | 0.106579 | -0.34846 |
| WC04992 | CYP5347A3 | 0.000106 | 0.003217 | 1.34E-05 | -0.36028 |
| WC06146 | CYP53D8 | 0.0017 | 4.38E-05 | 0.006346 | -0.38655 |
| WC00694 | CYP5906Q2 | 0.008088 | 0.125 | 0.010525 | -0.4007 |
| WC08780 | CYP5350A2 | 0.025033 | 0.011438 | 0.066064 | -0.40025 |
| WC04423 | CYP5144FJ1 | 0.045437 | 0.040386 | 0.062068 | -0.41565 |
| WC06088 | CYP512CG1 | 0.314253 | 1.729074 | 0.450625 | -0.46313 |
| WC08188 | CYP5136M3 | 0.027776 | 0.239816 | 0.049378 | -0.46774 |
| WC00939 | CYP63M2 | 0.004129 | 0.001228 | 0.023196 | -0.50667 |
| WC08133 | CYP512CH3 | 0.007494 | 0.004275 | 0.030186 | -0.51323 |
| WC02502 | CYP5348AD8 | 0.005719 | 0.001887 | 0.037163 | -0.5261 |
| WC07841 | CYP5035F4 | 0.091505 | 0.072293 | 0.289172 | -0.5422 |
| WC09066 | CYP5348AH1 | 0.002307 | 0.000502 | 0.033262 | -0.56805 |
| WC02604 | CYP5340A70 | 0.031686 | 0.025916 | 0.133972 | -0.56932 |
| WC08782 | CYP5350F4 | 0.00064 | 0.000258 | 0.007546 | -0.57006 |
| WC08132 | CYP512CH2 | 0.003401 | 0.002244 | 0.038473 | -0.58514 |
| WC00895 | CYP5149B2 | 0.005486 | 0.000462 | 0.162668 | -0.58584 |
| WC09952 | CYP5154A5 | 0.00564 | 0.005083 | 0.137738 | -0.6048 |
| WC06117 | CYP53D | 0.004245 | 0.004334 | 0.035403 | -0.60966 |
| WC08249 | CYP5137A8 | 0.070316 | 0.343885 | 0.147624 | -0.6223 |
| WC00627 | CYP5151A22 | 0.000631 | 0.000782 | 0.005921 | -0.62742 |
| WC04941 | CYP512P11 | 0.001334 | 0.001835 | 0.016064 | -0.63121 |
| WC00942 | CYP5362B1 | 0.024861 | 0.048027 | 0.031686 | -0.6325 |
| WC06865 | CYP5363D1 | 0.00491 | 0.004979 | 0.006302 | -0.64203 |
| WC08044 | CYP5139D33 | 0.002438 | 0.007289 | 0.074325 | -0.65453 |
| WC03429 | CYP5037BB1 | 0.002801 | 0.010896 | 0.005448 | -0.65973 |
| WC06072 | CYP5350F | 0.004678 | 0.009163 | 0.046071 | -0.68313 |
| WC00865 | CYP5150F2 | 0.004843 | 0.01209 | 0.065154 | -0.6914 |
| WC00818 | CYP5037BA | 0.002036 | 0.00568 | 0.022876 | -0.7295 |
| WC04845 | CYP512N7 | 0.035897 | 0.126745 | 0.073812 | -0.73284 |
| WC09718 | CYP5348AA4 | 0.007494 | 0.11344 | 0.05366 | -0.74703 |
| WC04299 | CYP512 | 0.002631 | 0.00455 | 0.01209 | -0.7488 |
| WC02512 | CYP5037BA1 | 0.004456 | 0.007705 | 0.020193 | -0.75125 |
| WC07283 | CYP5144L3 | 0.011359 | 0.012958 | 0.018453 | -0.76414 |
| WC04848 | CYP512N10 | 0.020054 | 0.022876 | 0.031686 | -0.77572 |
| WC08454 | CYP5037B84 | 0.003545 | 0.008144 | 0.017701 | -0.82895 |
| WC02630 | CYP5136M2 | 0.001785 | 0.04095 | 0.025208 | -0.8575 |
| WC10132 | CYP5037BB2 | 0.002339 | 0.005759 | 0.010598 | -0.88096 |
| WC06863 | CYP5357D1 | 0.00455 | 0.01176 | 0.021944 | -0.88118 |
| WC00968 | CYP5150AR2 | 0.004395 | 0.01541 | 0.025916 | -0.92852 |
| WC09932 | CYP5035F5 | 0.002064 | 0.010672 | 0.014579 | -0.98376 |
| WC10654 | CYP5346A2 | 0.006215 | 0.015843 | 0.01937 | -0.99125 |
| WC02602 | CYP5340A69 | 0.03983 | 0.099442 | 0.11908 | -0.99388 |
| WC05147 | CYP5348AA2 | 0.009486 | 0.032804 | 0.034674 | -0.99802 |

**SUPPLEMENTARY DATA**

**Supplementary Data 1. A block containing 242 gens only exit in WCLT**

**Supplementary Data 2. Genes involved in *W.cocos* genome-wide doubling events**

**Supplementary Data 3. Gene family expansion and contraction of 31 fungi**

**Supplementary Data 4. Gene family most significant expansion in *W.cocos* genome**

**Supplementary Data 5. The domains contained in significantly differentially expressed genes**

**Supplementary Data 6. CAZymes number of different family from wood rot representative fungal genomes**

**Supplementary Data 7. Identification and comparison of CYP450 genes in WCLT and WCFL**

**Supplementary Data 8. Biosynthesis gene clusters located on 7 chromosomes**

**Supplementary Data 9. Genes that may regulate the formation of sclerotium obtained by alignment**

**Supplementary Data 10. Expression of genes containing NAD and FAD related domains**

**Supplementary Data 11. Transporters in *W.cocos* genome**

**Supplementary Data 12. Transcriptional regulators in *W.cocos* genome**

**References**

1. Zhao J, Chang S-T. Monokaryotization by protoplasting heterothallic species of edible mushrooms. *World Journal of Microbiology and Biotechnology* **9**, 538-543 (1993).

2. Mehrabi R, Taga M, Aghaee M, de Wit PJGM, Kema GHJ. Karyotyping Methods for Fungi. In: *Plant Fungal Pathogens: Methods and Protocols* (eds Bolton MD, Thomma BPHJ). Humana Press (2012).

3. Simão FA, Waterhouse RM, Ioannidis P, Kriventseva EV, Zdobnov EM. BUSCO: assessing genome assembly and annotation completeness with single-copy orthologs. *Bioinformatics (Oxford, England)* **31**, 3210-3212 (2015).

4. Emms DM, Kelly S. OrthoFinder: solving fundamental biases in whole genome comparisons dramatically improves orthogroup inference accuracy. *Genome biology* **16**, 157 (2015).

5. Stamatakis A. RAxML version 8: a tool for phylogenetic analysis and post-analysis of large phylogenies. *Bioinformatics (Oxford, England)* **30**, 1312-1313 (2014).

6. Li L, Stoeckert CJ, Jr., Roos DS. OrthoMCL: identification of ortholog groups for eukaryotic genomes. *Genome research* **13**, 2178-2189 (2003).

7. De Bie T, Cristianini N, Demuth JP, Hahn MW. CAFE: a computational tool for the study of gene family evolution. *Bioinformatics (Oxford, England)* **22**, 1269-1271 (2006).

8. Floudas D*, et al.* The Paleozoic origin of enzymatic lignin decomposition reconstructed from 31 fungal genomes. *Science* **336**, 1715-1719 (2012).

9. James TY*, et al.* Polyporales genomes reveal the genetic architecture underlying tetrapolar and bipolar mating systems. *Mycologia* **105**, 1374-1390 (2013).

10. Blin K*, et al.* antiSMASH 5.0: updates to the secondary metabolite genome mining pipeline. *Nucleic acids research* **47**, W81-w87 (2019).

11. Saier MH, Jr., Reddy VS, Tsu BV, Ahmed MS, Li C, Moreno-Hagelsieb G. The Transporter Classification Database (TCDB): recent advances. *Nucleic acids research* **44**, D372-379 (2016).
